# A Domino Radical Amidation/Semipinacol Approach to All‐Carbon Quaternary Centers Bearing an Aminomethyl Group

**DOI:** 10.1002/chem.202300922

**Published:** 2023-07-24

**Authors:** Mandeep S. Dhak, Dhanarajan Arunprasath, Stephen P. Argent, James D. Cuthbertson

**Affiliations:** ^1^ GlaxoSmithKline Carbon Neutral Laboratories for Sustainable Chemistry University of Nottingham, Jubilee Campus Triumph Road Nottingham NG7 2TU UK; ^2^ School of Chemistry University of Nottingham University Park Nottingham NG7 2RD UK

**Keywords:** aminofunctionalisation, nitrogen-centred radicals, photoredox catalysis, ring expansion, semipinacol rearrangement

## Abstract

A photoredox‐mediated radical amidation ring‐expansion sequence that enables the generation of all‐carbon quaternary centers bearing a protected aminomethyl substituent is described. The methodology can be applied to both styrene and unactivated alkene substrates generating structurally diverse sp^3^‐rich amine derivatives in a concise manner.

## Graphical abstract

**A photoredox‐mediated** radical amidation semipinacol sequence enables the generation of all‐carbon quaternary centers bearing a protected aminomethyl substituent. The methodology can be applied to both styrene and unactivated alkene substrates generating structurally diverse sp^3^‐rich amine derivatives in a concise manner.

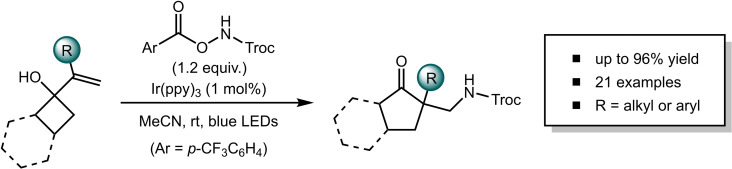

## Introduction

**Figure 1:**
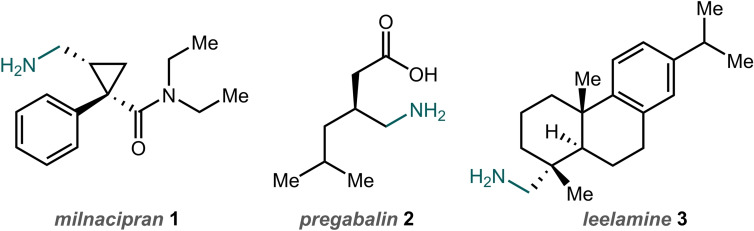
Bioactive molecules featuring the aminomethyl motif.

Nitrogen‐containing molecules are of great importance to society, with applications ranging from pharmaceuticals and agrochemicals to novel materials and surfactants.^[1]^ Of particular significance is the aminomethyl motif which is encountered in a range of synthetically valuable intermediates and building blocks, as well as various biologically active molecules such as milnacipran **1** and pregabalin **2** (Figure 1).[^2^, ^3^] Often, the aminomethyl motif can be accessed via well‐established amine synthesis methods such as nucleophilic substitution, Mannich reactions, or reductive amination.[^1^, ^4^] However, the synthesis of compounds in which this group is attached to an all‐carbon quaternary center, as found in the diterpene amine leelamine **3**,^[5]^ can be more challenging.

Arguably, the most common method used for accessing this motif is via reduction of the corresponding nitrile.[^6^, ^7^] However, the nitriles are typically prepared via alkylation reactions involving toxic alkyl halides. Given the prevalence of all‐carbon quaternary centers bearing an aminomethyl substituent, there remains a need for new synthetic methods that provide access to this motif from readily available precursors and under mild conditions.

**Figure 2:**
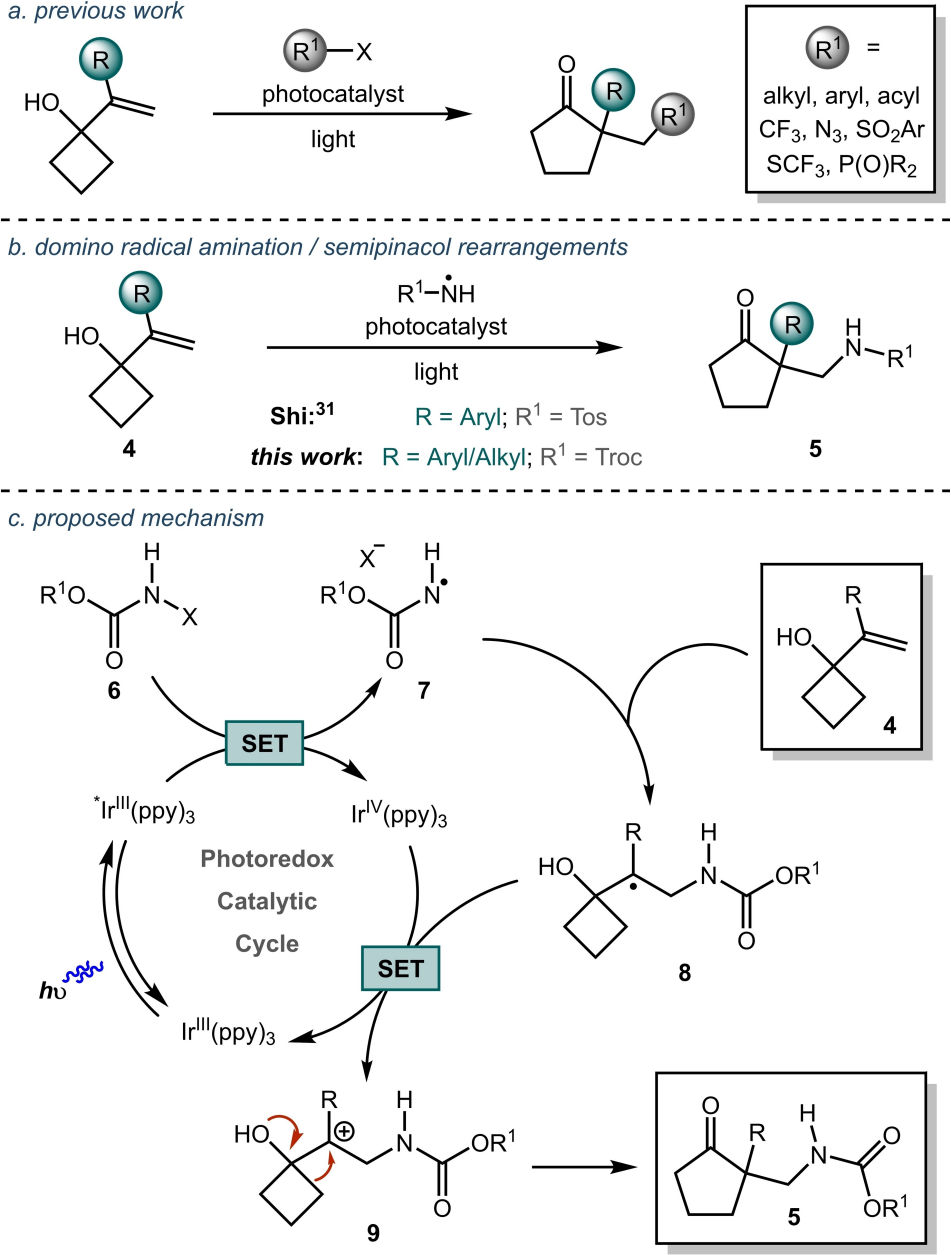
Synthesis of all‐carbon quaternary centers via domino radical addition/semipinacol rearrangements.

The semipinacol rearrangement provides an important strategy for accessing all‐carbon quaternary centers.[^8^, ^9^, ^10^, ^11^] In recent years, numerous groups have reported photoredox‐mediated radical addition/semipinacol rearrangement sequences that enable the construction of all‐carbon quaternary centers under mild conditions (Figure 2a).[^12^, ^13^, ^14^, ^15^, ^16^, ^17^, ^18^, ^19^, ^20^, ^21^, ^22^, ^23^, ^24^] However, despite significant advances in this area, examples of photoredox and non‐photoredox methods that use a semipinacol rearrangement sequence to install an aminomethyl group, or derivatives thereof, remain limited.[^25^, ^26^, ^27^, ^28^, ^29^, ^30^] Furthermore, these existing methods have a number of drawbacks such as the use of chlorinated solvents or potentially unstable/toxic azide‐based reagents. If a mild and broadly applicable amidation‐induced rearrangement process that overcomes these limitations could be developed, it would provide a valuable method for accessing synthetically valuable building blocks **5** featuring an aminomethyl substituted all‐carbon quaternary center (Figure 2b). During the course of our studies, Shi and co‐workers demonstrated such an approach for the synthesis of tosylated β‐amino (spiro)cyclic ketones from activated alkene (styrenyl) substrates.^[31]^ Additionally, a further example of a photochemical radical amidation/semipinacol reaction was recently reported by Xu and co‐workers.^[32]^ Despite these advances, to the best of our knowledge, this photoredox‐mediated domino radical amidation/semipinacol strategy has only been applied to styrene substrates. Development of a method that could be applied to unactivated alkenes would greatly expand the scope of this strategy enabling access to diverse sp^3^‐rich building blocks.

Mechanistically, we envisaged a redox‐neutral strategy analogous to that proposed by Shi and co‐workers,^[31]^ involving a photochemically generated nitrogen‐centered radical **7** (Figure 2c).[^33^, ^34^, ^35^, ^36^] Intermolecular addition of a reductively generated carbamidyl radical **7** to an alkene **4**,[^37^, ^38^, ^39^, ^40^, ^41^, ^42^, ^43^, ^44^, ^45^] would give the carbon‐centered radical **8**. Single electron oxidation of radical **8** by the oxidised photocatalyst would afford carbocation **9**, which would then undergo a semipinacol rearrangement to give the product **5**.

Herein, we report the successful development of this approach which provides a straightforward method for accessing all‐carbon quaternary centers bearing a protected aminomethyl substituent. The reaction proceeds under mild conditions and generates structurally diverse sp^3^‐rich amine derivatives from both styrene and unactivated alkene substrates.

## Results and Discussion

Our investigation began with the optimization of the reaction using styrene‐derived substrate **10 a** (Table 1), prepared in a single step via addition of styrenyl magnesium bromide to cyclobutanone (see Supporting Information for details). The readily accessible hydroxylamine‐derived amidyl radical precursors **6** used by Yu and co‐workers were employed in initial studies.^[38]^ Based on reported chemistry using similar carbamidyl radical precursors,^[38]^ we rapidly identified suitable conditions, using the highly reducing photocatalyst Ir(ppy)_3_ [(*E*_1/2_^IV/*III^)=−1.73 V versus saturated calomel electrode (SCE) in MeCN]^[46]^ (Table 1, entry 1). Minor amounts of epoxide **12** were often observed in early experiments.^[13]^ However, it was found that by stirring with acid (1 M aqueous HCl) at the end of the reaction, the undesired epoxide side‐product **12** could be converted into the desired product **11**.^[47]^ A reaction in DMSO resulted in a reduced yield of the product (Table 1, entry 2). Furthermore, no product was observed when the reaction was carried out in DCM (Table 1, entry 3). Other protecting groups could be used in place of the Troc‐group; however, the yield was lower when *N*‐Boc or *N*‐Cbz derivatives were used (Table 1, entries 4–5). Furthermore, in the case of the *N*‐Alloc derivative, multiple products were evident in the ^1^H NMR spectrum of the unpurified reaction mixture (Table 1, entry 6). The reaction was also repeated using the radical precursor employed by Shi and co‐workers which gave product **11** in a significantly reduced yield under our standard conditions (Table 1, entry 7).^[31]^ The reaction was found to be less efficient when irradiated with white LEDs instead of blue (Table 1, entry 8), or when water (5 equiv.) was present (Table 1, entry 9). Furthermore, a reaction carried out under an air atmosphere gave the product in reduced yield (entry 10). Finally, control reactions confirmed the requirement for both the photocatalyst and light (Table 1, entries 11 and 12).

**Table 1:**
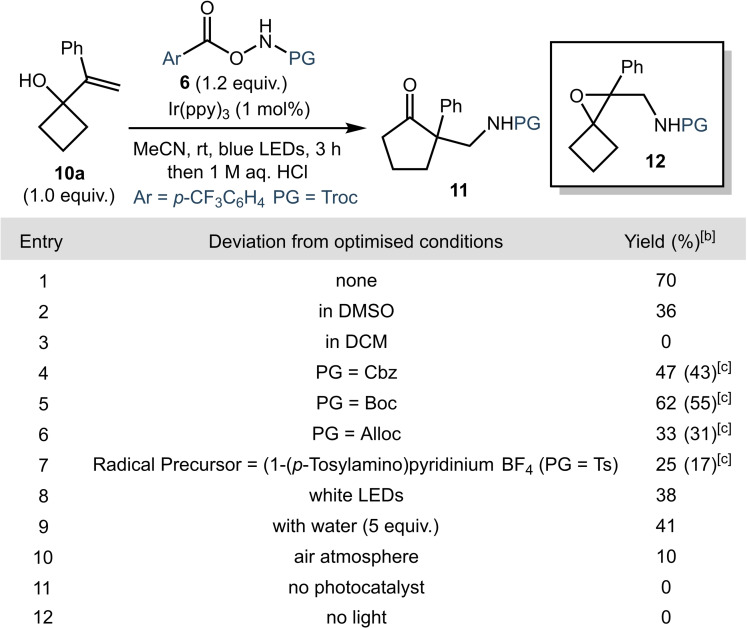
Optimization studies and control reactions.^[a]^

[a] Reactions were carried out on a 0.2 mmol scale in MeCN ([**10 a**]_0_=0.2 M). [b] Yields were determined by ^1^H NMR spectroscopy using 1,3‐benzodioxole as the internal standard. [c] Yields in parentheses refer to material isolated after purification by flash column chromatography (see Supporting Information for details).

With optimized reaction conditions in hand, we next sought to explore the scope of the process. We initially investigated a series of styrene derivatives **10** bearing different substituents on the aromatic ring (Table 2a). The unsubstituted styrene derivative used in optimization studies gave product **11 a** in 68 % yield when carried out on 0.4 mmol scale. This reaction could also be conducted on a larger scale (1.44 mmol) with no significant decrease in the isolated yield. Increasing the electron density on the aryl ring by introduction of a *p*‐*t*Bu group gave product **11 b** in excellent yield. Furthermore, an analogous substrate bearing an additional carbon in the ring was also tolerated giving the cyclohexanone derivative **11 c** in 68 % yield. Pleasingly, substrates bearing extended aromatic systems such as a naphthalene derivative and biphenyl derivative also gave the products in good to excellent yields (**11 d** and **11 e**). In addition, substrates bearing halide substituents at either the *meta*‐ or *para*‐position could also be employed in the reaction (**11 f** and **11 g**). In the case of product **11 f**, 3 points of diversification are present in the molecule which could enable further elaboration of the core structure. Substrates containing a heteroatom in the ring were next investigated. Pleasingly, when an oxetane derivative **10 h** was subjected to the optimized conditions, the product **11 h** was obtained in 40 % yield. However, in the case of the azetidine derivative **10 i** a complex mixture was obtained from which no product could be isolated. An indene derivative was also found to be a suitable substrate giving the diastereomeric products **11 j** and **11 j′** in excellent yield as a separable mixture of diastereoisomers (d.r.=3.2 : 1). Finally, the chemistry is not limited to ring expansion processes (Table 2b). A benzophenone derived substrate **13** underwent a radical amidation/1,2‐aryl migration cascade to give the ketone product **14** in 49 % yield.

**Table 2:**
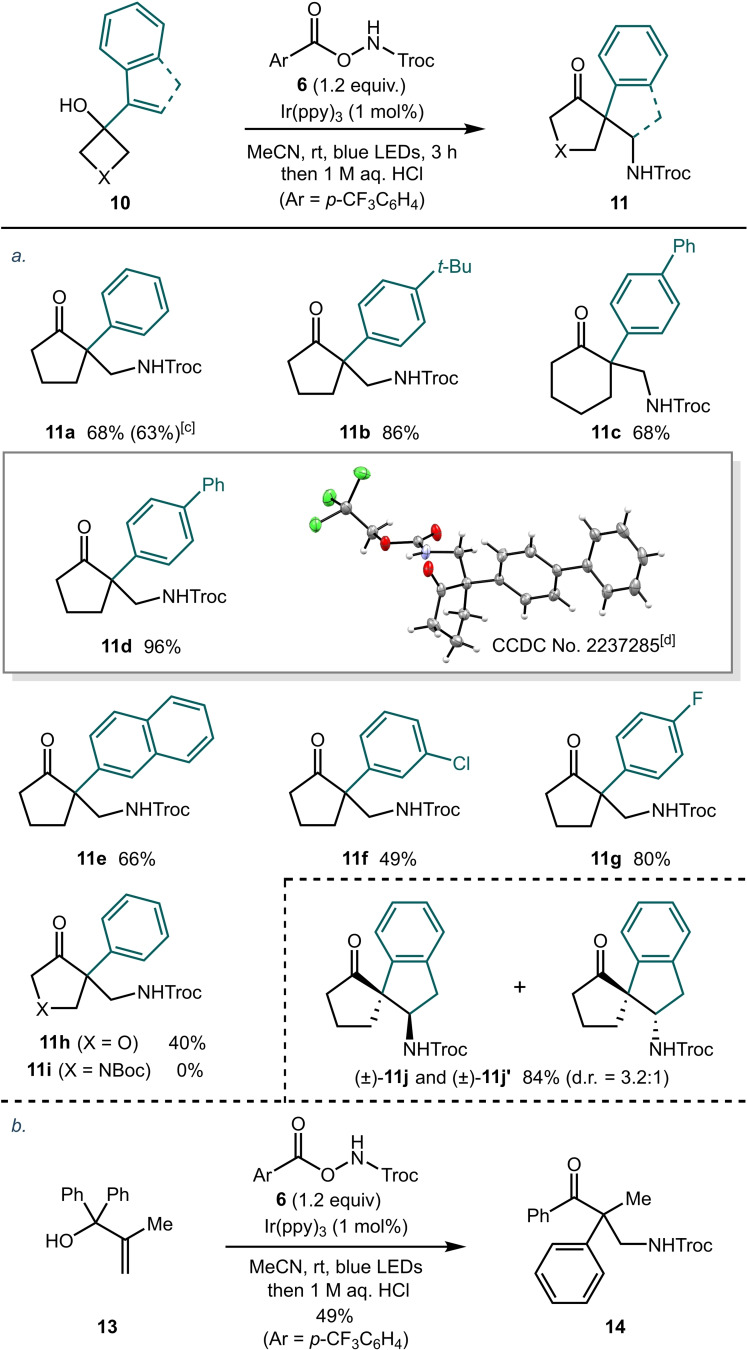
Styrene scope.^[a,b]^

[a] Reactions were carried out on a 0.4 mmol scale in MeCN ([**10**]_0_=0.2 M). [b] Yields refer to material isolated after purification by flash column chromatography. [c] Isolated yield for a reaction carried out on a 1.44 mmol scale. [d] The second molecule within the asymmetric unit has been omitted for clarity.

We next sought to extend the concept to unactivated alkene substrates **15**, which, to the best of our knowledge have not been explored in photo‐mediated radical amidation induced semipinacol reactions (Table 3). Pleasingly, under the optimized conditions, a simple propenyl‐derived substrate underwent the desired amidation ring expansion sequence to give product **16 a** in 78 % yield. Interestingly, this reactivity is not limited to carbamidyl radicals; employing the radical precursor used by Shi and co‐workers gave the *N*‐tosyl analogue of product **16 a** in 69 % yield under our standard conditions (see Supporting Information for details).^[31]^

**Table 3:**
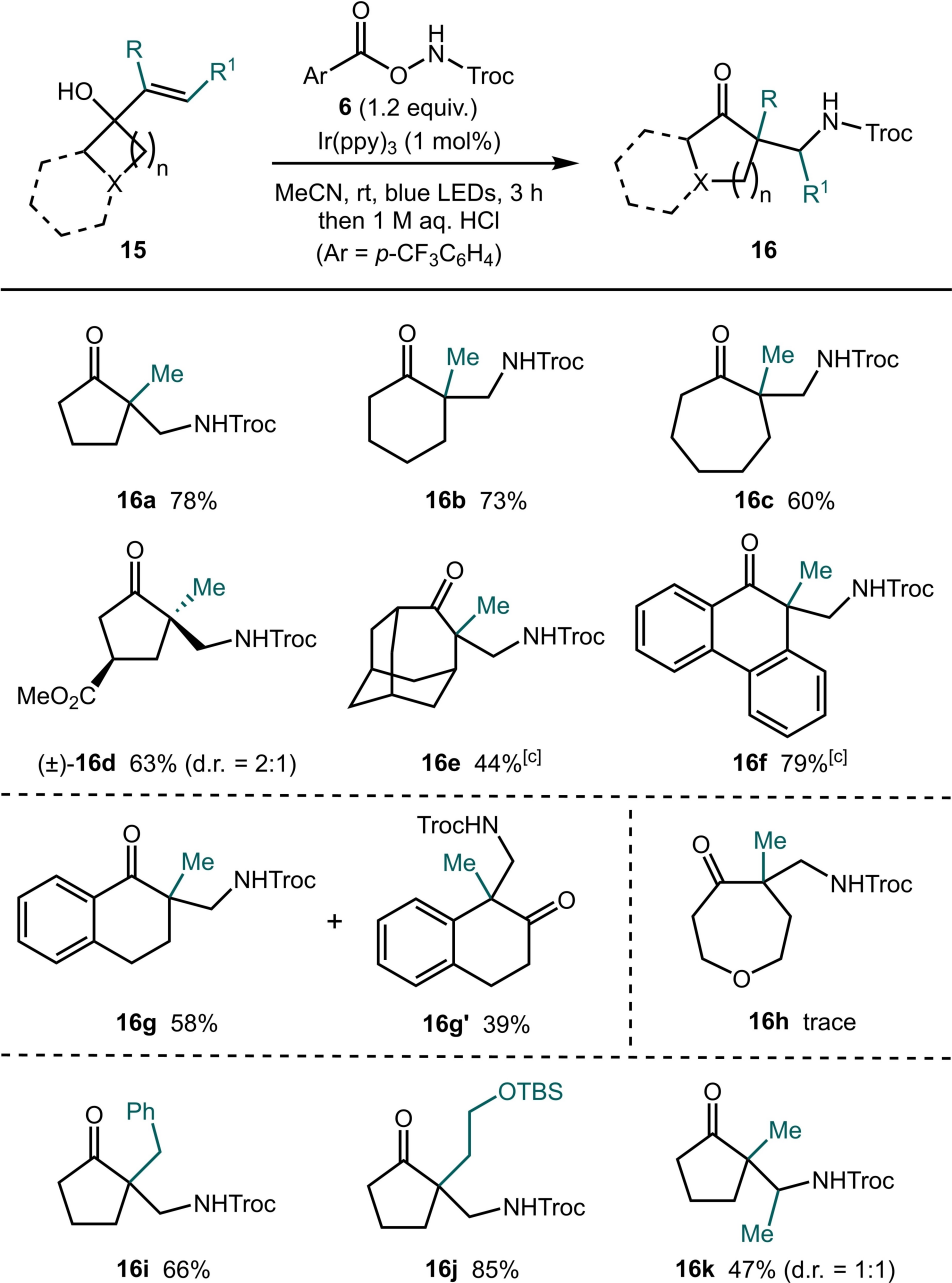
Unactivated alkene scope.^[a,b]^

[a] Reactions were carried out on a 0.4 mmol scale in MeCN ([**15**]_0_=0.2 M). [b] Yields refer to material isolated after purification by flash column chromatography. [c] Reaction time=48 h.

Reactions involving unactivated alkene substrates are not limited to the synthesis of 5‐membered rings. Substrates featuring 5 and 6‐membered rings both underwent the semipinacol rearrangement to give the corresponding cyclohexanone **16 b** and cycloheptanone **16 c** derivatives in good yield. A substrate bearing an ester substituent on the cyclobutane ring gave the product **16 d** in good yield (d.r. 2 : 1). Notably, a substrate prepared from adamantanone was also tolerated giving the caged product **16 e** in 44 % yield. Cyclic alcohols fused to aromatic rings were also suitable substrates. The fluorenone derived product **16 f** was obtained in 79 % yield. Furthermore, an indanone derived substrate gave product **16 g** in 58 % yield along with the isomeric product **16 g′** arising from migration of the phenyl ring in the semipinacol rearrangement. A heterocyclic substrate derived from tetrahydro‐4*H*‐pyran‐4‐one gave traces (<5 %) of product **16 h** when subjected to the standard conditions. However, attempts to isolate the product from the crude reaction mixture proved unsuccessful. We next sought to explore the scope of the substituents on the alkene. Pleasingly, a benzyl group and an alkyl chain bearing a TBS‐protected alcohol could both be employed in the reaction giving the products **16 i** and **16 j** in good yields. Finally, terminal alkene substrates are not a necessity; the carbamidyl radical was also found to add to a trisubstituted alkene forming product **16 k** as a mixture of diastereoisomers.

**Scheme 1:**
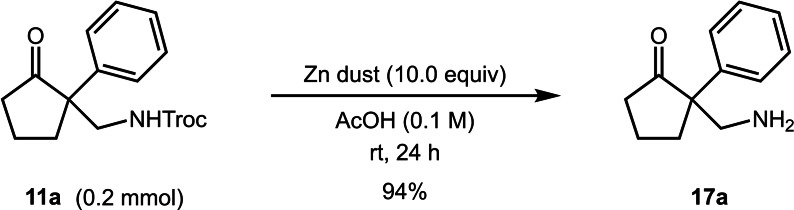
Removal of the Troc group from product **11 a**.

To conclude out studies we investigated the removal of the Troc group. Pleasingly, on treatment with zinc dust in acetic acid, the protecting group was cleanly removed from product **11 a** giving the primary amine **17 a** in excellent yield (Scheme 1).

## Conclusions

In summary, we have developed a straightforward strategy for preparing all‐carbon quaternary centers bearing a protected aminomethyl group. By merging a radical amidation with a semipinacol rearrangement, diverse sp^3^‐rich amine derivatives can be accessed in a straightforward manner. The methodology is applicable to both styrene and unactivated alkene substrates generating a range of protected amine building blocks from readily accessible precursors. Further studies exploring the radical aminofunctionalisation of alkenes are currently underway in our laboratory.

## Experimental Section

**General procedure for the photoredox mediated amidation/semipinacol rearrangement sequence**: To a mixture of the olefin substrate **10** or **15** (400 μmol, 1.00 equiv.), amidyl radical precursor **6** (183 mg, 480 μmol, 1.20 equiv.) and Ir(ppy)_3_ (2.7 mg, 4.00 μmol, 1 mol %), was added acetonitrile (2 mL). The reaction mixture was sparged with argon for 10 min, then stirred at rt whilst irradiating with blue LEDs. After 3 h, 1 M aq. HCl solution (4 mL) was added, then the reaction was stirred at rt for a further 18 h. The aqueous layer was separated and extracted with ethyl acetate (×3), then the combined organic phases were washed with 1 M aq. NaOH and brine, dried (MgSO_4_), then concentrated in vacuo. Purification by flash column chromatography gave the products **11** and **16**.

Deposition Number 2237285 (**11 d**) contains the supplementary crystallographic data for this paper. These data are provided free of charge by the joint Cambridge Crystallographic Data Centre and Fachinformationszentrum Karlsruhe Access Structures service.

## Supporting Information

Additional references cited within the Supporting Information.[^48^, ^49^, ^50^, ^51^, ^52^, ^53^, ^54^, ^55^, ^56^, ^57^, ^58^, ^59^, ^60^, ^61^, ^62^, ^63^, ^64^, ^65^, ^66^, ^67^, ^68^, ^69^, ^70^, ^71^, ^72^, ^73^, ^74^]

## Conflict of interest

The authors declare no conflict of interest.

1

## Supporting information

As a service to our authors and readers, this journal provides supporting information supplied by the authors. Such materials are peer reviewed and may be re‐organized for online delivery, but are not copy‐edited or typeset. Technical support issues arising from supporting information (other than missing files) should be addressed to the authors.

Supporting Information

## Data Availability

The data that support the findings of this study are available in the supplementary material of this article.
