# Supplementary material for: A Domino Radical Amidation/Semipinacol Approach to All‐Carbon Quaternary Centers Bearing an Aminomethyl Group
Source: Chemistry. 2023 Jul 24;29(47):e202300922. doi: 10.1002/chem.202300922 (PMC10947466; doi:10.1002/chem.202300922)
Supplement: Supplementary file 1 — Supporting Information [file CHEM-29-0-s001.pdf]

Chemistry–A European Journal

Supporting Information

A Domino Radical Amidation/Semipinacol Approach to All-Carbon Quaternary Centers Bearing an Aminomethyl Group

Mandeep S. Dhak, Dhanarajan Arunprasath, Stephen P. Argent, and James D. Cuthbertson*

Contents

1. General Experimental 2

2. Synthesis of Amidyl Radical Precursors 3

3. Substrate Synthesis: 6

3.1 Vinyl Bromides: 6

3.2 General Procedures: 6

3.3 Experimental Procedures and Characterization Data 8

4. Photoredox Mediated Amidation / Semipinacol Rearrangement 18

5. Removal of the Troc Protecting Group 33

6. Crystal Structure of Compound **11d** 34

7. References 36

8. Copies of ^{1}H, ^{13}C, and ^{19}F NMR Spectra for Novel Radical Precursors and Substrates 40

9. Copies of ^{1}H, ^{13}C, and ^{19}F NMR Spectra for all Products 46

1. General Experimental

Starting materials were obtained from commercial sources and were used without further purification unless stated otherwise. Reactions were carried out under an atmosphere of argon (unless otherwise stated), and reactions requiring anhydrous conditions were performed in oven-dried apparatus using distilled solvents. Acetonitrile and tetrahydrofuran were taken from an Inert® PureSolv MD5 solvent purification system under nitrogen. All water used was previously deionised, petrol refers to petroleum ether (b.p. 40-60 °C), and ether refers to diethyl ether. Thin layer chromatography was carried out using Merck silica gel precoated sheets SIL G/UV254, which were visualised under UV light then stained with basic potassium permanganate solution or vanillin-sulfuric acid solution. Flash column chromatography was performed using Merck silica gel 60, 35-70 µm particles as the packing agent.

^{1}H NMR, ^{13}C NMR and ^{19}F NMR spectra were acquired on Bruker Avance III 400HD or Avance III 500HD spectrometers. Chemical shifts (δ) are reported in parts per million (ppm); coupling constants (*J*) are quoted in Hertz (Hz) and are reported to the nearest 0.1 Hz. ^{1}H and ^{13}C chemical shifts are reported relative to tetramethylsilane, and are referenced to the appropriate residual solvent peaks (^{1}H NMR: $CDCl_3$ at 7.26 ppm; ^{13}C NMR: $CDCl_3$ at 77.16 ppm). ^{1}H NMR spectral data are reported as follows; chemical shift, number of protons, multiplicity and coupling constant. The following abbreviations (and combinations of) are used to label multiplicities: s (singlet), d (doublet), t (triplet), q (quartet), quint (quintet), m (multiplet), br (broad), and app (apparent). HSQC, DEPT, NOESY and COSY experiments were used to assist structural assignments.

All IR experiments were carried out on a Bruker Tensor 27 FT-IR spectrometer. High-resolution electrospray ionization time-of-flight (ESI-TOF) mass spectra were recorded using a Bruker MicroTOF II mass spectrometer. High-resolution electron impact ionization (EI) mass spectra were recorded using a JEOL AccuTOF-GCx mass spectrometer. Melting points were recorded on a Gallenkamp melting point apparatus and are uncorrected.

All photoredox reactions were performed in an EvoluChem PhotoRedOx Box™ using an EvoluChem LED 450PF (Blue: 450 nm).

2. Synthesis of Amidyl Radical Precursors

General procedure A – *N*-Aryolation of *N*-Hydroxycarbamates

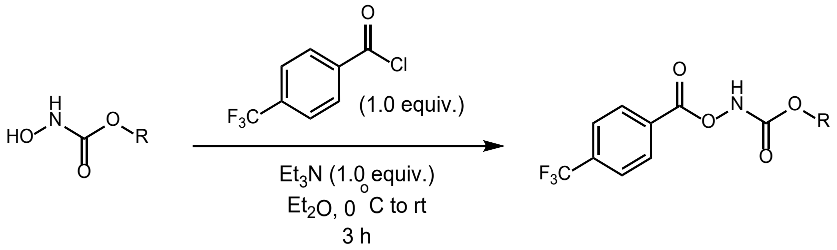

Prepared using a modified version of the procedure reported by Yu *et al.*[38] To a stirred suspension of *N*-hydroxycarbamate (1.0 equiv.) in diethyl ether (0.15 M) at 0 °C, was added triethylamine (1.0 equiv.) dropwise over 10 min. 4-(Trifluoromethyl)benzoyl chloride (1.0 equiv.) was added dropwise at the same temperature and subsequently warmed to room temperature and stirred for a further 3 h before quenching with 1.0 M aq. HCl. The phases were separated, and the organic phase was washed sequentially with H_2O, sat. aq. $NaHCO_3$, brine, dried ($MgSO_4$), filtered and concentrated *in vacuo* to afford the crude product.

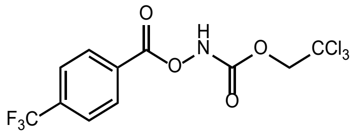

2,2,2-Trichloroethyl {[4-(trifluoromethyl)benzoyl]oxy}carbamate (6): Prepared according to general procedure A using 2,2,2-trichloroethyl hydroxycarbamate[48] (3.10 g, 15.0 mmol,) 4-trifluoromethylbenzoyl chloride (2.23 mL, 15.0 mmol), triethylamine (2.09 mL, 15.0 mmol) and diethyl ether (100 mL). Purification by flash column chromatography (SiO_2, cyclohexane:ethyl acetate – 8:2) gave the title compound **6** (5.42 g, 14.2 mmol, 95%) as a colourless crystalline solid; m.p. 96 – 98 °C; R_f = 0.32 (cyclohexane/ethyl acetate; 8:2); ^{1}H NMR (500 MHz, $CDCl_3$) δ 8.54 (1H, br. s), 8.24 (2H, d, *J* = 8.3 Hz), 7.78 (2H, d, *J* = 8.3 Hz), 4.86 (2H, s); ^{13}C{^{1}H} NMR (126 MHz, $CDCl_3$) δ 164.5, 154.5, 136.0 (q, *J* = 32.9 Hz, ArC), 130.6, 129.7, 126.0 (q, *J* = 3.7 Hz), 125.1 (q, *J* = 273 Hz), 94.5, 75.4; ^{19}F NMR (376 MHz, $CDCl_3$) δ –63.4.

Data are consistent with those reported in the literature.[38]

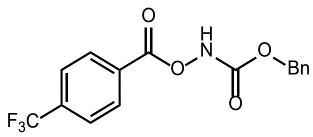

Benzyl {[4-(trifluoromethyl)benzoyl]oxy}carbamate (S1): Prepared according to general procedure A using *N*-(benzyloxycarbonyl)hydroxylamine (1.17 g, 7.00 mmol,) 4-trifluoromethylbenzoyl chloride (1.04 mL, 7.00 mmol), triethylamine (976 μL, 7.00 mmol) and diethyl ether (46 mL). Purification by

flash column chromatography (SiO_2, cyclohexane:ethyl acetate – 8.5:1.5) gave the title compound **S1** (1.54 g, 4.5 mmol, 65%) as a colourless crystalline solid; m.p. 112 – 114 °C; ^{1}H NMR (400 MHz, $CDCl_3$) δ 8.51 (1H, s), 8.23 – 8.17 (2H, m), 7.75 (2H, d, *J* = 8.2 Hz), 7.41 – 7.30 (5H, m), 5.26 (2H, s); ^{13}C{^{1}H} NMR (101 MHz, $CDCl_3$) δ 164.8, 156.4, 135.8 (q, *J* = 32.9 Hz) 135.0, 130.5, 130.1, 128.8, 128.8, 128.5, 125.9 (q, *J* = 3.7 Hz), 123.5 (q, *J* = 273.1 Hz), 68.7; ^{19}F NMR (376 MHz, $CDCl_3$) δ −63.3. Data are consistent with those reported in the literature.[39]

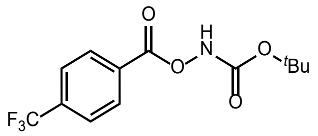

***tert*-Butyl {[4-(trifluoromethyl)benzoyl]oxy}carbamate (S2):** Prepared according to general procedure A using *N*-Boc-hydroxylamine (1.07 g, 8.00 mmol,) 4-trifluoromethylbenzoyl chloride (1.19 mL, 8.00 mmol), triethylamine (1.12 mL, 8.00 mmol) and diethyl ether (50 mL). Purification by flash column chromatography (SiO_2, cyclohexane:ethyl acetate – 8.5:1.5) gave the title compound **S2** (2.10 g, 6.9 mmol, 86%) as a colourless crystalline solid; m.p. 83 – 85 °C; ^{1}H NMR (400 MHz, $CDCl_3$) δ 8.26 – 8.19 (2H, m), 8.17 (1H, s), 7.76 (2H, d, *J* = 8.2 Hz), 1.52 (9H, s); ^{13}C{^{1}H} NMR (101 MHz, $CDCl_3$) δ 165.1, 155.5, 135.6 (q, *J* = 32.9 Hz), 130.5, 130.4, 125.8 (q, *J* = 3.7 Hz), 123.5 (d, *J* = 272.9 Hz), 83.8, 28.1; ^{19}F NMR (376 MHz, $CDCl_3$) δ −63.4.
Data are consistent with those reported in the literature.[49]

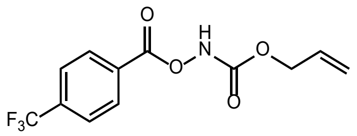

Allyl {[4-(trifluoromethyl)benzoyl]oxy}carbamate (S3): Prepared according to general procedure A using allyl hydroxycarbamate[50] (1.05 g, 9.00 mmol), 4-(trifluoromethyl)benzoyl chloride (1.34 mL, 9.00 mmol), triethylamine (1.25 mL, 9.00 mmol) and diethyl ether (60 mL, 0.15 M). Purification by flash column chromatography (SiO_2, cyclohexane:ethyl acetate – 8.5:1.5) gave the title compound **S3** (2.03 g, 7.02 mmol, 78%) as a colourless crystalline solid; m.p. 66 – 68 °C; R_f = 0.35 (cyclohexane/ethyl acetate; 4:1); υ_{max}/cm^{-1} (thin film) 3212, 2949, 1769, 1738, 1715, 1482, 1324, 1172, 1119, 1068; ^{1}H NMR (400 MHz, $CDCl_3$) δ 8.60 (1H, s), 8.23 – 8.16 (2H, m), 7.74 (2H, d, *J* = 8.2 Hz), 5.92 (1H, ddt, *J* = 17.2, 10.3, 5.7 Hz), 5.35 (1H, dq, *J* = 17.2, 1.5 Hz), 5.26 (1H, dq, *J* = 10.4, 1.2 Hz), 4.71 (2H, dt, *J* = 5.8, 1.4 Hz); ^{13}C{^{1}H} NMR (101 MHz, $CDCl_3$) δ 164.8, 156.3, 135.7 (q, *J* = 32.9 Hz), 131.4, 130.5, 130.1, 125.9 (q, *J* = 3.8 Hz), 123.5 (q, *J* = 272.9 Hz), 119.3, 67.5; ^{19}F NMR (376 MHz, $CDCl_3$) δ −63.4; HRMS (ESI) m/z: [M + Na]$^+$ calcd for $C_{12}H_{10}F_3NNaO_4$, 312.0454; found, 312.0456.

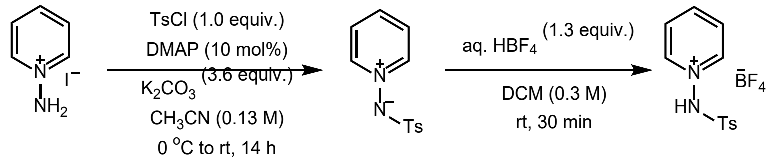

1-[(4-Methylphenyl)sulfonamide]pyridin-1-ium tetrafluoroborate (S5): Prepared using the two step procedure reported by Koike and Akita.[51]

1-(*p*-Toluenesulfonylamino)-pyridinium ylide (S4): To a stirred solution of 1-aminopyridinium iodide (1.11 g, 5.0 mmol, 1.0 equiv.) in acetonitrile (39 mL, 0.13 M) was added DMAP (61.1 mg, 0.5 mmol, 0.1 equiv.) and K_2CO_3 (2.49 g, 18.0 mmol, 3.6 equiv.) at 0 °C under N_2. The reaction was allowed to warm to room temperature and stirred for 14 h. The solids were filtered off then the filtrate was concentrated *in vacuo*. Purification by flash column chromatography (SiO_2, DCM:MeOH – 19:1) gave 1-(*p*-toluenesulfonylamino)-pyridinium ylide **S4** (1.03 g, 4.14 mmol, 83%) as a colourless solid; R_f = 0.46 (DCM/MeOH; 9:1); ^1H NMR (400 MHz, $CDCl_3$) δ 8.48 – 8.41 (2H, m), 7.98 (1H, td, *J* = 7.7, 1.3 Hz), 7.59 (4H, dd, *J* = 7.8, 6.2 Hz), 7.15 (2H, d, *J* = 7.9 Hz), 2.34 (3H, s); $^{13}C\{^1H\}$ NMR (101 MHz, $CDCl_3$) δ 145.3, 141.7, 138.8, 138.6, 129.4, 127.2, 126.8, 21.5.

Data are consistent with those reported in the literature.[51]

1-[(4-Methylphenyl)sulfonamide]pyridin-1-ium tetrafluoroborate (S5): To a stirred solution of 1-(*p*-toluenesulfonylamino)-pyridinium ylide **S4** (993 mg, 4.0 mmol, 1.0 equiv.) in DCM (13.5 mL, 0.3 M) was added aq. HBF_4 solution (50% wt. H_2O; 0.65 mL, 5.2 mmol, 1.3 equiv.) at room temperature. The reaction was stirred for 30 min then the solids were filtered and subsequently washed with diethyl ether (20 mL) and pentane (20 mL), and dried under high vac to give the title compound **S5** (1.28 g, 3.8 mmol, 96%) as a colourless solid; m.p. 220 – 222 °C; ^1H NMR (400 MHz, CD_3CN) δ 8.63 (t, *J* = 7.9 Hz, 1H), 8.37 (d, *J* = 6.2 Hz, 2H), 8.03 (t, *J* = 7.1 Hz, 2H), 7.63 – 7.56 (m, 2H), 7.46 (d, *J* = 8.1 Hz, 2H), 2.47 (s, 3H); $^{13}C\{^1H\}$ NMR (101 MHz, CD_3CN) δ 149.4, 149.0, 146.4, 131.9, 130.7, 130.4, 129.7, 21.9.

Data are consistent with those reported in the literature.[51]

3. Substrate Synthesis:

3.1 Vinyl Bromides:

Vinyl bromides were synthesized according to known procedures,[52] and were used without purification. ^{1}H NMR spectroscopic data for the unpurified vinyl bromides were consistent with those previously reported.

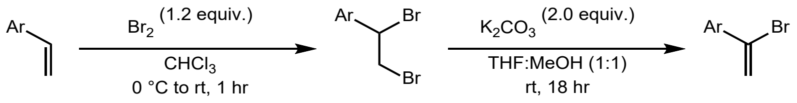

3.2 General Procedures:

General procedure B – Synthesis of styrene substrates using organolithium reagents:[15]

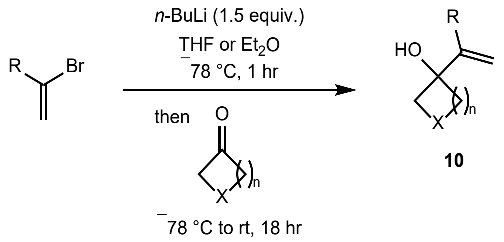

To a solution of vinyl bromide (1.50 equiv.) in THF (0.5 M) cooled to −78 °C, was added *n*-BuLi (1.50 equiv.) dropwise, then the reaction mixture was left to stir at −78 °C for 1 h. A solution of cyclic ketone (1.00 equiv.) in THF (0.5 M) was added dropwise, then the reaction mixture was warmed to room temperature and left to stir for a further 18 h. The reaction was quenched with sat. aq. NH_4Cl (12 mL), then the aqueous layer was separated and extracted using ethyl acetate (3 × 20 mL). The combined organic phases were dried ($MgSO_4$), filtered and concentrated *in vacuo* to afford the crude product.

General procedure C – Synthesis of substrates using Grignard reagents:[15]

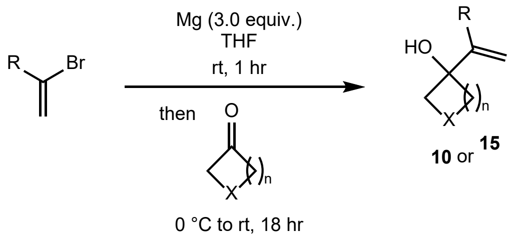

To a suspension of Mg (3.00 equiv.) in THF (1.2 M), was added vinyl bromide (1.00 equiv.) as a solution in THF (1.2 M), then the suspension was left to stir at room temperature for 1 h. The suspension was cooled to 0 °C, then a solution of cyclic ketone (1.25 equiv.) in THF (1.2 M) was added dropwise. The reaction mixture was warmed to room temperature and left to stir for a further 18 h. The reaction was quenched with sat. aq. NH_4Cl (12 mL) then the aqueous phase was separated and extracted using DCM

(3 × 30 mL). The combined organic phases were washed with water (20 mL) and brine (20 mL), dried ($MgSO_4$), filtered and concentrated *in vacuo* to afford the crude product.

General procedure D – Synthesis of unactivated alkene substrates using Grignard reagents: [53]

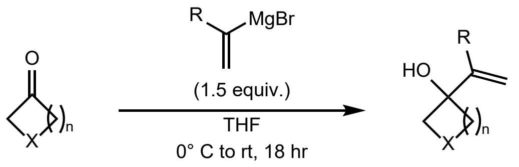

15

To a solution of alkenyl magnesium bromide (1.50 eq.) cooled to 0 °C, was added a solution of alkene (1.00 eq.) in THF (0.5 M) dropwise, then the reaction mixture was warmed to room temperature and left to stir for a further 18 h. The reaction mixture was quenched using sat. aq. NH_4Cl (10 mL) and the aqueous phase was extracted using ethyl acetate (3 × 30 mL). The combined organic phases were concentrated *in vacuo* to yield the crude product.

3.3 Experimental Procedures and Characterization Data

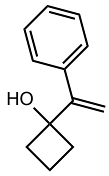

1-(1-Phenylvinyl)cyclobutanol (10a): Prepared according to general procedure B using α-bromostyrene (1.95 mL, 15.0 mmol) in THF (30 mL), *n*-BuLi (2.5 M in hexanes, 6.00 mL, 15.0 mmol) and cyclobutanone (750 µL, 10.0 mmol) in THF (20 mL). Purification by flash column chromatography (SiO_2, cyclohexane:ethyl acetate – 4:1) gave the title compound **10a** (1.43 g, 8.20 mmol, 82%) as a yellow oil; R_f = 0.30 (cyclohexane/ethyl acetate, 4:1); ^1H NMR (500 MHz, $CDCl_3$) δ 7.51 – 7.46 (2H, m), 7.35 – 7.28 (3H, m), 5.38 (1H, br. s), 5.36 (1H, br. s), 2.51 – 2.44 (2H, m), 2.29 – 2.21 (2H, m), 2.04 – 1.94 (2H, m), 1.67 – 1.60 (1H, m); $^{13}C\{^1H\}$ NMR (126 MHz, $CDCl_3$) δ 152.5, 139.2, 128.3, 127.8, 127.7, 113.0, 78.2, 35.8, 13.5.

Data are consistent with those reported in the literature.[54]

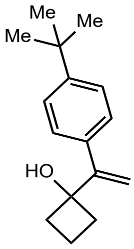

1-{1-[4-(*tert*-Butyl)phenyl]vinyl}cyclobutan-1-ol (10b): Prepared according to general procedure B using 1-(1-bromovinyl)-4-(*tert*-butyl)benzene[29] (2.94 g, 12.3 mmol) in THF (25 mL), *n*-BuLi (2.5 M in hexanes, 4.92 mL, 12.3 mmol) and cyclobutanone (620 µL, 8.19 mmol) in THF (27 mL) to afford the crude product. Purification by flash column chromatography (SiO_2, cyclohexane:ethyl acetate – 20:1) gave the title compound **10b** (1.35 g, 5.85 mmol, 71%) as a colourless oil; R_f = 0.38 (cyclohexane/ethyl acetate, 8:2); ^1H NMR (400 MHz, $CDCl_3$) δ 7.45 – 7.40 (2H, m), 7.37 – 7.32 (2H, m), 5.36 (1H, br. s), 5.35 (1H, br. s), 2.55 – 2.45 (2H, m), 2.31 – 2.22 (2H, m), 2.03 – 1.93 (2H, m), 1.70 – 1.58 (1H, m), 1.33 (9H, s); $^{13}C\{^1H\}$ NMR (101 MHz, $CDCl_3$) δ 152.0, 150.7, 136.0, 127.3, 125.3, 112.2, 78.3, 35.9, 34.65, 31.5, 13.5.

Data are consistent with those reported in the literature.[54]

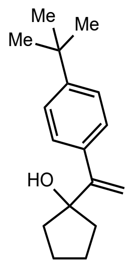

1-{1-[4-(*tert*-Butyl)phenyl]vinyl}cyclopentan-1-ol (10c): Prepared according to general procedure B using 1-(1-bromovinyl)-4-(*tert*-butyl)benzene[29] (2.77 g, 11.6 mmol) in THF (23 mL), *n*-BuLi (2.5 M in hexanes, 4.64 mL, 11.6 mmol) and cyclopentanone (690 µL, 7.70 mmol) in THF (15 mL) to afford the crude product. Purification by flash column chromatography (SiO_2, cyclohexane:ethyl acetate – 20:1) gave the title compound **10c** (1.39 g, 5.69 mmol, 74%) as a colourless crystalline solid; m.p. 101 – 103 °C; R_f = 0.20 (cyclohexane/ethyl acetate, 20:1); ^{1}H NMR (400 MHz, $CDCl_3$) δ 7.34 – 7.28 (4H, m), 5.40 (1H, d, J= 1.4 Hz), 5.08 (1H, d, J= 1.4 Hz), 1.94 – 1.84 (4H, m), 1.83 – 1.76 (2H, m) 1.72 – 1.62 (2H, m), 1.33 (9H, s); $^{13}C\{^1H\}$ NMR (101 MHz, $CDCl_3$) δ 154.8, 150.0, 138.7, 128.2, 124.9, 113.0, 84.3, 39.4, 34.6, 31.5, 23.5.

Data are consistent with those reported in the literature.[55]

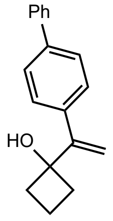

1-{1-[(1,1'-Biphenyl)-4-yl]vinyl}cyclobutan-1-ol (10d): Prepared according to general procedure C using 4-(1-bromovinyl)-1,1'-biphenyl[29] (2.54 g, 9.80 mmol) in THF (8 mL), Mg (712 mg, 29.3 mmol) in THF (24 mL) and cyclobutanone (920 µL, 12.2 mmol) in THF (10 mL). Purification by flash column chromatography (SiO_2, cyclohexane:ethyl acetate – 20:1) gave the title compound **10d** (668 mg, 2.67 mmol, 27%) a yellow oil; R_f = 0.45 (cyclohexane/ethyl acetate, 6:4); ^{1}H NMR (400 MHz, $CDCl_3$) δ 7.65 – 7.55 (6H, m), 7.51 – 7.43 (2H, m), 7.43 – 7.33 (1H, m), 5.43 (1H, s), 5.40 (1H, s), 2.60 – 2.46 (2H, m), 2.32 – 2.24 (2H, m), 2.11 – 1.99 (1H, m), 1.98 (1H, s), 1.75 – 1.61 (1H, m); $^{13}C\{^1H\}$ NMR (101 MHz, $CDCl_3$) δ 152.0, 140.9, 140.5, 138.1, 128.9, 128.1, 127.5, 127.2, 127.0, 113.0, 78.3, 35.9, 13.6.

Data are consistent with those reported in the literature.[29]

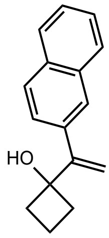

1-[1-(Naphthalen-2-yl)vinyl]cyclobutan-1-ol (10e): Prepared according to general procedure C using 2-(1-bromovinyl)naphthalene[29] (1.93 g, 8.29 mmol) in THF (7 mL), Mg (605 mg, 24.9 mmol) in THF (21 mL) and cyclobutanone (770 µL, 10.4 mmol) in THF (9 mL). Purification by flash column chromatography (SiO_2, cyclohexane:ethyl acetate – 20:1) gave the title compound **10e** (530 mg, 2.37 mmol, 29%) as a yellow oil; R_f = 0.40 (cyclohexane/ethyl acetate, 6:4); ^{1}H NMR (400 MHz, $CDCl_3$) δ 7.97 (1H, s), 7.91 – 7.76 (3H, m), 7.63 (1H, dd, *J*= 8.5, 1.8 Hz), 7.54 – 7.42 (2H, m), 5.53 – 5.45 (2H, m), 2.61 – 2.46 (2H, m), 2.38 – 2.29 (2H, m), 2.12 – 1.94 (2H, m), 1.75 – 1.56 (1H, m); ^{13}C{^{1}H} NMR (101 MHz, $CDCl_3$) δ 152.6, 136.6, 133.4, 132.9, 128.4, 127.8, 127.7, 126.6, 126.2, 126.1, 126.0, 113.5, 78.4, 35.9, 13.6.

Data are consistent with those reported in the literature.[29]

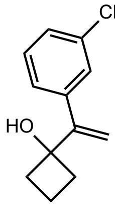

1-[1-(3-Chlorophenyl)vinyl]cyclobutan-1-ol (10f): Prepared according to general procedure C using 1-(1-bromovinyl)-3-chlorobenzene[56] (2.10 g, 9.64 mmol) in THF (8 mL), Mg (703 mg, 28.9 mmol) in THF (24 mL) and cyclobutanone (904 µL, 12.1 mmol) in THF (10 mL). Purification by flash column chromatography (SiO_2, cyclohexane:ethyl acetate – 20:1) gave the title compound **10f** (260 mg, 1.24 mmol, 12%) as a yellow oil; R_f = 0.48 (cyclohexane/ethyl acetate, 6:4); ^{1}H NMR (400 MHz, $CDCl_3$) δ 7.54 – 7.47 (1H, m), 7.44 – 7.36 (1H, m), 7.31 – 7.24 (2H, m), 5.43 (1H, s), 5.40 (1H, s), 2.52 – 2.41 (2H, m), 2.29 – 2.18 (2H, m), 2.07 – 1.95 (1H, m), 1.92 (1H, br. s), 1.71 – 1.58 (1H, m); ^{13}C{^{1}H} NMR (101 MHz, $CDCl_3$) δ 151.5, 141.2, 134.1, 129.5, 127.9, 127.7, 125.9, 114.1, 78.1, 35.8, 13.5.

Data are consistent with those reported in the literature.[15]

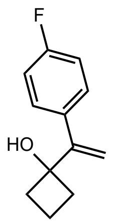

1-[1-(4-Fluorophenyl)vinyl]cyclobutan-1-ol (10g): Prepared according to general procedure B using 1-(1-bromovinyl)-4-fluorobenzene[29] (1.79 g, 8.92 mmol) in THF (18 mL), *n*-BuLi (2.5 M in hexanes, 3.60 mL, 8.92 mmol) and cyclobutanone (450 µL, 5.95 mmol) in THF (12 mL). Purification by flash column chromatography (SiO_2, cyclohexane:ethyl acetate – 20:1) gave the title compound **10g** (290 mg, 1.51 mmol, 25%) as a pale yellow oil; R_f = 0.30 (cyclohexane/ethyl acetate, 4:1); ^{1}H NMR (400 MHz, $CDCl_3$) δ 7.50 – 7.41 (2H, m), 7.05 – 6.95 (2H, m), 5.36 (1H, s), 5.32 (1H, s), 2.50 – 2.38 (2H, m), 2.27 – 2.16 (2H, m), 2.06 – 1.92 (1H, m), 1.85 (1H, br. s), 1.68 – 1.58 (1H, m); $^{13}C\{^1H\}$ NMR (101 MHz, $CDCl_3$) 162.5 (d, *J* = 246.1 Hz), 151.5, 135.2 (d, *J* = 3.5 Hz), 129.4 (d, J = 7.8 Hz), 115.2 (d, *J* = 21.0 Hz), 113.0, 78.2, 35.8, 13.5; ^{19}F NMR (376 MHz, $CDCl_3$) δ −115.08.

Data are consistent with those reported in the literature.[54]

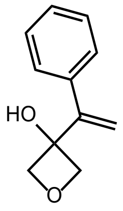

3-(1-Phenylvinyl)oxetan-3-ol (10h): Prepared according to general procedure B using α-bromostyrene (200 µL, 1.50 mmol) in THF (3 mL), *n*-BuLi (2.5 M in hexanes, 600 µL, 1.50 mmol) and 3-oxetanone (65.0 µL, 1.00 mmol) in THF (200 µL). Purification by flash column chromatography (SiO_2, cyclohexane:ethyl acetate – 10:1) gave the title compound **10h** (100 mg, 567 µmol, 56%) as a yellow oil; R_f = 0.17 (cyclohexane/ethyl acetate, 6:4); ^{1}H NMR (500 MHz, $CDCl_3$) δ 7.40 – 7.31 (5H, m), 5.59 (1H, s), 5.40 (1H, s), 4.91 (2H, dd, *J* = 7.0, 0.8 Hz), 4.79 (2H, dd, *J* = 7.0, 0.8 Hz); $^{13}C\{^1H\}$ NMR (126 MHz, $CDCl_3$) δ 149.1, 137.4, 128.8, 128.3, 126.9, 114.8, 83.4, 77.2.

Data are consistent with those reported in the literature.[29]

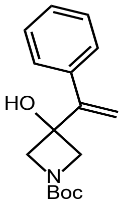

***tert*-Butyl 3-hydroxy-3-(1-phenylvinyl)azetidine-1-carboxylate (10i):** Prepared according to general procedure B using α-bromostyrene (200 µL, 1.50 mmol) in THF (3 mL), *n*-BuLi (2.5 M in hexanes,

600 µL, 1.50 mmol) and 1-boc-3-azetidinone (172 mg, 1.00 mmol) in THF (200 µL). Purification by flash column chromatography (SiO_2, cyclohexane:ethyl acetate – 10:1) gave the title compound **10i** (116 mg, 421 µmol, 42%) as a colourless crystalline solid; m.p. 97 – 99 °C (lit.[29] 95 – 110 °C); R_f = 0.22 (cyclohexane/ethyl acetate, 8:2); ^{1}H NMR (500 MHz, $CDCl_3$) δ 7.41 – 7.35 (2H, m), 7.35 – 7.23 (3H, m), 5.50 (1H, s), 5.38 (1H, s), 4.21 (2H, d, *J* = 9.1 Hz), 4.03 (2H, d, *J* = 9.1 Hz), 1.40 (9H, s); ^{13}C{^{1}H} NMR (126 MHz, $CDCl_3$) δ 156.4, 149.5, 137.7, 128.5, 128.0, 127.0, 114.8, 79.8, 72.4, 69.8, 61.8, 28.2.

Data are consistent with those reported in the literature.[29]

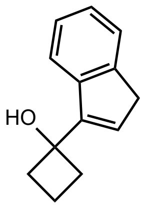

1-(1*H*-Inden-3-yl)cyclobutan-1-ol (10j): To a solution of indene (700 µL, 6.00 mmol) in diethyl ether (12 mL) cooled to −78 °C, was added *n*-BuLi (2.5 M in hexanes, 2.40 mL, 6.00 mmol) dropwise, then the reaction mixture was left to stir at −78 °C for 1 h. A solution of cyclobutanone (300 µL, 4.00 mmol) was added dropwise, then the reaction mixture was warmed to room temperature and left to stir for a further 18 h. The reaction was quenched with sat. aq. NH_4Cl (10 mL), then the aqueous layer was separated and extracted using diethyl ether (3 × 20 mL). The combined organic phases were washed with brine (50 mL), dried ($MgSO_4$), filtered and concentrated *in vacuo*. Purification by flash column chromatography (SiO_2, cyclohexane:ethyl acetate – 9:1 → 8:2) gave the title compound **10j** (570 mg, 3.05 mmol, 76%) as a colourless crystalline solid; m.p. 105 – 107 °C (lit.[57] 97 – 98 °C); R_f = 0.20 (cyclohexane/ethyl acetate, 9:1); ^{1}H NMR (500 MHz, $CDCl_3$) δ 7.59 (1H, d, *J* = 7.5 Hz), 7.49 (1H, d, *J* = 7.4 Hz), 7.30 (1H, dd, *J* = 7.5, 1.0 Hz), 7.23 (1H, *t*, J = 7.4 Hz), 6.46 (1H, t, *J* = 2.0 Hz), 3.41 (2H, d, *J* = 2.0 Hz), 2.64 – 2.55 (2H, m), 2.45 – 2.32 (2H, m), 2.06 (1H, br. s), 1.98 – 1.88 (1H, m), 1.70 – 1.57 (1H, m); ^{13}C{^{1}H} NMR (126 MHz, $CDCl_3$) δ 147.4, 145.2, 142.8, 128.3, 126.1, 125.0, 124.2, 121.7, 74.1, 37.7, 35.7, 13.4.

^{1}H and ^{13}C NMR Spectroscopic data are consistent with those reported in the literature.[58]

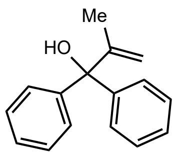

2-Methyl-1,1-diphenylprop-2-en-1-ol (13): Prepared according to general procedure D using isopropenylmagnesium bromide solution (0.5 M in THF, 9.00 mL, 4.50 mmol) and benzophenone (547 mg, 3.00 mmol) in THF (6 mL). Purification by flash column chromatography (SiO_2,

cyclohexane:diethyl ether – 9:1) gave the title compound **13** (333 mg, 1.48 mmol, 49%) as a colourless oil; R_f = 0.22 (cyclohexane/diethyl ether; 9:1); υ_{max}/cm^{-1} 3473, 1445, 1162, 1022, 904, 756, 697; ^{1}H NMR (400 MHz, $CDCl_3$) δ 7.39 – 7.24 (10H, m), 5.14 (1-H, s), 4.73 (1H, s), 2.44 (1H, br. s), 1.80 (3H, s); ^{13}C{^{1}H} NMR (101 MHz, $CDCl_3$) δ 149.2, 145.0, 128.1, 127.8, 127.4, 115.7, 83.1, 20.2.

Data are consistent with those reported in the literature. [59]

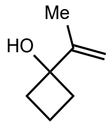

1-(Prop-1-en-2-yl)cyclobutan-1-ol (15a): Prepared according to general procedure C using 2-bromopropene (1.34 mL, 15.0 mmol) in THF (12.5 mL), Mg (1.10 g, 45.0 mmol) in THF (37.5 mL) and cyclobutanone (1.40 mL, 18.8 mmol) in THF (16 mL). Purification by flash column chromatography (SiO_2, petrol:diethyl ether – 8:2) gave the title compound **15a** (420 mg, 3.74 mmol, 25%) as a yellow oil; R_f = 0.29 (petrol/diethyl ether, 8:2); ^{1}H NMR (400 MHz, $CDCl_3$) δ 5.04 – 4.95 (1H, m), 4.86 (1H, t, J = 1.4 Hz), 2.40 – 2.28 (2H, m), 2.12 – 1.98 (2H, m), 1.96 – 1.82 (1H, m), 1.80 (3H, s), 1.66 (1H, br. s), 1.63 – 1.47 (1H, m); ^{13}C{^{1}H} NMR (101 MHz, $CDCl_3$) δ 148.0, 109.7, 78.1, 34.5, 17.6, 13.0.

Data are consistent with those reported in the literature.[57]

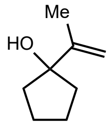

1-(Prop-1-en-2-yl)cyclopentan-1-ol (15b): Prepared according to general procedure D using isopropenylmagnesium bromide solution (0.5 M in THF, 9 mL, 4.50 mmol) and cyclopentanone (270 µL, 3.00 mmol) in THF (6 mL). Purification by flash column chromatography (SiO_2, cyclohexane:ethyl acetate – 9:1) gave the title compound **15b** (240 mg, 1.90 mmol, 63%) as a yellow oil; R_f = 0.42 (cyclohexane/ethyl acetate, 9:1); υ_{max}/cm^{-1} (thin film) 3386, 2960, 2872, 1186, 1003, 897; ^{1}H NMR (400 MHz, $CDCl_3$) δ 5.02 (1H, d, J = 1.6 Hz), 4.80 (1H, d, J = 1.6 Hz), 1.93 – 1.75 (7H, m), 1.75 – 1.58 (4H, m); ^{13}C{^{1}H} NMR (101 MHz, $CDCl_3$) δ 149.9, 109.3, 84.5, 38.6, 23.9, 19.6.

Data are consistent with those reported in the literature.[60]

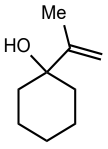

1-(Prop-1-en-2-yl)cyclohexan-1-ol (15c): Prepared according to general procedure D using isopropenylmagnesium bromide solution (0.5 M in THF, 9.00 mL, 4.50 mmol) and cyclohexanone (310 µL, 3.00 mmol) in THF (6 mL). Purification by flash column chromatography (SiO_2, cyclohexane:diethyl ether – 9:1) gave the title compound **15c** (301 mg, 2.15 mmol, 72%) as a colourless oil; R_f = 0.46 (cyclohexane/diethyl ether, 9:1); ^{1}H NMR (400 MHz, $CDCl_3$) δ 5.01 (1H, s), 4.81 (1H, s), 1.80 (3H, s), 1.71 – 1.48 (10H, m); ^{13}C{^{1}H} NMR (101 MHz, $CDCl_3$) δ 152.1, 109.4, 73.8, 36.0, 25.8, 22.2, 19.1.

Data are consistent with those reported in the literature.[61]

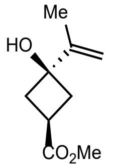
(±)-**15d**

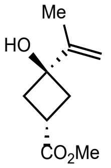
(±)-**15d'**

Methyl 3-hydroxy-3-(prop-1-en-2-yl)cyclobutane-1-carboxylate (15d and 15d'): To a stirred solution of methyl 3-oxocyclobutanecarboxylate (340 µL, 3.15 mmol) in THF (6 mL) cooled to 0 °C, was added isopropenylmagnesium bromide solution (0.5 M in THF, 6 mL, 3.00 mmol) then the reaction mixture was warmed to rt and stirred for 18 h. The reaction was quenched using sat. aq. NH_4Cl solution (10 mL), then the aqueous phase was separated and extracted with diethyl ether (3 × 30 mL). The combined organic phases were concentrated *in vacuo*. Purification by flash column chromatography (SiO_2, cyclohexane:diethyl ether – 6:4) gave an inseparable mixture of the title compounds **15d** and **15d'** (151 mg, 887 µmol, 30%, d.r. 5:1) as a colourless oil; R_f = 0.11 (cyclohexane/diethyl ether; 6:4); υ_{max}/cm^{-1} 3441, 2971, 2935, 1700, 1240, 1182, 1107, 891; **Major diastereoisomer 15d**: ^{1}H NMR (400 MHz, $CDCl_3$) δ 5.01 (1H, s), 4.92 (1H, s), 3.71 (3H, s), 2.76 – 2.54 (3H, m), 2.48 (1H, br. s), 2.39 – 2.23 (2H, m), 1.81 (3H, s); ^{13}C{^{1}H} NMR (101 MHz, $CDCl_3$) δ 176.5, 146.7, 110.6, 74.3, 52.2, 38.5, 29.7, 17.6; **Minor diastereoisomer (Resolved signals) 15d'**: ^{1}H NMR (400 MHz, $CDCl_3$) δ 4.85 (1H, s), 3.68 (3H, s), 3.34 (1H, tt, J = 8.7, 4.1 Hz), 1.76 (3H, s); ^{13}C{^{1}H} NMR (101 MHz, $CDCl_3$) δ 175.9, 148.4, 110.5, 76.3, 51.9, 37.0, 31.2, 17.3; HRMS (ESI) m/z: $[M + Na]^+$ calcd for $C_9H_{14}NaO_3$, 193.0835; found, 193.0832.

Note: Attempts to establish the relative stereochemistry of the major and minor diastereoisomers proved unsuccessful. The major diastereoisomer is tentatively assigned as compound **15d** based on addition of the Grignard reagent to the least hindered face of the ketone in analogy with similar reactions reported in the literature.[62]

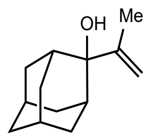

2-(Prop-1-en-2-yl)adamantan-2-ol (15e): Prepared according to general procedure D using isopropenylmagnesium bromide solution (0.5 M in THF, 15.0 mL, 7.50 mmol) and adamantanone (751 mg, 5.00 mmol) in THF (10 mL). Purification by flash column chromatography (SiO_2, pentane:diethyl ether – 8:2) gave the title compound **15e** (880 mg, 4.58 mmol, 92%) as a colourless crystalline solid; m.p. 49 – 51 °C (lit.[2] 50 – 51 °C); R_f = 0.53 (cyclohexane/diethyl ether; 6:4); ^{1}H NMR (400 MHz, $CDCl_3$) δ 5.07 (1H, s), 5.00 (1H, s), 2.33 – 2.23 (2H, m), 2.14 (2H, br. s), 1.86 – 1.80 (5H, m), 1.80 – 1.58 (9H, m), 1.20 (1H, br. s); $^{13}C\{^1H\}$ NMR (101 MHz, $CDCl_3$) δ 148.7, 112.1, 76.2, 37.8, 35.1, 34.7, 33.0, 27.3, 27.1, 18.7.

Data are consistent with those reported in the literature.[63]

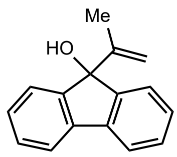

9-(Prop-1-en-2-yl)-9*H*-fluoren-9-ol (15f): Prepared according to general procedure D using isopropenylmagnesium bromide solution (0.5 M in THF, 9.00 mL, 4.50 mmol) and 9-fluorenone (542 mg, 3.00 mmol) in THF (6 mL). Purification by flash column chromatography (SiO_2, cyclohexane:diethyl ether – 9:1) gave the title compound **15f** (527 mg, 2.37 mmol, 79%) as a yellow oil; R_f = 0.22 (cyclohexane/ethyl acetate; 9:1); υ_{max}/cm^{-1} (thin film) 3531, 3405, 1447, 1255, 1055, 986, 901, 753, 730; ^{1}H NMR (400 MHz, $CDCl_3$) δ 7.64 (2H, ddd, *J* = 7.5, 1.0, 1.0 Hz), 7.45 – 7.35 (4H, m), 7.30 (2H, ddd, *J* = 7.5, 1.0, 1.0 Hz), 5.71 (1H, s), 5.13 (1H, s), 2.20 (1H, br. s), 1.24 (3H, s); $^{13}C\{^1H\}$ NMR (101 MHz, $CDCl_3$) δ 148.4, 145.5, 140.3, 129.3, 128.4, 123.9, 120.2, 111.0, 84.8, 18.7; HRMS (ESI) m/z: [M + Na]$^+$ calcd for $C_{16}H_{14}NaO$, 245.0937; found, 245.0936.

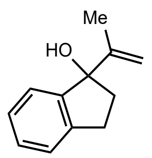

1-(Prop-1-en-2-yl)-2,3-dihydro-1*H*-inden-1-ol (15g): Prepared according to general procedure D using isopropenylmagnesium bromide solution (0.5 M in THF, 9.00 mL, 4.50 mmol) and 1-indanone (397 mg, 3.00 mmol) in THF (6 mL). Purification by flash column chromatography (SiO_2, cyclohexane:ethyl acetate – 8:2) gave the title compound **15g** (225 mg, 1.29 mmol, 43%) as a yellow oil; R_f = 0.44 (cyclohexane/ethyl acetate, 8:2); ^{1}H NMR (400 MHz, $CDCl_3$) δ 7.28 – 7.18 (4H, m), 5.06 (1H, s), 4.96 (1H, s), 3.10 (1H, ddd, *J* = 16.0, 8.3, 5.8 Hz), 2.88 (1H, ddd, *J* = 16.0, 8.4, 5.3 Hz), 2.48 (1H, ddd, *J* = 13.5, 8.4, 5.8 Hz), 2.13 (1H, ddd, *J* = 13.5, 8.4, 5.3 Hz), 1.82 (1H, br. s), 1.74 (3H, s);

$^{13}C\{^1H\}$ NMR (101 MHz, $CDCl_3$) δ 148.0, 146.4, 143.9, 128.4, 126.8, 125.0, 123.3, 110.8, 86.5, 39.9, 30.0, 19.5.

Data are consistent with those reported in the literature.[64]

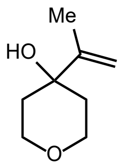

4-Isopropenyltetrahydropyran-4-ol (15h): Prepared according to general procedure D using isopropenylmagnesium bromide solution (0.5 M in THF, 9.00 mL, 4.50 mmol) and 4-oxotetrahydropyran (280 µL, 3.00 mmol) in THF (6 mL). Purification by flash column chromatography (SiO_2, cyclohexane:diethyl ether – 6:4) gave the title compound **15h** (400 mg, 2.83 mmol, 95%) as a colourless oil; R_f = 0.19 (cyclohexane/diethyl ether, 6:4); δ ^1H NMR (400 MHz, $CDCl_3$) δ 5.03 (1H, s), 4.87 (1H, s), 3.90 – 3.74 (4H, m), 2.00 – 1.90 (2H, m), 1.82 (3H, s), 1.53 – 1.45 (2H, m), 1.23 (1H, s); $^{13}C\{^1H\}$ NMR (101 MHz, $CDCl_3$) δ 151.0, 110.0, 71.3, 63.9, 36.2, 18.6.

Data are consistent with those reported in the literature.[63]

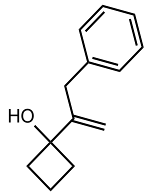

1-(3-Phenylprop-1-en-2-yl)cyclobutan-1-ol (15i): Prepared according to general procedure C using (2-bromoallyl)benzene[65] (900 mg, 4.57 mmol) in THF (4 mL), Mg (333 mg, 13.7 mmol) in THF (11 mL) and cyclobutanone (430 µL, 5.71 mmol) in THF (20 mL). Purification by flash column chromatography (SiO_2, cyclohexane:ethyl acetate – 9:1 → 6:4) gave the title compound **15i** (662 mg, 3.52 mmol, 77%) as a yellow oil; R_f = 0.50 (cyclohexane/ethyl acetate, 6:4); ^1H NMR (400 MHz, $CDCl_3$) δ 7.35 – 7.25 (2H, m), 7.25 – 7.16 (3H, m), 5.16 (1H, d, J = 1.0 Hz), 4.70 (1H, d, J = 1.0 Hz), 3.46 (2H, s), 2.40 – 2.29 (2H, m), 2.12 – 2.00 (2H, m), 1.99 – 1.85 (1H, m), 1.67 – 1.50 (1H, m); $^{13}C\{^1H\}$ NMR (101 MHz, $CDCl_3$) 152.0, 140.2, 129.5, 128.5, 126.2, 111.2, 78.7, 37.5, 35.0, 13.3.

Data are consistent with those reported in the literature.[12]

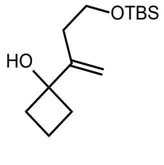

1-{4-[(*tert*-Butyldimethylsilyl)oxy]but-1-en-2-yl}cyclobutan-1-ol (15j): To a stirred solution of [(3-bromobut-3-en-1-yl)oxy](*tert*-butyl)dimethylsilane[66] (956 mg, 3.60 mmol) in THF (6 mL) at –78 °C,

was added *n*-BuLi (2.5 M in hexanes, 1.44 mL, 3.60 mmol) then the reaction mixture was stirred at this temperature for 2 h. Cyclobutanone (230 µL, 3.00 mmol) was added, then the reaction mixture was warmed to rt and stirred for 18 h. The reaction mixture was quenched using sat. aq. NH_4Cl solution (10 mL) then the aqueous layer was separated and extracted using DCM (3 × 30 mL). The combined organic phases were dried ($MgSO_4$), filtered and concentrated *in vacuo*. Purification by flash column chromatography (SiO_2, cyclohexane:diethyl ether – 20:1 → 9:1) gave the title compound **15j** (196 mg, 764 µmol, 25%) as a colourless oil; R_f = 0.10 (cyclohexane/diethyl ether; 20:1); υ_{max}/cm^{-1} (thin film) 3428, 2952, 2930, 1249, 1088, 832, 774; ^{1}H NMR (400 MHz, $CDCl_3$) δ 5.16 (1H, d, *J* = 1.2 Hz), 4.94 (1H, d, *J* = 1.2 Hz), 4.22 (1H, s), 3.77 (2H, t, *J* = 5.8 Hz), 2.35 (2H, t, *J* = 5.8 Hz), 2.30 – 2.19 (2H, m), 2.19 – 2.07 (2H, m), 1.84 – 1.75 (1H, m), 1.56 – 1.41 (1H, m), 0.89 (9H, s), 0.07 (6H, s); ^{13}C{^{1}H} NMR (101 MHz, $CDCl_3$) δ 150.5, 110.9, 77.4, 65.0, 35.3, 34.7, 26.1, 18.5, 12.8, −5.4; HRMS (ESI) m/z: [M + Na]$^+$ calcd for $C_{14}H_{28}NaO_2Si$, 279.1745; found, 279.1751.

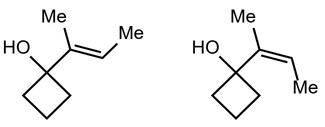

15k 15k'

(*E*)-1-(But-2-en-2-yl)cyclobutan-1-ol (15k) and **(*Z*)-1-(But-2-en-2-yl)cyclobutan-1-ol (15k')**: Prepared according to general procedure D using 1-methyl-1-propenylmagnesium bromide solution (0.5 M in THF, 9.00 mL, 4.50 mmol) and cyclobutanone (220 µL, 3.00 mmol) in THF (6 mL). Purification by flash column chromatography (SiO_2, cyclohexane:ethyl acetate – 8:2) gave an inseperable mixture of the title compounds **15k** and **15k'** (124 mg, 982 µmol, 33%, d.r. 2:1) as a colourless oil; R_f = 0.23 (cyclohexane/ethyl acetate; 9:1); ^{1}H NMR (400 MHz, $CDCl_3$) δ 5.55 (1H, qd, *J* = 6.5, 1.2 Hz), 5.30 (1H, qd, *J* = 7.2, 1.5 Hz), 2.47 – 2.34 (2H, m), 2.33 – 2.26 (2H, m), 2.20 – 2.07 (3H, m), 2.08 – 1.96 (2H, m), 1.92 – 1.80 (1H, m), 1.75 – 1.58 (13H, m), 1.56 – 1.46 (1H, m); ^{13}C{^{1}H} NMR (101 MHz, $CDCl_3$) δ 140.2, 138.5, 122.8, 118.3, 79.3, 78.4, 36.4, 34.2, 20.4, 15.2, 14.6, 13.4, 13.2, 11.2.

Data are consistent with those reported in the literature.[67]

4. Photoredox Mediated Amidation / Semipinacol Rearrangement

General procedure E - Photoredox mediated amidation/semipinacol rearrangement:

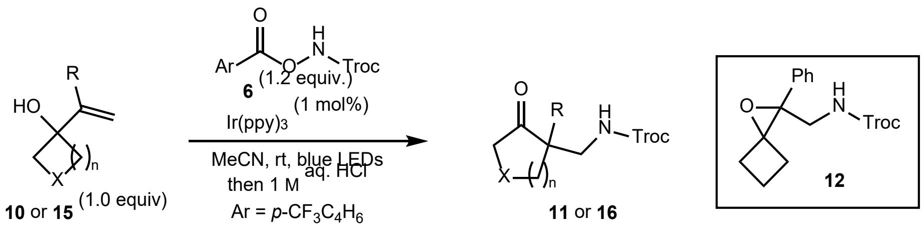

To a mixture of olefin substrate **10** or **15** (1.00 equiv.), amidyl radical precursor **6** (1.20 equiv.) and Ir(ppy)$_3$ (1 mol%), was added acetonitrile (0.2 M). The reaction mixture was sparged with argon for 10 minutes, then stirred at rt whilst irradiating with blue LEDs (450 nm). After 3 h, 1 M aq. HCl solution (4 mL) was added, then the reaction was stirred at rt for a further 18 h. The aqueous layer was separated and extracted with ethyl acetate (3 × 20 mL), then the combined organic phases were washed with 1 M aq. NaOH solution (30 mL) and brine (30 mL). The combined organic phases were dried ($MgSO_4$), filtered and concentrated *in vacuo*.

Data for Novel Products Prepared During Optimisation Studies:

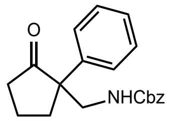

Benzyl [(2-oxo-1-phenylcyclopentyl)methyl]carbamate (S6): Prepared according to general procedure E using 1-(1-phenylvinyl)cyclobutan-1-ol **10a** (34.8 mg, 200 µmol), benzyl {[4-(trifluoromethyl)benzoyl]oxy}carbamate **S1** (81.4 mg, 240 µmol), Ir(ppy)$_3$ (1.3 mg, 2.00 µmol, 1 mol%) and acetonitrile (1 mL). Purification by flash column chromatography (SiO_2, cyclohexane:ethyl acetate – 19:1) gave the title compound **S6** (28 mg, 87 µmol, 43%) as a colourless oil; R_f = 0.27 (cyclohexane/ethyl acetate; 4:1); υ_{max}/cm^{-1} (thin film) 3338, 2959, 1721, 1513, 1497, 1233, 1153, 1136, 754, 699; ^{1}H NMR (500 MHz, $CDCl_3$) δ 7.41 – 7.23 (10H, m), 5.04 (2H, s), 5.02 – 4.94 (1H, m), 3.59 (1H, dd, *J* = 14.0, 6.8 Hz), 3.51 (1H, dd, *J* = 14.0, 6.1 Hz), 2.56 – 2.48 (1H, m), 2.36 (1H, dddd, *J* = 19.3, 9.3, 4.1, 1.7 Hz), 2.26 (1H, dt, *J* = 19.4, 8.7 Hz), 2.15 (1H, ddd, *J* = 13.4, 10.5, 6.8 Hz), 2.01 – 1.87 (1H, m), 1.84 – 1.69 (1H, m); ^{13}C{^{1}H} NMR (126 MHz, $CDCl_3$) δ 219.8, 156.8, 137.9, 136.6, 129.1, 128.6, 128.3, 128.2, 127.6, 126.9, 66.9, 58.6, 47.2, 37.9, 32.7, 18.6; HRMS (ESI) m/z: [M + Na]$^+$ calcd for $C_{20}H_{21}NNaO_3$, 346.1414; found, 346.1413.

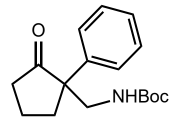

***tert*-Butyl [(2-oxo-1-phenylcyclopentyl)methyl]carbamate (S7):** Prepared according to general procedure E using 1-(1-phenylvinyl)cyclobutan-1-ol **10a** (34.8 mg, 200 μmol), tert-butyl {[4-(trifluoromethyl)benzoyl]oxy}carbamate **S2** (73.3 mg, 240 μmol), Ir(ppy)$_3$ (1.3 mg, 2.00 μmol, 1 mol%) and acetonitrile (1 mL). Purification by flash column chromatography (SiO_2, cyclohexane:ethyl acetate – 23:2) gave the title compound **S7** (31.9 mg, 110 μmol, 55%) as a colourless oil; R_f = 0.42 (cyclohexane/ethyl acetate; 7:3); υ_{max}/cm^{-1} (thin film) 3366, 2973, 1709, 1599, 1497, 1453, 1391, 1243, 1158, 755, 699; ^{1}H NMR (500 MHz, $CDCl_3$) δ 7.34 (4H, d, *J* = 1.0 Hz), 7.26 (1H, q, *J* = 4.6 Hz), 4.73 (1H, t, *J* = 6.4 Hz), 3.54 – 3.40 (2H, m), 2.54 – 2.46 (1H, m), 2.40 – 2.21 (2H, m), 2.15 (1H, ddd, *J* = 13.5, 10.3, 6.8 Hz), 2.01 – 1.90 (1H, m), 1.78 (1H, dddd, *J* = 15.6, 9.4, 7.7, 4.5 Hz), 1.37 (9H, s); ^{13}C{^{1}H} NMR (126 MHz, $CDCl_3$) δ 220.0, 156.2, 138.2, 129.0, 127.5, 126.9, 79.4, 58.8, 46.6, 38.0, 32.8, 28.4, 18.6; HRMS (ESI) m/z: $[M + H]^+$ calcd for $C_{17}H_{24}NO_3$, 290.1751; found, 290.1752.

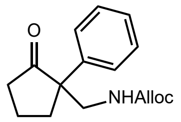

Allyl [(2-oxo-1-phenylcyclopentyl)methyl]carbamate (S8): Prepared according to general procedure E using 1-(1-phenylvinyl)cyclobutan-1-ol **10a** (34.8 mg, 200 μmol), allyl {[4-(trifluoromethyl)benzoyl]oxy}carbamate **S3** (69.4 mg, 240 μmol), Ir(ppy)$_3$ (1.3 mg, 2.00 μmol, 1 mol%) and acetonitrile (1 mL). Purification by flash column chromatography (SiO_2, cyclohexane:ethyl acetate – 9:1) gave the title compound **S8** (20 mg, 73 μmol, 31%) as a colourless oil; R_f = 0.24 (cyclohexane/ethyl acetate; 4:1); υ_{max}/cm^{-1} (thin film) 3347, 2961, 1722, 1514, 1497, 1234, 1154, 994, 759, 701; ^{1}H NMR (500 MHz, $CDCl_3$) δ 7.37 – 7.32 (4H, m), 7.31 – 7.26 (1H, m), 5.91 – 5.80 (1H, m), 5.24 (1H, dq, *J* = 17.2, 1.7 Hz), 5.18 (1H, dd, *J* = 10.7, 1.7 Hz), 4.93 (1H, t, *J* = 6.5 Hz), 4.50 (2H, dt, *J* = 5.7, 1.5 Hz), 3.58 (1H, dd, *J* = 14.0, 6.7 Hz), 3.50 (1H, dd, *J* = 14.0, 6.2 Hz), 2.56 – 2.48 (1H, m), 2.36 (1H, dddd, *J* = 19.4, 9.3, 4.1, 1.7 Hz), 2.27 (1H, dt, *J* = 19.4, 8.7 Hz), 2.16 (1H, ddd, *J* = 13.4, 10.5, 6.8 Hz), 2.01 – 1.91 (1H, m), 1.78 (1H, ddtd, *J* = 13.0, 10.5, 9.1, 6.4 Hz); ^{13}C{^{1}H} NMR (126 MHz, $CDCl_3$) δ 219.8, 156.6, 137.9, 132.9, 129.1, 127.6, 126.9, 117.8, 65.7, 58.6, 47.1, 37.9, 32.7, 18.6; HRMS (ESI) m/z: $[M + NH_4]^+$ calcd for $C_{16}H_{23}N_2O_3$, 291.1703; found 291.1704.

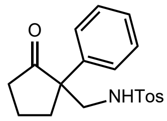

4-Methyl-*N*-[(2-oxo-1-phenylcyclopentyl)methyl]benzenesulfonamide (S9): Prepared according to general procedure E using 1-(1-phenylvinyl)cyclobutan-1-ol **10a** (69.7 mg, 400 μmol), 1-[(4-methylphenyl)sulfonamido]pyridin-1-ium tetrafluoroborate **S5** (161 mg, 480 μmol), $Ir(ppy)_3$ (2.7 mg, 4.00 μmol, 1 mol%) and acetonitrile (2 mL). Purification by flash column chromatography (SiO_2, cyclohexane:ethyl acetate – 9:1) gave the title compound **S9** (23.8 mg, 69 μmol, 17%) as a colourless oil; R_f = 0.3 (cyclohexane/ethyl acetate; 4:1); υ_{max}/cm^{-1} (thin film) 3280, 2923, 1731, 1446, 1331, 1160, 1092, 759, 701, 663; ^{1}H NMR (400 MHz, $CDCl_3$) δ 7.61 (2H, d, *J* = 8.0 Hz), 7.28 (7H, td, *J* = 12.7, 7.3 Hz), 4.67 (1H, t, *J* = 6.9 Hz), 3.19 (2H, qd, *J* = 13.0, 6.9 Hz), 2.56 (1H, ddt, *J* = 13.5, 6.6, 2.2 Hz), 2.45 – 2.17 (6H, m), 1.97 (1H, dddt, *J* = 11.9, 9.0, 6.5, 3.0 Hz), 1.85 – 1.69 (1H, m); $^{13}C\{^1H\}$ NMR (101 MHz, $CDCl_3$) δ 219.4, 143.5, 137.0, 136.9, 129.9, 129.2, 127.9, 127.0, 126.8, 57.9, 49.5, 37.6, 32.0, 21.6, 18.5; HRMS (ESI) m/z: $[M + H]^+$ calcd for $C_{19}H_{22}NO_3S$, 344.1315; found, 344.1317.

Procedures and Characterisation Data for Products:

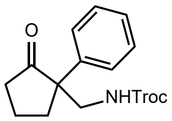

2,2,2-Trichloroethyl [(2-oxo-1-phenylcyclopentyl)methyl]carbamate (11a): Prepared according to general procedure E using 1-(1-phenylvinyl)cyclobutanol **10a** (71.0 mg, 400 μmol), 2,2,2-trichloroethyl {[4-(trifluoromethyl)benzoyl]oxy}carbamate **6** (183 mg, 480 μmol), $[Ir(ppy)_3]$ (2.7 mg, 4.00 μmol, 1 mol%) and acetonitrile (2 mL). Purification by flash column chromatography (SiO_2, cyclohexane:ethyl acetate – 19:1) gave the title compound **11a** (99.9 mg, 274 μmol, 68%) as a colourless oil; R_f = 0.55 (cyclohexane/ethyl acetate; 1:1); υ_{max}/cm^{-1} (thin film) 3331, 2959, 1724, 1513, 1229, 1139, 803, 722, 699, 566; ^{1}H NMR (400 MHz, $CDCl_3$) δ 7.42 – 7.27 (5H, m), 5.33 – 5.25 (1H, m), 4.68 (1H, d, *J* = 12.1 Hz), 4.67 (1H, d, *J* = 12.1 Hz), 3.65 (1H, dd, *J*= 14.1, 6.9 Hz), 3.52 (1H, dd, *J* = 14.0, 7.0 Hz), 2.60 – 2.50 (1H, m), 2.44 – 2.22 (2H, m), 2.18 – 2.08 (1H, m), 2.00 – 1.91 (1H, m), 1.84 – 1.72 (1H, m); $^{13}C\{^1H\}$ NMR (101MHz, $CDCl_3$) δ 219.9, 155.0, 137.6, 129.2, 127.8, 126.9, 95.7, 74.6, 58.5, 47.4, 37.8, 32.9, 18.6; HRMS (ESI) m/z: $[M + Na]^+$ calcd for $C_{15}H_{16}{}^{35}Cl_3NNaO_3$, 386.0088; found, 386.0087.

Note: Whilst we were unable to isolate a pure sample of the epoxide side-product **12** observed during optimization studies, a sample of sufficient purity for characterization by $^1H/^{13}C$ NMR spectroscopy was obtained by flash column chromatography (SiO_2, cyclohexane:ethyl acetate – 4:1). The shifts for key ^{13}C signals (epoxide quaternary carbons) are consistent with a structurally related epoxide previously reported by Glorius and co-workers.[13]

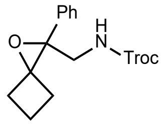

2,2,2-Trichloroethyl ((2-phenyl-1-oxaspiro[2.3]hexan-2-yl)methyl)carbamate (12): ^{1}H NMR (500 MHz, $CDCl_3$) 7.38 – 7.32 (2H, m), 7.31 – 7.27 (3H, m), 5.12 – 5.04 (1H, m), 4.68 (1H, d, J = 12.0 Hz), 4.66 (1H, d, J = 12.0 Hz), 4.18 (1H, dd, J = 14.4, 6.4 Hz), 3.47 (1H, d, J = 14.4, 5.2 Hz), 2.61 – 2.45 (2H, m), 2.35 – 2.26 (1H, m), 1.95 – 1.79 (2H, m), 1.71 (1H, ddd, J = 17.1, 10.8, 8.4 Hz); ^{13}C{^{1}H} NMR (126 MHz, $CDCl_3$) δ 154.6, 136.0, 128.5, 128.0, 126.5, 95.6, 74.7, 70.2, 65.4, 44.1, 29.5, 29.3, 13.0.

Scale up reaction (1.44 mmol):

Prepared according to general procedure E using 1-(1-phenylvinyl)cyclobutanol **10a** (250 mg, 1.44 mmol), 2,2,2-trichloroethyl {[4-(trifluoromethyl)benzoyl]oxy}carbamate **6** (655.3 mg, 1.72 mmol), [Ir(ppy)$_3$] (9.4 mg,14.0 μmol) and acetonitrile (7.2 mL). Purification by flash column chromatography (SiO_2, cyclohexane:ethyl acetate 19:1) gave the title compound **11a** (328 mg, 0.902 mmol, 63%) as a colourless oil.

Data for the product are consistent with those reported above.

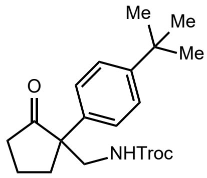

2,2,2-Trichloroethyl ((1-(4-(*tert*-butyl)phenyl)-2-oxocyclopentyl)methyl)carbamate (11b): Prepared according to general procedure E using 1-{1-[4-(*tert*-butyl)phenyl]vinyl}cyclobutan-1-ol **10b** (92.0 mg, 400 μmol), {[4-(trifluoromethyl)benzoyl]oxy}carbamate **6** (183 mg, 480 μmol), [Ir(ppy)$_3$] (2.7 mg, 4.00 μmol, 1 mol%) and acetonitrile (2 mL). Purification by flash column chromatography (SiO_2, cyclohexane:ethyl acetate – 20:1) gave the title compound **11b** (144 mg, 342 μmol, 86%) as a colourless oil; R_f = 0.31 (cyclohexane/ethyl acetate; 8:2); υ_{max}/cm^{-1} (thin film) 3342, 2961, 1726, 1509, 1229, 1140, 815, 721, 566; ^{1}H NMR (400 MHz, $CDCl_3$) δ 7.36 (2H, d, J = 8.6 Hz), 7.25 (2H, d, J = 8.6 Hz), 5.25 (1H, t, J = 6.9 Hz), 4.72 – 4.60 (2H, m), 3.63 (1H, dd, J = 14.0, 6.9 Hz), 3.51 (1H, dd, J = 14.0, 6.9 Hz), 2.61 – 2.47 (1H, m), 2.44 – 2.32 (1H, m), 2.31 – 2.18 (1H, m), 2.16 – 2.05 (1H, m), 2.01 – 1.89 (1H, m), 1.85 – 1.72 (1H, m), 1.30 (9H, s); ^{13}C{^{1}H} NMR (101 MHz, $CDCl_3$) δ 220.0, 155.0, 150.6, 134.3, 126.6, 126.0, 95.7, 74.6, 58.1, 47.3, 37.8, 34.6, 32.8, 31.4, 18.6; HRMS (ESI) m/z: $[M + NH_4]^+$calcd for $C_{19}H_{28}{}^{35}Cl_3N_2O_3$, 437.1161; found, 437.1160.

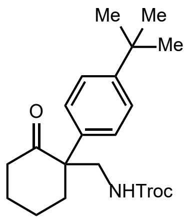

2,2,2-Trichloroethyl ((1-(4-(*tert*-butyl)phenyl)-2-oxocyclohexyl)methyl)carbamate (11c): Prepared according to general procedure E using 1-{1-[4-(*tert*-butyl)phenyl]vinyl}cyclopentan-1-ol **10c** (98.0 mg, 400 μmol), {[4-(trifluoromethyl)benzoyl]oxy}carbamate **6** (183 mg, 480 μmol), [Ir(ppy)$_3$] (2.7 mg, 4.00 μmol, 1 mol%) and acetonitrile (2 mL). Purification by flash column chromatography (SiO_2, cyclohexane:diethyl ether – 20:1) gave the title compound **11c** (118 mg, 271 μmol, 68%) as a colourless oil; R_f = 0.10 (cyclohexane/diethyl ether; 20:1); υ_{max}/cm^{-1} (thin film) 3356, 2948, 2867, 1740, 1701, 1506, 1212, 1142, 1127, 810, 727, 568; ^{1}H NMR (400 MHz, $CDCl_3$) δ 7.37 (2H, d, J = 8.5 Hz), 7.09 (2H, d, J = 8.4 Hz), 5.47 (1H, t, J = 6.6 Hz), 4.62 (2H, s), 3.45 (1H, dd, J = 13.9, 6.1 Hz), 3.33 (1H, dd, J = 13.9, 6.1 Hz), 2.68 – 2.62 (1H, m), 2.48 – 2.36 (1H, m), 2.33 – 2.27 (1H, m), 2.01 – 1.82 (2H, m), 1.78 – 1.61 (3H, m), 1.31 (9H, s); ^{13}C{^{1}H} NMR (101 MHz, $CDCl_3$) δ 214.8, 155.0, 150.6, 135.2, 126.7, 126.2, 95.8, 74.5, 59.0, 49.6, 40.3, 34.6, 32.9, 31.4, 28.3, 21.2; HRMS (ESI) m/z: [M + H]$^+$ calcd for $C_{20}H_{27}{}^{35}Cl_3NO_3$, 434.1051; found, 434.1050.

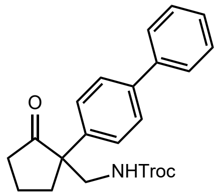

2,2,2-Trichloroethyl ((1-([1,1'-biphenyl]-4-yl)-2-oxocyclopentyl)methyl)carbamate (11d): Prepared according to general procedure E using 1-{1-[(1,1'-biphenyl)-4-yl]vinyl}cyclobutan-1-ol **10d** (100 mg, 400 μmol), {[4-(trifluoromethyl)benzoyl]oxy}carbamate **6** (183 mg, 480 μmol), [Ir(ppy)$_3$] (2.7 mg, 4.00 μmol, 1 mol%) and acetonitrile (2 mL). Purification by flash column chromatography (SiO_2, cyclohexane:diethyl ether – 9:1) gave the title compound **11d** (169 mg, 383 μmol, 96%) as a colourless oil that crystallised on standing; m.p. 86 – 88 °C; R_f = 0.43 (cyclohexane/ethyl acetate; 6:4); υ_{max}/cm^{-1} (thin film) 3336, 2953, 1724, 1512, 1229, 1142, 765, 726, 698; ^{1}H NMR (400 MHz, $CDCl_3$) δ 7.64 – 7.57 (4H, m), 7.49 – 7.42 (4H, m), 7.41 – 7.34 (1H, m), 5.36 (1H, t, *J* = 6.1 Hz), 4.78 – 4.65 (2H, m), 3.72 (1H, dd, *J* = 14.1, 7.1 Hz), 3.59 (1H, dd, *J* = 14.1, 6.0 Hz), 2.62 – 2.52 (1H, m), 2.50 – 2.39 (1H, m), 2.36 – 2.24 (1H, m), 2.22 – 2.12 (1H, m), 2.05 – 1.94 (1H, m), 1.88 – 1.79 (1H, m); ^{13}C{^{1}H} NMR (101 MHz, $CDCl_3$) δ 219.8, 155.0, 140.6, 140.4, 136.5, 128.9, 127.7, 127.6, 127.4, 127.1, 95.7, 74.5, 58.3, 47.3, 37.8, 32.8, 18.6; HRMS (ESI) m/z: [M + H]$^+$ calcd for $C_{21}H_{21}{}^{35}Cl_3NO_3$, 440.0582; found, 440.0582.

Slow diffusion (Layering technique) of heptane into a solution of ketone **11d** in DCM gave crystals that were suitable for X-ray crystallography:

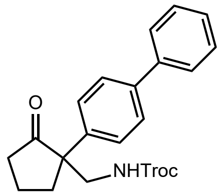

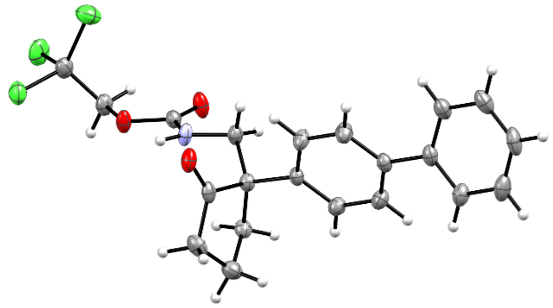

X-Ray crystal structure of compound 11d (CCDC No. 2237285) depicted using Mercury 2022.3.0[68]

The second molecule within the asymmetric unit has been omitted for clarity – See section 6 for further details.

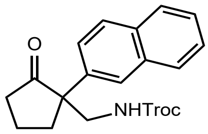

2,2,2-Trichloroethyl {[1-(naphthalen-2-yl)-2-oxocyclopentyl]methyl}carbamate (11e): Prepared according to general procedure E using 1-(1-(naphthalen-2-yl)vinyl)cyclobutan-1-ol **10e** (90.0 mg, 400 µmol), {[4-(trifluoromethyl)benzoyl]oxy}carbamate **6** (183 mg, 480 µmol), [Ir(ppy)$_3$] (2.7 mg, 4.00 µmol, 1 mol%) and acetonitrile (2 mL). Purification by flash column chromatography (SiO_2, cyclohexane:diethyl ether – 15:1) gave the title compound **11e** (109 mg, 263 µmol, 66%) as a colourless oil; R_f = 0.42 (cyclohexane/ethyl acetate; 8:2); υ_{max}/cm^{-1} (thin film) 3334, 2962, 1725, 1508, 1230, 1140, 815, 723; ^{1}H NMR (400 MHz, $CDCl_3$) δ 7.86 (1H, d, *J* = 8.7 Hz), 7.84 – 7.78 (2H, m), 7.73 – 7.69 (1H, m), 7.54 – 7.46 (m, 3H), 5.31 (1H, t, *J* = 6.1 Hz), 4.64 – 4.58 (2H, m), 3.75 (1H, dd, *J* = 14.1, 5.8 Hz), 3.60 (1H, dd, *J* = 14.1, 6.4 Hz), 2.73 – 2.61 (1H, m), 2.52 – 2.38 (1H, m), 2.38 – 2.26 (1H, m), 2.26 – 2.16 (1H, m), 2.07 – 1.94 (1H, m), 1.88 – 1.71 (1H, m); ^{13}C{^{1}H} NMR (101 MHz, $CDCl_3$) δ 220.0, 155.0, 134.8, 133.3, 132.7, 129.2, 128.2, 127.7, 126.6, 126.6, 126.4, 124.4, 95.6, 74.3, 58.7, 47.3, 37.9, 33.0, 18.7; HRMS (ESI) m/z: [M + Na]$^+$ calcd for $C_{19}H_{18}{}^{35}Cl_3NNaO_3$, 436.0244; found, 436.0241.

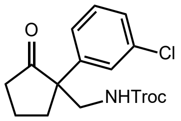

2,2,2-Trichloroethyl {[1-(3-chlorophenyl)-2-oxocyclopentyl]methyl}carbamate (11f): Prepared according to general procedure E using 1-[1-(3-chlorophenyl)vinyl]cyclobutan-1-ol **10f** (84.0 mg, 400 µmol), {[4-(trifluoromethyl)benzoyl]oxy}carbamate **6** (183 mg, 480 µmol), [Ir(ppy)$_3$] (2.70 mg, 4.00

μmol, 1 mol%) and acetonitrile (2 mL). Purification by flash column chromatography (SiO_2, cyclohexane:diethyl ether – 9:1 → 6:4) gave the title compound **11f** (78.0 mg, 195 μmol, 49%) as a colourless oil; R_f = 0.44 (cyclohexane/ethyl acetate; 6:4); υ_{max}/cm^{-1} (thin film) 3337, 2954, 1724, 1514, 1230, 1142, 723, 696; ^{1}H NMR (400 MHz, $CDCl_3$) δ 7.35 – 7.21 (4H, m), 5.28 (1H, t, *J* = 6.2 Hz), 4.77 – 4.61 (2H, m), 3.62 (1H, dd, J = 14.1, 7.0 Hz), 3.49 (1H, dd, J = 14.1, 6.0 Hz), 2.54 – 2.44 (1H, m), 2.45 – 2.22 (2H, m), 2.20 – 2.09 (1H, m), 2.04 – 1.92 (1H, m), 1.85 – 1.71 (1H, m); ^{13}C{^{1}H} NMR (101 MHz, $CDCl_3$) δ 219.1, 155.0, 139.8, 135.1, 130.3, 128.0, 127.3, 125.2, 95.6, 74.6, 58.2, 47.3, 37.9, 32.9, 18.6; HRMS (ESI) m/z: [M + NH_4]$^+$ calcd for $C_{15}H_{19}{}^{35}Cl_4N_2O_3$, 415.0144; found, 415.0142.

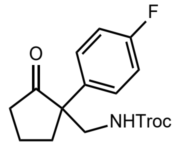

2,2,2-Trichloroethyl {[1-(4-fluorophenyl)-2-oxocyclopentyl]methyl}carbamate (11g): Prepared according to general procedure E using 1-[1-(4-fluorophenyl)vinyl]cyclobutan-1-ol **10g** (77.0 mg, 400 μmol), {[4-(trifluoromethyl)benzoyl]oxy}carbamate **6** (183 mg, 480 μmol), [Ir(ppy)$_3$] (2.7 mg, 4.00 μmol, 1 mol%) and acetonitrile (2 mL). Purification by flash column chromatography (SiO_2, cyclohexane:diethyl ether – 20:1 → 9:1) gave the title compound **11g** (123 mg, 321 μmol, 80%) as a colourless oil; R_f = 0.34 (cyclohexane/ethyl acetate; 6:4); υ_{max}/cm^{-1} (thin film) 3326, 2961, 1724, 1507, 1226, 1141, 815, 721; ^{1}H NMR (400 MHz, $CDCl_3$) δ 7.37 – 7.29 (2H, m), 7.08 – 7.00 (2H, m), 5.26 (1H, t, *J* = 6.3 Hz), 4.69 – 4.60 (2H, m), 3.62 (1H, dd, J = 14.1, 7.1 Hz), 3.48 (1H, dd, J = 14.1, 6.0 Hz), 2.56 – 2.45 (1H, m), 2.43 – 2.23 (2H, m), 2.20 – 2.08 (1H, m), 2.02 – 1.92 (1H, m), 1.83 – 1.69 (1H, m); ^{13}C{^{1}H} NMR (101 MHz, $CDCl_3$) δ 219.6, 162.3 (d, J = 243.0 Hz), 155.0, 133.2 (d, J = 3.2 Hz), 128.7 (d, J = 8.0 Hz), 116.0 (d, J = 21.4 Hz) 95.7, 74.6, 57.8, 47.4, 37.8, 33.0, 18.5; ^{19}F NMR (376 MHz, $CDCl_3$) δ −114.6 ppm; HRMS ((ESI) m/z: [M + H]$^+$ calcd for $C_{15}H_{16}{}^{35}Cl_3{}^{19}FNO_3$, 382.0174; found, 382.0174.

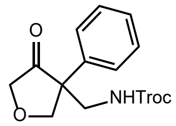

2,2,2-Trichloroethyl [(4-oxo-3-phenyltetrahydrofuran-3-yl)methyl]carbamate (11h): Prepared according to general procedure E using 3-(1-phenylvinyl)oxetan-3-ol **10h** (70.5 mg, 400 μmol), benzyl {[4-(trifluoromethyl)benzoyl]oxy}carbamate **6** (182.6 mg, 480 μmol), Ir(ppy)$_3$ (2.7 mg, 4.00 μmol, 1 mol%) and acetonitrile (2 mL). Purification by flash column chromatography (SiO_2, cyclohexane:ethyl acetate – 9:1) gave the title compound **11h** (58 mg, 158 μmol, 40%) as a colourless oil; R_f = 0.20 (cyclohexane/ethyl acetate; 4:1); υ_{max}/cm^{-1} (thin film) 3331, 2952, 1735, 1532, 1449, 1235, 1151, 1031, 812, 722, 699; ^{1}H NMR (400 MHz, $CDCl_3$) δ 7.44 – 7.28 (5H, m), 5.20 (1H, t, *J* = 6.6 Hz), 4.74 – 4.61 (3H, m), 4.36 (1H, d, *J* = 10.2 Hz), 4.08 (2H, app d, *J* = 17.3 Hz), 3.83 – 3.67 (2H, m); ^{13}C{^{1}H} NMR

(101 MHz, $CDCl_3$) δ 214.4, 155.0, 135.7, 129.4, 128.3, 126.8, 95.5, 75.1, 74.7, 71.5, 56.7, 45.5; HRMS (ESI) m/z: $[M + Na]^+$ calcd for $C_{14}H_{14}{}^{35}Cl_3NNaO_4$, 387.9881; found, 387.9884.

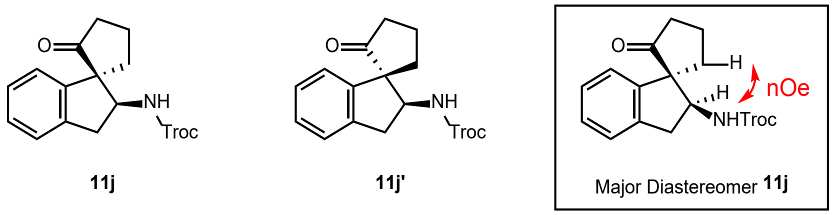

2,2,2-Trichloroethyl {(1*R,2'*S**)-2-oxo-2',3'-dihydrospiro[cyclopentane-1,1'-inden]-2'-yl}carbamate (11j) and 2,2,2-Trichloroethyl {(1*S**,2'*S**)-2-oxo-2',3'-dihydrospiro[cyclopentane-1,1'-inden]-2'-yl}carbamate (11j'):** Prepared according to general procedure E using 1-(1*H*-inden-3-yl)cyclobutan-1-ol **10j** (75.0 mg, 400 µmol), 2,2,2-trichloroethyl {[4-(trifluoromethyl)benzoyl]oxy}carbamate **6** (183 mg, 480 µmol), [Ir(ppy)$_3$] (2.7 mg, 4.00 µmol, 1 mol%) and acetonitrile (2 mL). Purification by flash column chromatography (SiO_2, cyclohexane:diethyl ether – 8:2 → 6:4) gave the title compound **11j** (97.0 mg, 258 µmol, 64%) as a colourless oil and the minor diastereoisomer **11j'** (30 mg, 80.0 µmol, 20%) as a colourless oil.

2,2,2-Trichloroethyl {(1*R,2'*S**)-2-oxo-2',3'-dihydrospiro[cyclopentane-1,1'-inden]-2'-yl}carbamate (11j):** R_f = 0.43 (cyclohexane/diethyl ether; 6:4); υ_{max}/cm^{-1} (thin film) 3319, 2954, 1718, 1510, 1238, 724; ^1H NMR (400 MHz, $CDCl_3$) δ 7.25 – 7.18 (3H, m), 7.14 – 7.07 (1H, m), 5.51 (1H, d, J = 9.9 Hz), 4.75 (1H, d, J = 12.0 Hz), 4.69 (1H, d, J = 12.0 Hz), 4.62 (1H, dt, J = 9.9, 7.8 Hz), 3.27 (1H, dd, J = 15.6, 7.8 Hz), 3.15 (1H, dd, J = 15.6, 7.8 Hz), 2.51 – 2.27 (4H, m), 2.24 – 2.09 (2H, m); $^{13}C\{^1H\}$ NMR (101 MHz, $CDCl_3$) δ 219.7, 154.6, 144.0, 141.0, 128.1, 127.6, 125.2, 123.2, 95.6, 74.7, 64.5, 60.1, 38.7, 38.2, 35.6, 20.2; (ESI) m/z: $[M + Na]^+$ calcd for $C_{16}H_{16}{}^{35}Cl_3NNaO_3$, 398.0088; found, 398.0089.

2,2,2-Trichloroethyl {(1*S*,2'*S*)-2-oxo-2',3'-dihydrospiro[cyclopentane-1,1'-inden]-2'-yl}carbamate (11j'): R_f = 0.40 (cyclohexane/diethyl ether; 6:4); υ_{max}/cm^{-1} (thin film) 3332, 2958, 1729, 1531, 1256, 1238, 738; ^1H NMR (400 MHz, $CDCl_3$) δ 7.34 – 7.19 (4H, m), 7.12 – 7.03 (1H, m), 5.19 (1H, d, J = 8.5 Hz), 4.86 (1H, d, J = 12.0 Hz), 4.70 – 4.59 (2H, m), 3.62 (1H, dd, J = 16.0, 7.0 Hz), 2.82 (1H, dd, J = 16.0, 4.0 Hz), 2.59 – 2.34 (3H, m), 2.26 – 2.05 (3H, m); $^{13}C\{^1H\}$ NMR (101 MHz, $CDCl_3$) δ 218.1, 154.4, 143.9, 140.9, 128.2, 127.6, 125.3, 123.4, 95.7, 74.7, 65.3, 58.1, 38.7, 37.7, 30.9, 19.7; HRMS (ESI) m/z: $[M + Na]^+$ calcd for $C_{16}H_{16}{}^{35}Cl_3NNaO_3$, 398.0088; found, 398.0088.

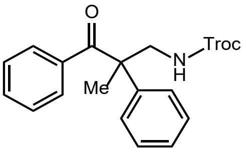

2,2,2-Trichloroethyl (2-methyl-3-oxo-2,3-diphenylpropyl)carbamate (14): Prepared according to general procedure E using 2-methyl-1,1-diphenylprop-2-en-1-ol **13** (90.0 mg, 400 µmol), 2,2,2-trichloroethyl {[4-(trifluoromethyl)benzoyl]oxy}carbamate **6** (183 mg, 480 µmol), [Ir(ppy)$_3$] (2.7 mg, 4.00 µmol, 1 mol%) and acetonitrile (2 mL). Purification by flash column chromatography (SiO_2, cyclohexane:diethyl ether 95:5 → 6:4) gave the title compound **14** (82.0 mg, 198 µmol, 49%) as a colourless oil; R_f = 0.43 (cyclohexane/diethyl ether; 6:4); υ_{max}/cm^{-1} (thin film) 2948, 1744, 1670, 1506, 1216, 1151, 715, 702; ^{1}H NMR (400 MHz, $CDCl_3$) δ 7.57 – 7.47 (2H, m), 7.44 – 7.21 (8H, m), 5.65 (1H, t, *J* = 6.4 Hz), 4.73 – 4.60 (2H, m), 3.70 (1H, dd, *J* = 13.8, 5.6 Hz), 3.56 (1H, dd, *J* = 13.8, 7.9 Hz), 1.75 (3H, s); ^{13}C{^{1}H} NMR (101 MHz, $CDCl_3$) δ 203.6, 155.1, 140.9, 135.3, 132.7, 130.2, 129.4, 128.3, 127.9, 126.5, 95.8, 74.6, 56.2, 51.4, 20.5. HRMS (ESI) m/z: [M + H]$^+$ calcd for $C_{19}H_{19}{}^{35}Cl_3NO_3$, 414.0425; found, 414.0421.

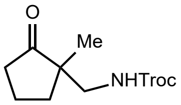

2,2,2-Trichloroethyl [(1-methyl-2-oxocyclopentyl)methyl]carbamate (16a): Prepared according to general procedure E using 1-(prop-1-en-2-yl)cyclobutan-1-ol **15a** (45.0 mg, 400 µmol), 2,2,2-trichloroethyl {[4-(trifluoromethyl)benzoyl]oxy}carbamate **6a** (183 mg, 480 µmol), [Ir(ppy)$_3$] (2.7 mg, 4.00 µmol, 1 mol%) and acetonitrile (2 mL). Purification by flash column chromatography (SiO_2, cyclohexane:ethyl acetate – 20:1) gave the title compound **16a** (94.0 mg, 310 µmol, 78%) as a colourless oil; R_f = 0.51 (cyclohexane/ethyl acetate; 6:4); υ_{max}/cm^{-1} (thin film) 3348, 2964, 1727, 1519, 1236, 1147, 907, 727; ^{1}H NMR (400 MHz, $CDCl_3$) δ 5.41 (1H, br. s), 4.76 – 4.69 (2H, m), 3.35 (1H, dd, J = 13.9, 7.0 Hz), 3.23 (1H, dd, J = 13.9, 7.0 Hz), 2.44 – 2.29 (1H, m), 2.29 – 2.13 (1H, m), 2.02 – 1.85 (3H, m), 1.84 – 1.74 (1H, m), 1.06 (3H, s); ^{13}C{^{1}H} NMR (101 MHz, $CDCl_3$) δ 223.1, 155.3, 95.7, 74.7, 49.2, 46.3, 37.7, 34.2, 20.0, 18.7; HRMS (ESI) m/z: [M + NH_4]$^+$ calcd for $C_{10}H_{18}{}^{35}Cl_3N_2O_3$, 319.0378; found, 319.0377.

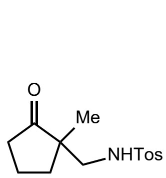

4-Methyl-*N*-[(1-methyl-2-oxocyclopentyl)methyl]benzenesulfonamide (S10): Prepared according to general procedure E using 1-(prop-1-en-2-yl)cyclobutan-1-ol **15a** (44.9 mg, 400 µmol), 1-[(4-methylphenyl)sulfonamido]pyridin-1-ium tetrafluoroborate **S5** (161 mg, 480 µmol), Ir(ppy)$_3$ (2.7 mg, 4.00 µmol, 1 mol%) and acetonitrile (2 mL). Purification by flash column chromatography (SiO_2, cyclohexane:ethyl acetate – 4:1) gave the title compound **S10** (77.2 mg, 274 µmol, 69%) as a colourless

oil; R_f = 0.13 (cyclohexane/ethyl acetate; 4:1); υ_{max}/cm^{-1} (thin film) 3378, 2964, 1731, 1455, 1329, 1159, 1092, 1075, 847, 663; ^{1}H NMR (400 MHz, $CDCl_3$) δ 7.71 (2H, d, *J* = 8.1 Hz), 7.30 (2H, d, *J* = 8.0 Hz), 4.95 (1H, t, *J* = 6.8 Hz), 2.97 (1H, dd, *J* = 12.7, 7.3 Hz), 2.84 (1H, dd, *J* = 12.6, 6.2 Hz), 2.42 (3H, s), 2.35 (1H, dddd, *J* = 19.2, 8.5, 4.2, 1.7 Hz), 2.14 (1H, dt, *J* = 18.8, 9.0 Hz), 2.07 – 1.83 (3H, m), 1.80 – 1.70 (1H, m), 1.02 (3H, s); ^{13}C{^{1}H} NMR (101 MHz, $CDCl_3$) δ 223.1, 143.6, 137.0, 129.9, 127.1, 48.6, 48.4, 37.7, 33.9, 21.7, 20.2, 18.7; HRMS (ESI) m/z: [M + Na]$^+$ calcd for $C_{14}H_{19}NNaO_3S$, 304.0978; found, 304.0980.

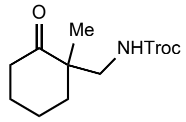

2,2,2-Trichloroethyl [(1-methyl-2-oxocyclohexyl)methyl]carbamate (16b): Prepared according to general procedure E using 1-(prop-1-en-2-yl)cyclopentan-1-ol **15b** (51.0 mg, 400 μmol), 2,2,2-trichloroethyl {[4-(trifluoromethyl)benzoyl]oxy}carbamate **6** (183 mg, 480 μmol), [Ir(ppy)$_3$] (2.7 mg, 4.00 μmol, 1 mol%) and acetonitrile (2 mL). Purification by flash column chromatography (SiO_2, cyclohexane:diethyl ether – 8:2 → 6:4) gave the title compound **16b** (93.0 mg, 294 μmol, 73%) as a colourless oil; R_f = 0.28 (cyclohexane/diethyl ether; 6:4); υ_{max}/cm^{-1} (thin film) 3336, 2937, 1733, 1699, 1509, 1218, 1144, 810, 723, 567; ^{1}H NMR (400 MHz, $CDCl_3$) δ 5.56 (1H, br. s), 4.77 – 4.65 (2H, m), 3.31 – 3.17 (2H, m), 2.57 – 2.45 (1H, m), 2.34 – 2.24 (1H, m), 2.08 – 1.99 (1H, m), 1.89 – 1.56 (5H, m), 1.19 (3H, s); ^{13}C{^{1}H} NMR (101 MHz, $CDCl_3$) δ 216.5, 155.1, 95.7, 74.5, 49.9, 48.0, 38.7, 36.6, 27.5, 20.9, 20.7; HRMS (ESI) m/z: [M + NH_4]$^+$ calcd for $C_{11}H_{20}{}^{35}Cl_3N_2O_3$, 333.0534; found, 333.0536.

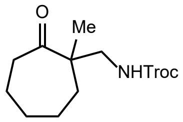

2,2,2-Trichloroethyl [(1-methyl-2-oxocycloheptyl)methyl]carbamate (16c): Prepared according to general procedure E using 1-(prop-1-en-2-yl)cyclohexan-1-ol **15c** (56.0 mg, 400 μmol), 2,2,2-trichloroethyl {[4-(trifluoromethyl)benzoyl]oxy}carbamate **6** (183 mg, 480 μmol), [Ir(ppy)$_3$] (2.7 mg, 4.00 μmol, 1 mol%) and acetonitrile (2 mL). Purification by flash column chromatography (SiO_2, cyclohexane:diethyl ether – 8:2) gave the title compound **16c** (80.0 mg, 242 μmol, 60%) as a colourless oil; R_f = 0.25 (cyclohexane/diethyl ether; 6:4); υ_{max}/cm^{-1} (thin film) 3348, 2931, 1731, 1696, 1514, 1235, 1146, 812, 724; ^{1}H NMR (500 MHz, $CDCl_3$) δ 5.54 (1H, t, *J* = 5.6 Hz), 4.76 – 4.65 (2H, m), 3.34 (1H, dd, J = 13.8, 5.3 Hz), 3.26 (1H, dd, J = 13.8, 7.7 Hz), 2.68 – 2.58 (1H, m), 2.47 – 2.38 (1H, m), 1.81 – 1.72 (2H, m), 1.70 – 1.50 (5H, m), 1.43 – 1.34 (1H, m), 1.13 (3H, s); ^{13}C{^{1}H} NMR (126 MHz, $CDCl_3$) δ 217.8, 155.2, 95.8, 74.6, 51.7, 48.4, 40.5, 36.2, 30.6, 26.6, 24.8, 21.8. HRMS (ESI) m/z: [M + NH_4]$^+$ calcd for $C_{12}H_{22}{}^{35}Cl_3N_2O_3$, 347.0691; found, 347.0689.

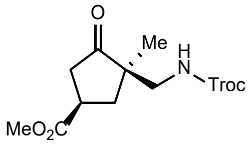

(±)-**16d**

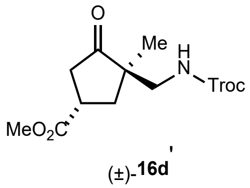

(±)-**16d'**

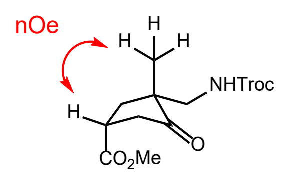

Major Diastereoisomer **16d**

Methyl 3-methyl-4-oxo-3-((((2,2,2-trichloroethoxy)carbonyl)amino)methyl)cyclopentane-1-carboxylate (16d): Prepared according to a modification of general procedure E (not stirred with 1 M aq. HCl solution before workup) using methyl 3-hydroxy-3-(prop-1-en-2-yl)cyclobutane-1-carboxylate **15d** (68.0 mg, 400 µmol), 2,2,2-trichloroethyl {[4-(trifluoromethyl)benzoyl]oxy}carbamate **6** (183 mg, 440 µmol) and [Ir(ppy)$_3$] (2.7 mg, 4.00 µmol, 1 mol%) and acetonitrile (2 mL). Purification by flash column chromatography (SiO_2, cyclohexane:diethyl ether – 95:5 → 6:4) gave an inseperable mixture of the title compounds **16d** and **16d'** (91.0 mg, 252 µmol, 63%, d.r. 2:1) as a colourless oil; R_f = 0.23 (cyclohexane/diethyl ether; 6:4); υ_{max}/cm^{-1} (thin film) 3348, 2955, 1721, 1529, 1229, 1145, 811, 721, 566; **Major Diastereoisomer 16d**: ^{1}H NMR (400 MHz, $CDCl_3$) δ 5.47 (1H, t, J = 6.1 Hz), 4.80 – 4.67 (2H, m), 3.73 (3H, s), 3.42 – 3.28 (2H, m), 3.25 – 3.10 (1H, m), 2.81 – 2.52 (2H, m), 2.17 – 1.98 (2H, m), 1.09 (3H, s); ^{13}C{^{1}H} NMR (101 MHz, $CDCl_3$) δ 219.2, 174.5, 155.3, 95.7, 74.6, 52.4, 50.7, 46.1, 40.6, 37.3, 37.2, 20.2; **Minor Diastereoisomer 16d'**: ^{1}H NMR (400 MHz, $CDCl_3$) δ 5.38 (1H, t, J = 5.5 Hz), 4.80 – 4.67 (2H, m), 3.74 (3H, s), 3.42 – 3.28 (2H, m), 3.25 – 3.10 (1H, m), 2.81 – 2.52 (2H, m), 2.40 – 2.31 (1H, m), 2.09 – 1.98 (1H, m), 1.12 (3H, s); ^{13}C{^{1}H} NMR (101 MHz, $CDCl_3$) δ 219.0, 174.9, 155.3, 95.6, 74.7, 52.4, 49.8, 46.4, 40.5, 37.7, 37.2, 20.9; HRMS (ESI) m/z: $[M + H]^+$ calcd for $C_{12}H_{17}{}^{35}Cl_3NO_5$, 360.0167; found, 360.0169.

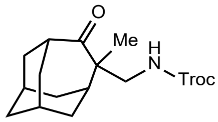

2,2,2-trichloroethyl{[(1R,3s,6r,8S)-4-methyl-5-oxotricyclo[4.3.1.13,8]undecan-4-yl]methyl}carbamate (16e): Prepared according to a modification of general procedure E (reaction time: 48 h) using 2-(prop-1-en-2-yl)adamantan-2-ol **15e** (90.0 mg, 400 µmol), 2,2,2-trichloroethyl {[4-(trifluoromethyl)benzoyl]oxy}carbamate **6** (183 mg, 480 µmol), [Ir(ppy)$_3$] (2.7 mg, 4.00 µmol, 1 mol%) and acetonitrile (2 mL). Purification by flash column chromatography (SiO_2, cyclohexane:diethyl ether 8:2) gave the title compound **16e** (68.0 mg, 178 µmol, 44%) as a colourless crystalline solid; m.p. 79 – 81 °C, R_f = 0.40 (cyclohexane/diethyl ether; 6:4); υ_{max}/cm^{-1} (thin film) 2915, 2856, 1739, 1240, 1150, 724; ^{1}H NMR (400 MHz, $CDCl_3$) δ 4.78 (1H, t, J = 5.4 Hz, NH), 4.67 (2H, s), 3.57 (2H, d, J = 5.8 Hz), 2.24 – 2.15 (5H, m), 2.06 – 1.94 (2H, m), 1.90 – 1.80 (2H, m), 1.76 – 1.60 (9H, m); ^{13}C{^{1}H} NMR (101 MHz, $CDCl_3$) δ 213.2, 154.8, 95.6, 74.7, 57.3, 43.7, 38.0, 35.0, 32.5, 30.4, 27.5, 26.9, 24.9; HRMS (ESI) m/z: $[M + NH_4]^+$ calcd for $C_{16}H_{26}{}^{35}Cl_3N_2O_3$, 339.1004; found, 339.1005.

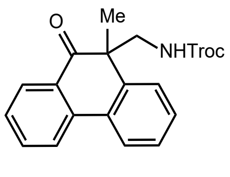

2,2,2-Trichloroethyl [(9-methyl-10-oxo-9,10-dihydrophenanthren-9-yl)methyl]carbamate (16f): Prepared according to a modification of general procedure E (reaction time: 48 h) using 9-(prop-1-en-2-yl)-9*H*-fluoren-9-ol **15f** (89.0 mg, 400 µmol), 2,2,2-trichloroethyl {[4-(trifluoromethyl)benzoyl]oxy}carbamate **6** (183 mg, 480 µmol), [Ir(ppy)$_3$] (2.7 mg, 4.00 µmol, 1 mol%) and acetonitrile (2 mL). Purification by flash column chromatography (SiO_2, cyclohexane:diethyl ether 9:1) gave the title compound **16f** (130 mg, 316 µmol, 79%) as a colourless oil; R_f = 0.38 (cyclohexane/diethyl ether; 6:4); υ_{max}/cm^{-1} (thin film) 1732, 1676, 1514, 1219, 905, 723; ^{1}H NMR (400 MHz, $CDCl_3$) δ 8.07 – 7.96 (3H, m), 7.69 (1H, td, *J* = 7.7, 1.5 Hz), 7.63 – 7.53 (1H, m), 7.47 – 7.33 (3H, m), 5.24 (1H, br. t, *J* = 6.0 Hz), 4.71 – 4.52 (2H, m), 3.95 (1H, dd, *J* = 13.8, 6.0 Hz), 3.79 (1H, dd, *J* = 13.8, 6.9 Hz), 1.50 (3H, s); ^{13}C{^{1}H} NMR (101 MHz, $CDCl_3$) δ 202.6, 154.7, 139.5, 137.3, 135.0, 130.3, 129.6, 128.8, 128.5, 127.9, 126.9, 124.5, 123.2, 95.6, 74.5, 52.5, 47.7, 24.8; HRMS (ESI) m/z: $[M + H]^+$ calcd for $C_{19}H_{17}{}^{35}Cl_3NO_3$, 412.0269; found, 412.0262.

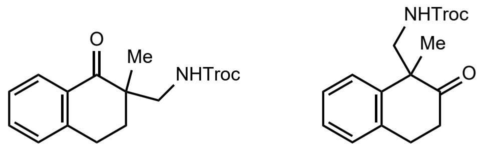

16g **16g'**

2,2,2-Trichloroethyl [(2-methyl-1-oxo-1,2,3,4-tetrahydronaphthalen-2-yl)methyl]carbamate (16g) and 2,2,2-trichloroethyl ((1-methyl-2-oxo-1,2,3,4-tetrahydronaphthalen-1-yl)methyl)carbamate (16g'): Prepared according to general procedure E using 1-(prop-1-en-2-yl)-2,3-dihydro-1*H*-inden-1-ol **15g** (70.0 mg, 400 µmol), 2,2,2-trichloroethyl {[4-(trifluoromethyl)benzoyl]oxy}carbamate **6** (183 mg, 480 µmol), [Ir(ppy)$_3$] (2.7 mg, 4.00 µmol, 1 mol%) and acetonitrile (2 mL). Purification by flash column chromatography (SiO_2, cyclohexane:diethyl ether 9:1 → 6:4) gave the title compound **16g** (84.0 mg, 230 µmol, 58%) as a colourless oil and regioisomer **16g'** (57.0 mg, 156 µmol, 39%) as a colourless oil.

2,2,2-Trichloroethyl [(2-methyl-1-oxo-1,2,3,4-tetrahydronaphthalen-2-yl)methyl]carbamate (16g): R_f = 0.51 (cyclohexane/diethyl ether; 6:4); υ_{max}/cm^{-1} (thin film) 3336, 2948, 1707, 1513, 1225, 1147, 761, 722; ^{1}H NMR (400 MHz, $CDCl_3$) δ 8.01 (1H, d, *J* = 7.5 Hz), 7.50 (1H, dd, *J* = 7.5, 1.0 Hz), 7.32 (1H, dd, *J* = 7.5, 1.0 Hz), 7.28 – 7.21 (1H, m), 5.65 (1H, t, *J* = 6.1 Hz), 4.77 (1H, d, J = 12.0 Hz), 4.71 (1H, d, J = 12.0 Hz) (2H, q, *J* = 12.1 Hz), 3.57 (1H, dd, *J* = 13.8, 6.6 Hz), 3.36 (1H, dd, *J* = 13.8, 6.6 Hz), 3.22 – 3.10 (1H, m), 3.01 – 2.89 (1H, m), 2.25 – 2.11 (1H, m), 1.95 – 1.84 (1H, m), 1.21 (3H, s); ^{13}C{^{1}H} NMR (101 MHz, $CDCl_3$) δ 202.8, 155.3, 143.3, 133.9, 131.4, 129.0, 128.0, 127.0, 95.8,

74.7, 48.1, 46.3, 32.1, 25.3, 19.2; HRMS (ESI) m/z: $[M + H]^+$ calcd for $C_{15}H_{17}{}^{35}Cl_3NO_3$, 364.0269; found, 364.0264.

2,2,2-Trichloroethyl [(1-methyl-2-oxo-1,2,3,4-tetrahydronaphthalen-1-yl)methyl]carbamate (16g'): R_f = 0.48 (cyclohexane/diethyl ether; 6:4); υ_{max}/cm^{-1} (thin film) 3350, 2929, 1731, 1674, 1510, 1223, 1143, 723; ^{1}H NMR (400 MHz, $CDCl_3$) δ 7.40 (1H, d, *J* = 7.6 Hz), 7.30 (t, *J* = 7.5 Hz), 7.27 – 7.14 (2H, m), 5.22 (1H, t, *J* = 5.3 Hz), 4.70 – 4.57 (2H, m), 3.80 (1H, dd, *J* = 13.9, 6.5 Hz), 3.62 (1H, dd, *J* = 13.9, 6.5 Hz), 3.17 – 3.07 (2H, m), 2.85 – 2.75 (1H, m), 2.70 – 2.60 (1H, m), 1.45 (3H, s); $^{13}C\{^1H\}$ NMR (101 MHz, $CDCl_3$) δ 214.3, 154.7, 139.4, 136.2, 128.5, 127.5, 127.3, 126.9, 95.7, 74.5, 52.5, 48.1, 37.7, 27.0, 24.2. HRMS (ESI) m/z: $[M + Na]^+$ calcd for $C_{15}H_{16}{}^{35}Cl_3NNaO_3$, 386.0088; found, 386.0089.

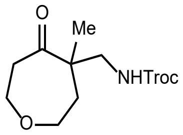

2,2,2-Trichloroethyl ((4-methyl-5-oxooxepan-4-yl)methyl)carbamate (16h): Whilst we were unable to cleanly isolate the trace product **16h**. A sample of sufficient purity for characterization by $^1H/^{13}C$ NMR spectroscopy and HRMS was obtained following attempted purification by flash column chromatography (SiO_2, cyclohexane:ethyl acetate – 15:1 → 3:2); ^{1}H NMR (400 MHz, $CDCl_3$) 5.51 – 5.42 (1H, m), 4.71 (2H, s), 3.88 – 3.79 (2H, m), 3.68 (1H, ddd, *J* = 12.1, 8.6, 3.3 Hz), 3.62 (1H, ddd, *J* = 13.3, 7.7, 2.3 Hz), 3.36 (1H, dd, *J* = 13.9, 6.3 Hz), 3.33 (1H, dd, *J* = 13.9, 6.9 Hz), 2.93 (1H, ddd, *J* = 13.1, 8.6, 3.9 Hz), 2.65 (1H, ddd, *J* = 13.1, 6.7, 3.3 Hz), 1.97 (1H, ddd, *J* = 15.7, 8.0, 2.3 Hz), 1.79 (1H, ddd, *J* = 15.7, 7.7, 2.1 Hz); $^{13}C\{^1H\}$ NMR (101 MHz, $CDCl_3$) 214.4, 155.2, 95.7, 74.7, 68.2, 66.8, 51.3, 48.5, 44.7, 36.5, 21.8; HRMS (ESI) m/z: $[M + Na]^+$ calcd for $C_{11}H_{16}{}^{35}Cl_3NNaO_4$, 354.0037; found, 354.0036.

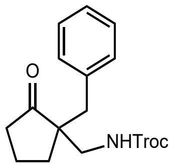

2,2,2-Trichloroethyl ((1-benzyl-2-oxocyclopentyl)methyl)carbamate (16i): Prepared according to general procedure E using 1-(3-phenylprop-1-en-2-yl)cyclobutan-1-ol **15i** (76.0 mg, 400 μmol), {[4-(trifluoromethyl)benzoyl]oxy}carbamate **6** (183 mg, 480 μmol), [Ir(ppy)$_3$] (2.7 mg, 4.00 μmol, 1 mol%) and acetonitrile (2 mL). Purification by flash column chromatography (SiO_2, cyclohexane:ethyl acetate – 20:1 → 9:1) gave the title compound **16i** (100 mg, 264 μmol, 66%) as a colourless oil; R_f = 0.48 (cyclohexane/ethyl acetate; 6:4); υ_{max}/cm^{-1} (thin film) 2927, 1726, 1515, 1230, 1148, 905, 726; ^{1}H NMR (400 MHz, $CDCl_3$) δ 7.32 – 7.20 (3H, m), 7.15 – 7.07 (2H, m), 5.45 (1H, t, *J* = 6.1 Hz), 4.80 – 4.67 (2H, m), 3.41 (1H, dd, *J* = 13.9, 5.2 Hz), 3.22 (1H, dd, *J* = 13.9, 5.2 Hz), 2.89 (1H, d, *J* = 13.5 Hz), 2.67

(1H, d, J = 13.5 Hz), 2.31 – 2.20 (1H, m), 2.17 – 2.06 (1H, m), 2.05 – 1.95 (1H, m), 1.88 – 1.72 (2H, m), 1.72 – 1.52 (1H, m); $^{13}C\{^1H\}$ NMR (101 MHz, $CDCl_3$) δ 223.0, 155.1, 136.5, 130.2, 128.7, 127.1, 95.7, 74.7, 53.5, 45.5, 39.3, 38.8, 30.9, 18.8; HRMS (ESI) m/z: $[M + H]^+$ calcd for $C_{16}H_{19}{}^{35}Cl_3NO_3$, 378.0425; found, 378.0414.

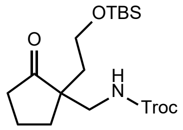

2,2,2-Trichloroethyl ((1-(2-((tert-butyldimethylsilyl)oxy)ethyl)-2-oxocyclopentyl)methyl)carbamate (16j): Prepared according to a modification of general procedure E (not stirred with 1 M aq. HCl solution before workup) using 1-{4-[(*tert*-butyldimethylsilyl)oxy]but-1-en-2-yl}cyclobutan-1-ol **15j** (103 mg, 400 μmol), 2,2,2-trichloroethyl {[4-(trifluoromethyl)benzoyl]oxy}carbamate **6** (183 mg, 440 μmol) and [Ir(ppy)$_3$] (2.7 mg, 4.00 μmol, 1 mol%), and acetonitrile (2 mL). Purification by column chromatography (SiO_2, cyclohexane:diethyl ether – 8:2) gave the title compound **16j** (151 mg, 338 μmol, 85%) as a colourless oil; R_f = 0.31 (cyclohexane/diethyl ether; 6:4); υ_{max}/cm^{-1} (thin film) 2953, 2930, 1727, 1144, 1093, 833, 775, 725; ^1H NMR (400 MHz, $CDCl_3$) δ 5.91 (1H, t, J = 5.9 Hz), 4.72 – 4.65 (2H, m), 3.82 – 3.73 (1H, m), 3.67 – 3.60 (1H, m), 3.38 (1H, dd, J = 14.0, 7.8 Hz), 3.19 (1H, dd, J = 14.0, 7.8 Hz), 2.34 – 2.24 (2H, m), 1.97 – 1.85 (4H, m), 1.77 – 1.62 (2H, m), 0.87 (9H, s), 0.05 (6H, s); $^{13}C\{^1H\}$ NMR (101 MHz, $CDCl_3$) δ 221.5, 155.1, 95.8, 74.6, 59.4, 51.4, 44.6, 37.9, 36.4, 32.4, 26.0, 18.8, 18.3, −5.4; HRMS (ESI) m/z: $[M + H]^+$ calcd for $C_{17}H_{31}{}^{35}Cl_3NO_4Si$, 446.1082; found, 446.1081.

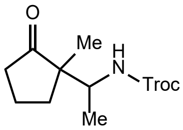

2,2,2-Trichloroethyl [1-(1-methyl-2-oxocyclopentyl)ethyl]carbamate (16k): Prepared according to general procedure E using 1-(but-2-en-2-yl)cyclobutan-1-ol **15k** (51 mg, 400 μmol), 2,2,2-trichloroethyl {[4-(trifluoromethyl)benzoyl]oxy}carbamate **6** (183 mg, 480 μmol), [Ir(ppy)$_3$] (2.7 mg, 4.00 μmol, 1 mol%) and acetonitrile (2 mL). Purification by flash column chromatography (SiO_2, cyclohexane:diethyl ether 9:1 → 6:4) gave the major diastereoisomer of the title compound **16k** (32.0 mg, 101 μmol, 25%) as a colourless oil and the minor diastereoisomer **16k**' (29.0 mg, 92.0 μmol, 23%) as a colourless oil (d.r. 1:1).

Major Diastereoisomer 16k: R_f = 0.28 (cyclohexane/diethyl ether; 6:4); υ_{max}/cm^{-1} (thin film) 3339, 2964, 1721, 1509, 1230, 1114, 735; ^1H NMR (400 MHz, $CDCl_3$) δ 5.83 (1H, d, J = 9.1 Hz), 4.80 – 4.67 (2H, m), 3.91 – 3.79 (1H, dq, J = 9.9, 6.8 Hz), 2.45 – 2.34 (1H, m), 2.25 – 2.11 (1H, m), 2.09 – 1.83

(3H, m), 1.82 – 1.71 (1H, m), 1.20 – 1.09 (6H, m); $^{13}C\{^{1}H\}$ NMR (101 MHz, $CDCl_3$) δ 223.8, 154.5, 95.9, 74.6, 51.9, 51.5, 39.1, 34.3, 20.8, 19.0, 17.3; HRMS (ESI) m/z: $[M + NH_4]^+$ calcd for $C_{11}H_{20}{}^{35}Cl_3N_2O_3$, 333.0534; Found, 333.0539 (1.50 ppm error).

Minor Diastereoisomer 16k': R_f = 0.26 (cyclohexane/diethyl ether; 6:4); υ_{max}/cm^{-1} 3330, 2966, 1729, 1535, 1237, 1154, 736; ^{1}H NMR (400 MHz, $CDCl_3$) δ 4.91 (1H, d, J = 7.3 Hz), 4.71 (2H, d, J = 2.4 Hz), 4.02 – 3.89 (1H, m), 2.41 – 2.31 (1H, m), 2.30 – 2.15 (1H, m), 2.08 – 1.81 (3H, m), 1.77 – 1.65 (1H, m), 1.18 (3H, d, J = 6.8 Hz), 1.07 (3H, s); $^{13}C\{^{1}H\}$ NMR (101 MHz, $CDCl_3$) δ 221.6, 154.5, 95.8, 74.7, 52.2, 51.2, 38.4, 33.1, 19.6, 18.6, 16.8; HRMS (ESI) m/z: $[M + H]^+$ calcd for $C_{11}H_{17}{}^{35}Cl_3NO_3$, 316.0269; found, 316.0268 (0.10 ppm error).

Note: It was not possible to establish the relative stereochemistry of the major and minor diastereoisomers.

5. Removal of the Troc Protecting Group

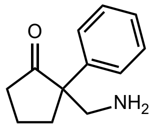

2-(Aminomethyl)-2-phenylcyclopentan-1-one (17): To a stirred solution of 2,2,2-trichloroethyl [(2-oxo-1-phenylcyclopentyl)methyl]carbamate **11a** (73 mg, 200 μmol, 1.0 equiv.) in AcOH (2 mL, 0.1 M), was added Zn dust (131 mg, 2.0 mmol, 10.0 equiv) at room temperature in a single portion, then the reaction was stirred at rt for 24 h. The reaction mixture was passed throuth a pad of celite which was washed with MeOH (2 mL), followed by removal of solvents *in vacuo* to give the crude prouduct. This residual gum was triturated with diethyl ether (3 × 2 mL) and the solvent was removed. The resulting white solids were dissolved in 10% aq. NaOH solution (5 mL) and extracted with DCM (3 × 3 mL). The combined organic phases were dried ($MgSO_4$), filtered and concentrated *in vacuo* to give the title compound **17** (35.5 mg, 187 μmol, 94%) as a colourless oil; υ_{max}/cm^{-1} (thin film) 3379, 2961, 1728, 1597, 1495, 1403, 1154, 757, 701; ^1H NMR (400 MHz, $CDCl_3$) δ 7.41 – 7.31 (4H, m), 7.29 – 7.23 (1H, m), 2.97 (2H, s), 2.49 (1H, dddd, *J* = 12.8, 6.2, 4.7, 1.3 Hz), 2.38 – 2.17 (3H, m), 2.01 – 1.77 (2H, m), 1.14 (2H, br s); $^{13}C\{^1H\}$ NMR (101 MHz, $CDCl_3$) δ 220.1, 139.0, 128.9, 127.2, 127.0, 59.3, 49.9, 38.7, 33.4, 18.9; HRMS (ESI) m/z: $[M + H]^+$ calcd for $C_{12}H_{16}NO$, 190.1226; found, 190.1228.

6. Crystal Structure of Compound **11d**

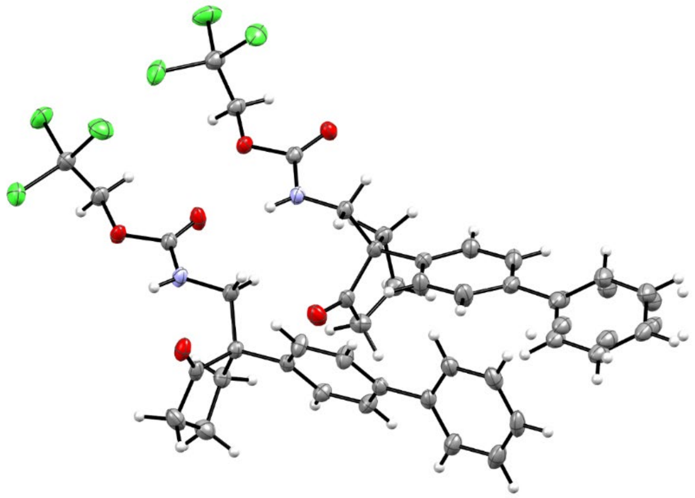

Crystal Data and Structure Refinement for Compound 11d (CCDC No. 2237285)

Identification code	MDJCCB
Empirical formula	$C_{21}H_{20}Cl_3NO_3$
Formula weight	440.73
Temperature/K	120(2)
Crystal system	triclinic
Space group	P-1
a/Å	11.0267(4)
b/Å	11.9294(5)
c/Å	18.1545(6)
α/°	77.691(3)
β/°	76.621(3)
γ/°	62.808(4)
Volume/Å^3	2050.51(15)
Z	4
ρ_{calc}g/cm^3	1.428
μ/mm^{-1}	4.234
F(000)	912.0
Crystal size/mm^3	0.116 × 0.053 × 0.034
Radiation	Cu Kα (λ = 1.54184)
2Θ range for data collection/°	8.398 to 145.638
Index ranges	$-13 \leq h \leq 13$, $-14 \leq k \leq 14$, $-20 \leq l \leq 22$
Reflections collected	31072
Independent reflections	7873 [R_{int} = 0.0385, R_{sigma} = 0.0343]
Data/restraints/parameters	7873/297/524
Goodness-of-fit on F^2	1.058
Final R indexes [I>=2σ (I)]	R_1 = 0.0375, wR_2 = 0.0930
Final R indexes [all data]	R_1 = 0.0456, wR_2 = 0.0970
Largest diff. peak/hole / e Å^{-3}	0.31/-0.42

Single Crystal X-ray Diffraction Method

Single crystals were selected and mounted using Fomblin® (YR-1800 perfluoropolyether oil) on a polymer-tipped MiTeGen MicroMountTM and cooled rapidly to 120 K in a stream of cold N_2 using an Oxford Cryosystems open flow cryostat.[69] Single crystal X-ray diffraction data were collected on an Oxford Diffraction GV1000 (TitanS2 CCD area detector, mirror-monochromated Cu-Kα radiation source; λ = 1.54184 Å, ω scans). Cell parameters were refined from the observed positions of all strong reflections and absorption corrections were applied using a Gaussian numerical method with beam profile correction (CrysAlisPro).[70] Structures were solved within Olex2[71] by dual space iterative methods (SHELXT)[72] and all non-hydrogen atoms refined by full-matrix least-squares on all unique F2 values with anisotropic displacement parameters (SHELXL).[73] Structures were checked with checkCIF.[74] CCDC-2237285 contains the supplementary data for these compounds. These data can be obtained free of charge from The Cambridge Crystallographic Data Centre via www.ccdc.cam.ac.uk/data_request/cif.

_refine_special_details

Conformational disorder is observed in phenyl ring moiety C23B/C-C28B/C with the occupancies of two disorder components refined and constrained to sum to unity resulting in values of 0.82(1) and 0.18(1) respectively. The geometries of the two disorder components were restrained to be similar and planar (SADI, FLAT) and their pivot atoms C23B/C coplanar with the adjacent phenyl ring (FLAT). Rigid bond and similarity restraints were applied to the anisotropic displacement parameters of all the disordered atoms (RIGU, SIMU). Additionally, the anisotropic displacement parameters of each adjacent pair of disordered carbon atoms were constrained to be identical (EADP). All hydrogen atoms in the structure were observed in the electron density map. Amide hydrogen atoms H9A and H9B were refined with their N-H distances restrained to target values of 0.88 Å (DFIX, esd 0.02 Å). All other hydrogen atoms were geometrically placed and refined with a riding model.

7. References

[1] V. Froidevaux, C. Negrell, S. Caillol, J.-P. Pascault, B. Boutevin, *Chem. Rev.* **2016**, *116*, 14181–14224.

[2] C. Chen, B. Dyck, B. A. Fleck, A. C. Foster, J. Grey, F. Jovic, M. Mesleh, K. Phan, J. Tamiya, T. Vickers, M. Zhang, *Bioorg. Med. Chem. Lett.* **2008**, *18*, 1346–1349.

[3] T. R. Belliotti, T. Capiris, I. V. Ekhato, J. J. Kinsora, M. J. Field, T. G. Heffner, L. T. Meltzer, J. B. Schwarz, C. P. Taylor, A. J. Thorpe, M. G. Vartanian, L. D. Wise, T. Zhi-Su, M. L. Weber, D. J. Wustrow, *J. Med. Chem.* **2005**, *48*, 2294–2307.

[4] S. A. Lawrence, *Amines: Synthesis, Properties and Applications*, Cambridge University Press, **2004**.

[5] Y. Y. Jung, J.-Y. Um, O. Nasif, S. A. Alharbi, G. Sethi, K. S. Ahn, *Phytomedicine* **2021**, *87*, 153574.

[6] B. Wan, Z. Lu, Z. Wu, C. Cheng, Y. Zhang, *Org. Lett.* **2021**, *23*, 1269–1274.

[7] S. Zhang, Z. Yan, Y. Li, Y. Gong, X. Lyu, J. Lou, D. Zhang, X. Meng, Y. Zhao, *J. Med. Chem.* **2022**, *65*, 6207–6230.

[8] Z.-L. Song, C.-A. Fan, Y.-Q. Tu, *Chem. Rev.* **2011**, *111*, 7523–7556.

[9] W. Z. Weng, B. Zhang, *Chem. Eur. J.* **2018**, *24*, 10934–10947.

[10] X.-M. Zhang, B.-S. Li, S.-H. Wang, K. Zhang, F.-M. Zhang, Y.-Q. Tu, *Chem. Sci.* **2021**, *12*, 9262–9274.

[11] S. Kumar Nanda, *Adv. Synth. Catal.* **2023**, *365*, 834–853.

[12] X. Z. Shu, M. Zhang, Y. He, H. Frei, F. D. Toste, *J. Am. Chem. Soc.* **2014**, *136*, 5844–5847.

[13] B. Sahoo, J. Li, F. Glorius, *Angew. Chem. Int. Ed.* **2015**, *54*, 11577–11580.

[14] S. Yao, K. Zhang, Q. Q. Zhou, Y. Zhao, D. Q. Shi, W. J. Xiao, *Chem. Commun.* **2018**, *54*, 8096–8099.

[15] M. Liu, H. Huang, Y. Chen, *Chinese J. Chem.* **2018**, *36*, 1209–1212.

[16] J. H. Kim, D. Y. Kim, *Synth. Commun.* **2020**, *50*, 207–216.

[17] S. B. Woo, D. Y. Kim, *J. Fluor. Chem.* **2015**, *178*, 214–218.

[18] C. W. Suh, D. Y. Kim, *Tetrahedron Lett.* **2015**, *56*, 5661–5664.

[19] G. Bergonzini, C. Cassani, H. Lorimer-Olsson, J. Hörberg, C.-J. Wallentin, *Chem. Eur. J.* **2016**, *22*, 3292–3295.

[20] R. Honeker, R. A. Garza-Sanchez, M. N. Hopkinson, F. Glorius, *Chem. Eur. J.* **2016**, *22*, 4395–4399.

[21] S. J. Kwon, Y. J. Kim, D. Y. Kim, *Tetrahedron Lett.* **2016**, *57*, 4371–4374.

[22] S. J. Kwon, D. Y. Kim, *Org. Lett.* **2016**, *18*, 4562–4565.

[23] J.-J. Zhang, Y.-B. Cheng, X.-H. Duan, *Chinese J. Chem.* **2017**, *35*, 311–315.

[24] Y. Yin, W. Z. Weng, J. G. Sun, B. Zhang, *Org. Biomol. Chem.* **2018**, *16*, 2356–2361.

[25] E. Zhang, Y.-Q. Tu, C.-A. Fan, X. Zhao, Y.-J. Jiang, S.-Y. Zhang, *Org. Lett.* **2008**, *10*, 4943–4946.

[26] Z.-M. Chen, Z. Zhang, Y.-Q. Tu, M.-H. Xu, F.-M. Zhang, C.-C. Li, S.-H. Wang, *Chem. Commun.* **2014**, *50*, 10805–10808.

[27] W.-Z. Weng, J.-G. Sun, P. Li, B. Zhang, *Chem. Eur. J.* **2017**, *23*, 9752–9755.

[28] J. Sun, G. Zheng, Y. Fu, Q. Zhang, Y. Wang, Q. Zhang, Y. Li, Q. Zhang, *Chinese J. Catal.* **2018**, *39*, 138–145.

[29] S. Alazet, J. Preindl, R. Simonet-Davin, S. Nicolai, A. Nanchen, T. Meyer, J. Waser, *J. Org. Chem.* **2018**, *83*, 12334–12356.

[30] J. Jang, D. Y. Kim, *Asian J. Org. Chem.* **2022**, *11*, e202200531.

[31] T.-C. C. Ma, S. Yao, M.-M. M. Qiao, F. Yuan, D.-Q. Q. Shi, W.-J. J. Xiao, *Org. Chem. Front.* **2021**, *8*, 4224–4229.

[32] S. Liu, Y. Huang, J. Wang, F.-L. Qing, X.-H. Xu, *J. Am. Chem. Soc.* **2022**, *144*, 1962–1970.

[33] M. D. Kärkäs, *ACS Catal.* **2017**, *7*, 4999–5022.

[34] X.-Y. Yu, Q.-Q. Zhao, J. Chen, W.-J. Xiao, J.-R. Chen, *Acc. Chem. Res.* **2020**, *53*, 1066–1083.

[35] C. Pratley, S. Fenner, J. A. Murphy, *Chem. Rev.* **2022**, *122*, 8181–8260.

[36] K. Kwon, R. T. Simons, M. Nandakumar, J. L. Roizen, *Chem. Rev.* **2022**, *122*, 2353–2428.

[37] X. Ren, Q. Guo, J. Chen, H. Xie, Q. Xu, Z. Lu, *Chem. Eur. J.* **2016**, *22*, 18695–18699.

[38] Q. Qin, Y.-Y. Han, Y.-Y. Jiao, Y. He, S. Yu, *Org. Lett.* **2017**, *19*, 2909–2912.

[39] X.-D. An, Y.-Y. Jiao, H. Zhang, Y. Gao, S. Yu, *Org. Lett.* **2018**, *20*, 401–404.

[40] X.-D. An, S. Yu, *Synthesis* **2018**, *50*, 3387–3394.

[41] X.-D. An, H. Zhang, Q. Xu, L. Yu, S. Yu, *Chinese J. Chem.* **2018**, *36*, 1147–1150.

[42] D. Zheng, A. Studer, *Angew. Chem. Int. Ed.* **2019**, *58*, 15803–15807.

[43] K. Goliszewska, K. Rybicka-Jasińska, J. Szurmak, D. Gryko, *J. Org. Chem.* **2019**, *84*, 15834–15844.

[44] X. Yi, X. Hu, *Chem. Sci.* **2021**, *12*, 1901–1906.

[45] D. Forster, W. Guo, Q. Wang, J. Zhu, *ACS Catal.* **2021**, *11*, 10871–10877.

[46] L. Flamigni, A. Barbieri, C. Sabatini, B. Ventura, F. Barigelletti, *Top. Curr. Chem.* **2007**, *281*, 143–203.

[47] J. Brioche, M. Michalak, B. Quiclet-Sire, S. Z. Zard, *Org. Lett.* **2011**, *13*, 6296–6299.

[48] C. Lai, G. Mathieu, L. P. Gabrielli Tabarez, H. Lebel, *Chem. Eur. J.* **2019**, *25*, 9423–9426.

[49] P. Patel, S. Chang, *Org. Lett.* **2014**, *16*, 3328–3331.

[50] W. Han, J. Su, J.-N. Mo, J. Zhao, *Org. Lett.* **2022**, *24*, 6247–6251.

[51] K. Miyazawa, T. Koike, M. Akita, *Chem. Eur. J.* **2015**, *21*, 11677–11680.

[52] D. L. Cain, C. McLaughlin, J. J. Molloy, C. Carpenter-Warren, N. A. Anderson, A. J. B. Watson, *Synlett* **2019**, *30*, 787–791.

[53] H. Zhang, G.-M. Zhang, S. He, Z.-C. Shi, X.-M. Zhang, J.-Y. Wang, *Org. Lett.* **2020**, *22*, 8337–8344.

[54] Q. Yin, S.-L. You, *Org. Lett.* **2014**, *16*, 1810–1813.

[55] Z. Shen, X. Pan, Y. Lai, J. Hu, X. Wan, X. Li, H. Zhang, W. Xie, *Chem. Sci.* **2015**, *6*, 6986–6990.

[56] Y. Dong, R. Li, J. Zhou, Z. Sun, *Org. Lett.* **2021**, *23*, 6387–6390.

[57] D. H. Lukamto, M. J. Gaunt, *J. Am. Chem. Soc.* **2017**, *139*, 9160–9163.

[58] H. Wu, Q. Wang, J. Zhu, *Chem. Eur. J.* **2017**, *23*, 13037–13041.

[59] A. Bunescu, Q. Wang, J. Zhu, *Angew. Chem. Int. Ed.* **2015**, *54*, 3132–3135.

[60] N. D. Willmore, R. Goodman, H. H. Lee, R. M. Kennedy, *J. Org. Chem.* **1992**, *57*, 1216–1219.

[61] Y. Liu, Y.-Y. Yeung, *Org. Lett.* **2017**, *19*, 1422–1425.

[62] A. V. Chernykh, D. S. Radchenko, A. V. Chernykh, I. S. Kondratov, N. A. Tolmachova, O. P. Datsenko, M. A. Kurkunov, S. X. Zozulya, Y. P. Kheylik, K. Bartels, C. G. Daniliuc, G. Haufe, *Eur. J. Org. Chem.* **2015**, *2015*, 6466–6471.

[63] A. Masuyama, T. Sugawara, M. Nojima, K. J. McCullough, *Tetrahedron* **2003**, *59*, 353–366.

[64] M. G. McLaughlin, M. J. Cook, *Chem. Commun.* **2014**, *50*, 3501–3504.

[65] A. Bigot, D. Breuninger, B. Breit, *Org. Lett.* **2008**, *10*, 5321–5324.

[66] G. Ali, G. D. Cuny, *J. Org. Chem.* **2021**, *86*, 10517–10525.

[67] R. Guo, G. Zhang, *J. Am. Chem. Soc.* **2017**, *139*, 12891–12894.

[68] C. F. Macrae, I. Sovago, S. J. Cottrell, P. T. A. Galek, P. McCabe, E. Pidcock, M. Platings, G. P. Shields, J. S. Stevens, M. Towler, P. A. Wood, *J. Appl. Crystallogr.* **2020**, *53*, 226–235.

[69] J. Cosier, A. M. Glazer, *J. Appl. Crystallogr.* **1986**, *19*, 105–107.

[70] Rigaku Oxford Diffraction, (**2018**), CrysAlisPro Software system, version 1.171.40.45a, Rigaku Corporation, Oxford, UK.

[71] O. V. Dolomanov, L. J. Bourhis, R. J. Gildea, J. A. K. Howard, H. Puschmann, *J. Appl. Crystallogr.* **2009**, *42*, 339–341.

[72] G. M. Sheldrick, *Acta Crystallogr. Sect. A Found. Adv.* **2015**, *71*, 3–8.

[73] G. M. Sheldrick, *Acta Crystallogr. Sect. C Struct. Chem.* **2015**, *71*, 3–8.

[74] "CheckCIF," can be found under http://checkcif.iucr.org.

8. Copies of ^{1}H, ^{13}C, and ^{19}F NMR Spectra for Novel Radical Precursors and Substrates

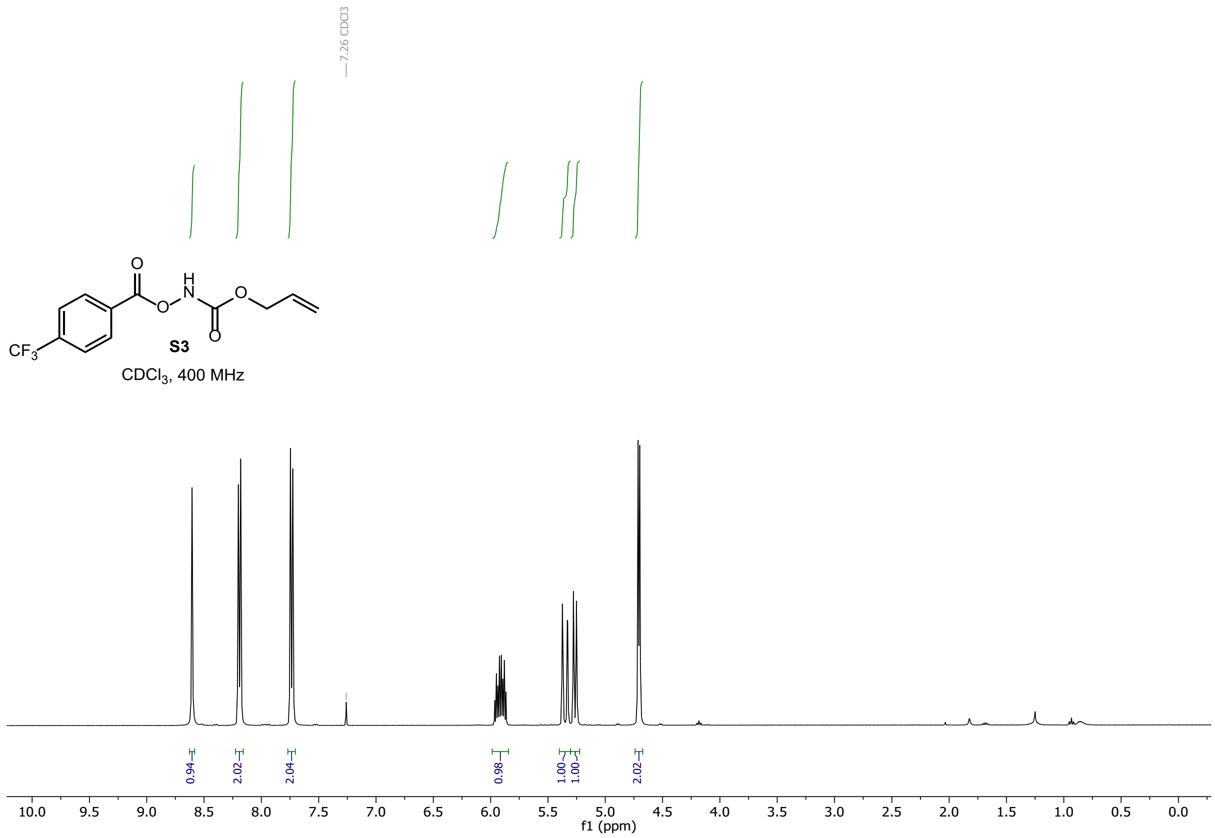

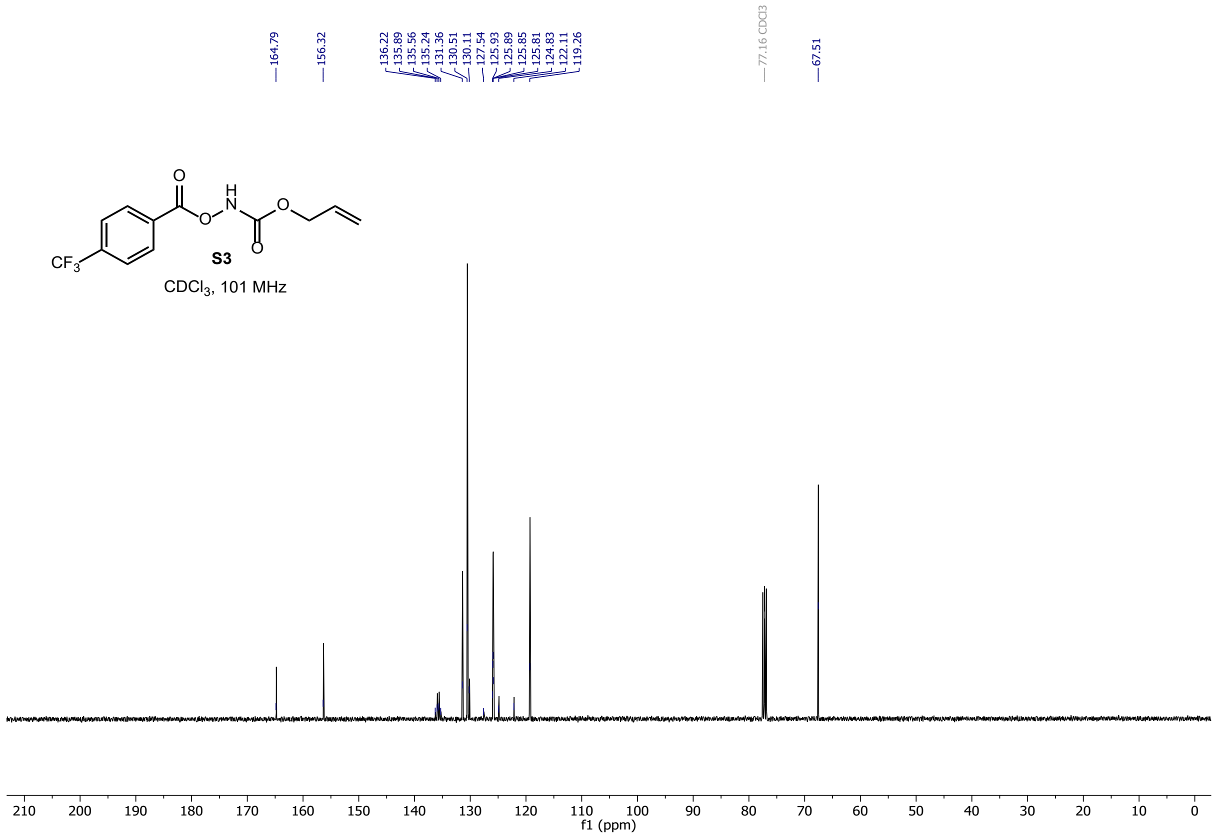

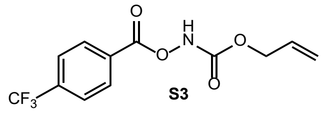
S3
CDCl3, 376 MHz

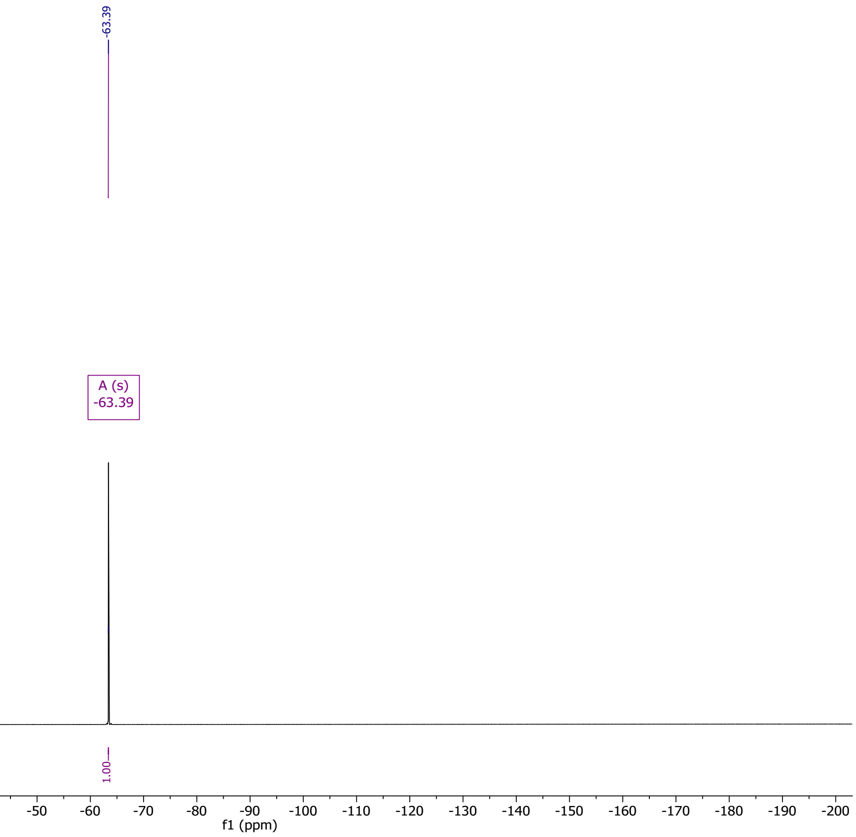
-63.39
A (s)
-63.39
1.00
20
10
0
-10
-20
-30
-40
-50
-60
-70
-80
-90
-100
-110
-120
-130
-140
-150
-160
-170
-180
-190
-200
f1 (ppm)

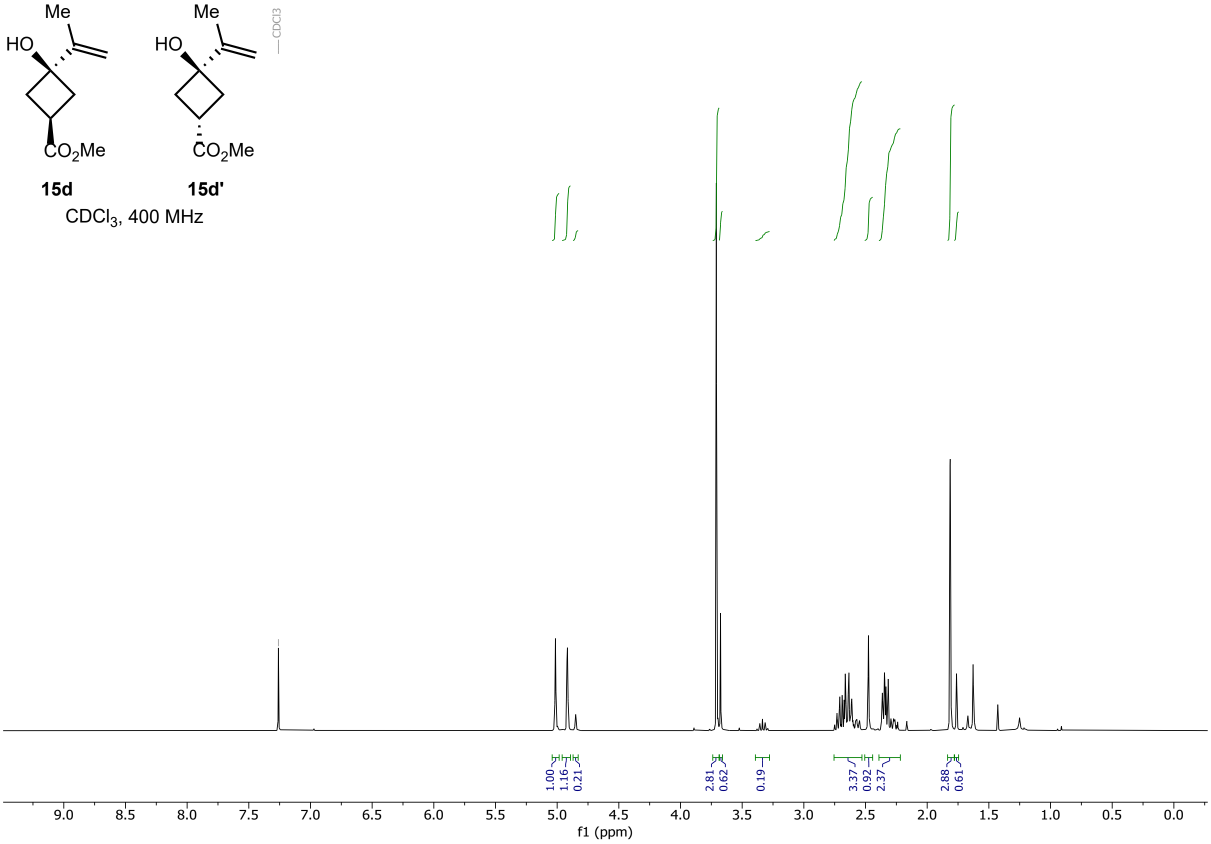
Me
HO
CO2Me
15d
15d'
CDCl3, 400 MHz
CDCl3
1.00
1.16
0.21
2.81
0.62
0.19
3.37
0.92
2.37
2.88
0.61
f1 (ppm)

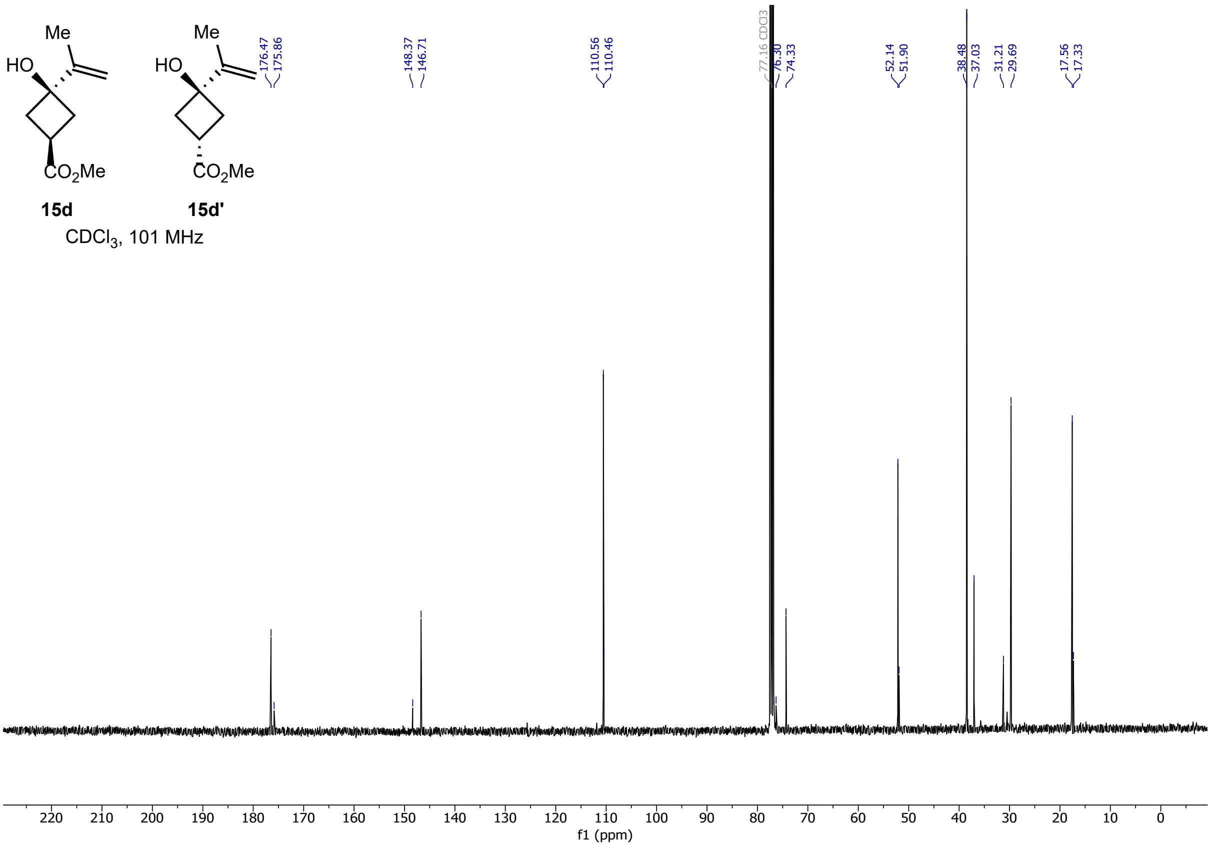
Me
HO
CO2Me
15d
15d'
CDCl3, 101 MHz
176.47
175.86
148.37
146.71
110.56
110.46
77.16 CDCl3
76.30
74.33
52.14
51.90
38.48
37.03
31.21
29.69
17.56
17.33
f1 (ppm)

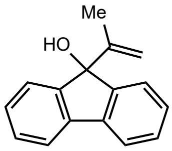

15f
$CDCl_3$, 400 MHz

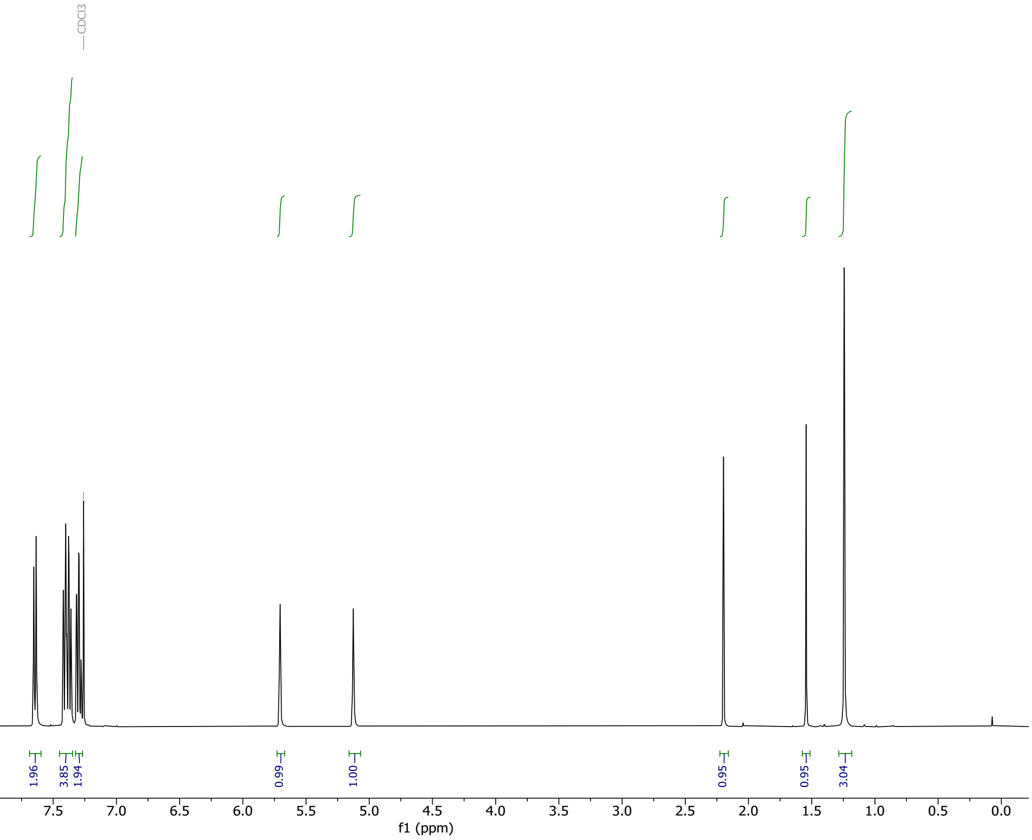

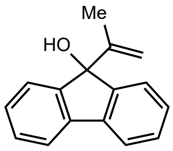

15f
$CDCl_3$, 101 MHz

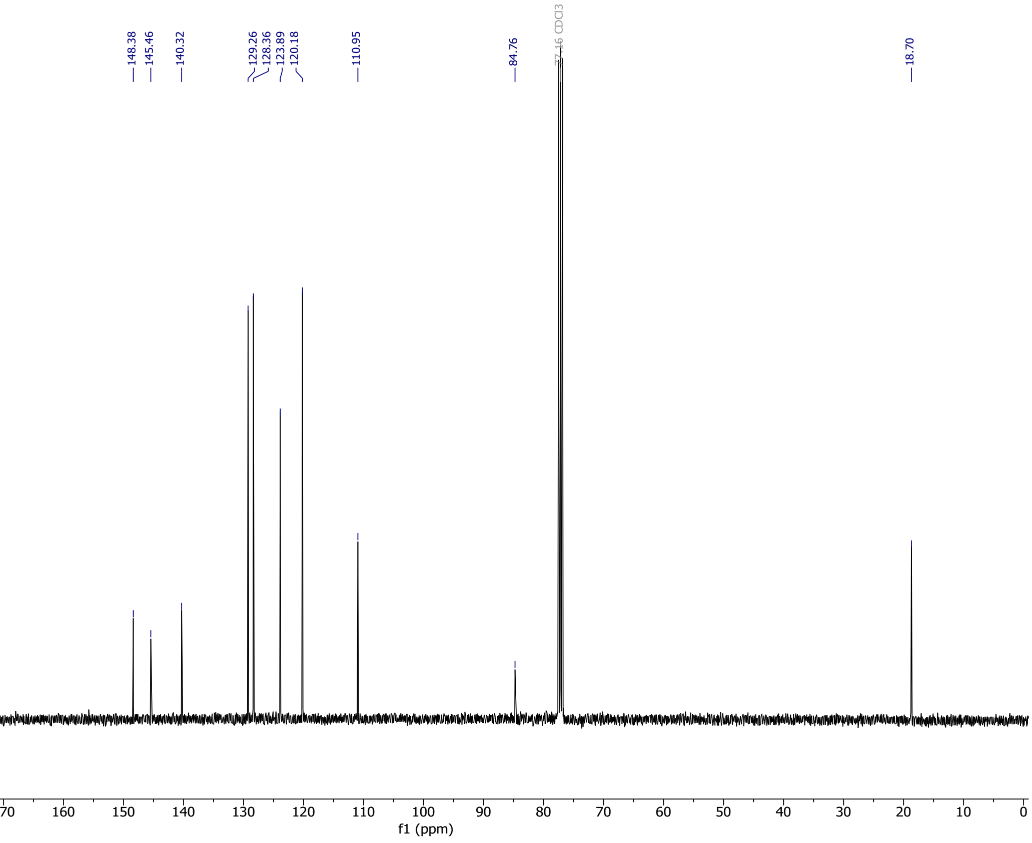

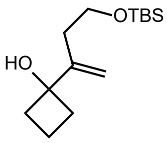

15j
$CDCl_3$, 400 MHz

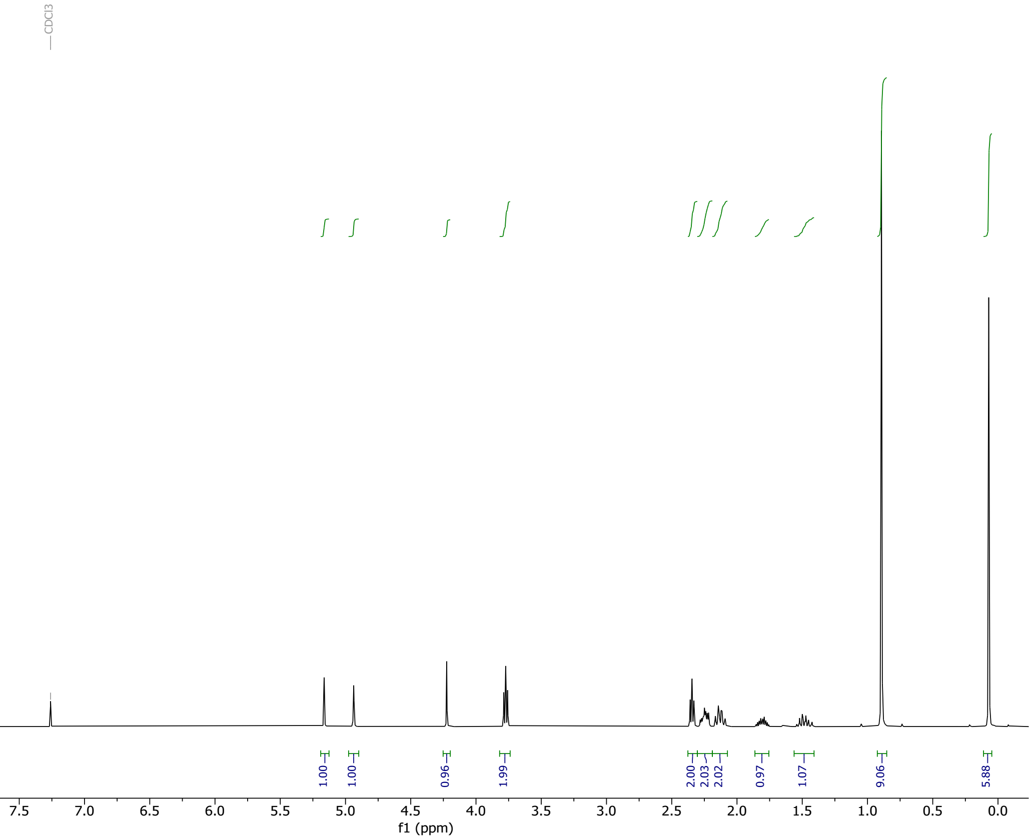

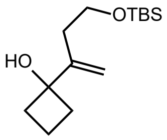

15j
$CDCl_3$, 101 MHz

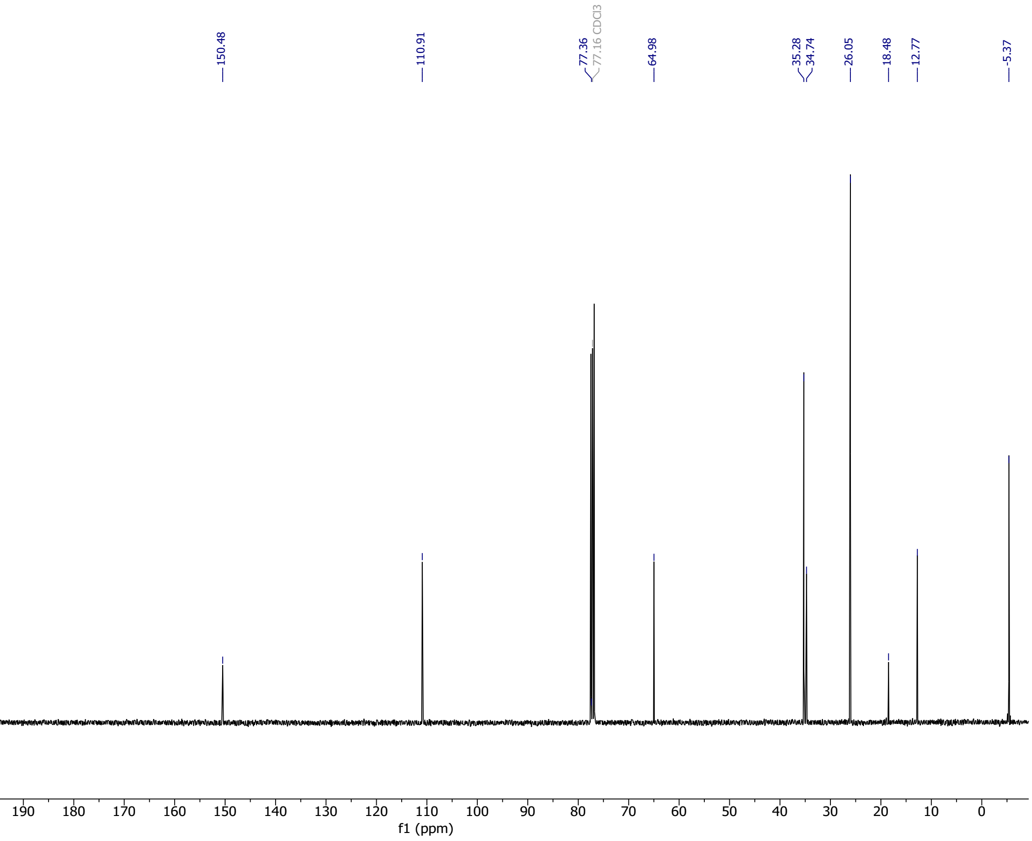

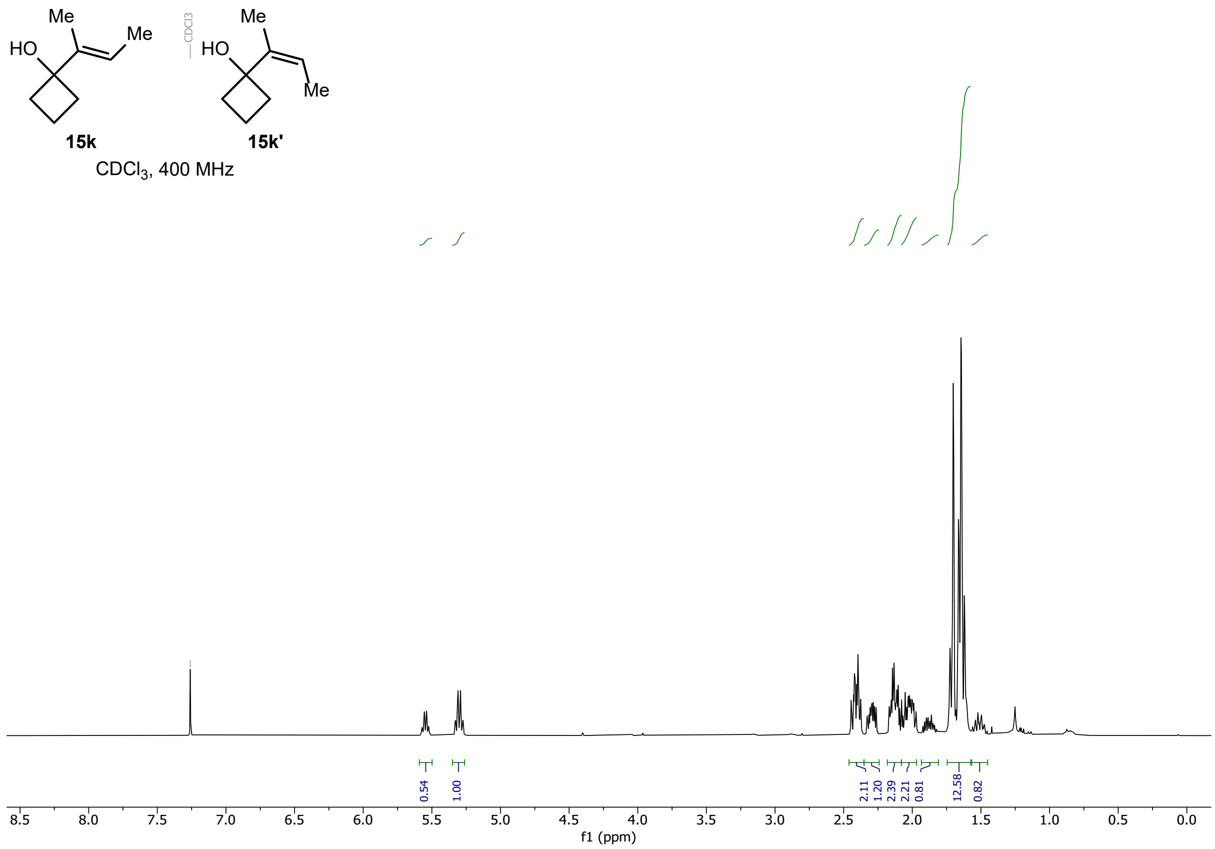
Me
HO
Me
15k
15k'
CDCl3, 400 MHz
0.54
1.00
2.11
1.20
2.39
2.21
0.81
12.58
0.82
f1 (ppm)

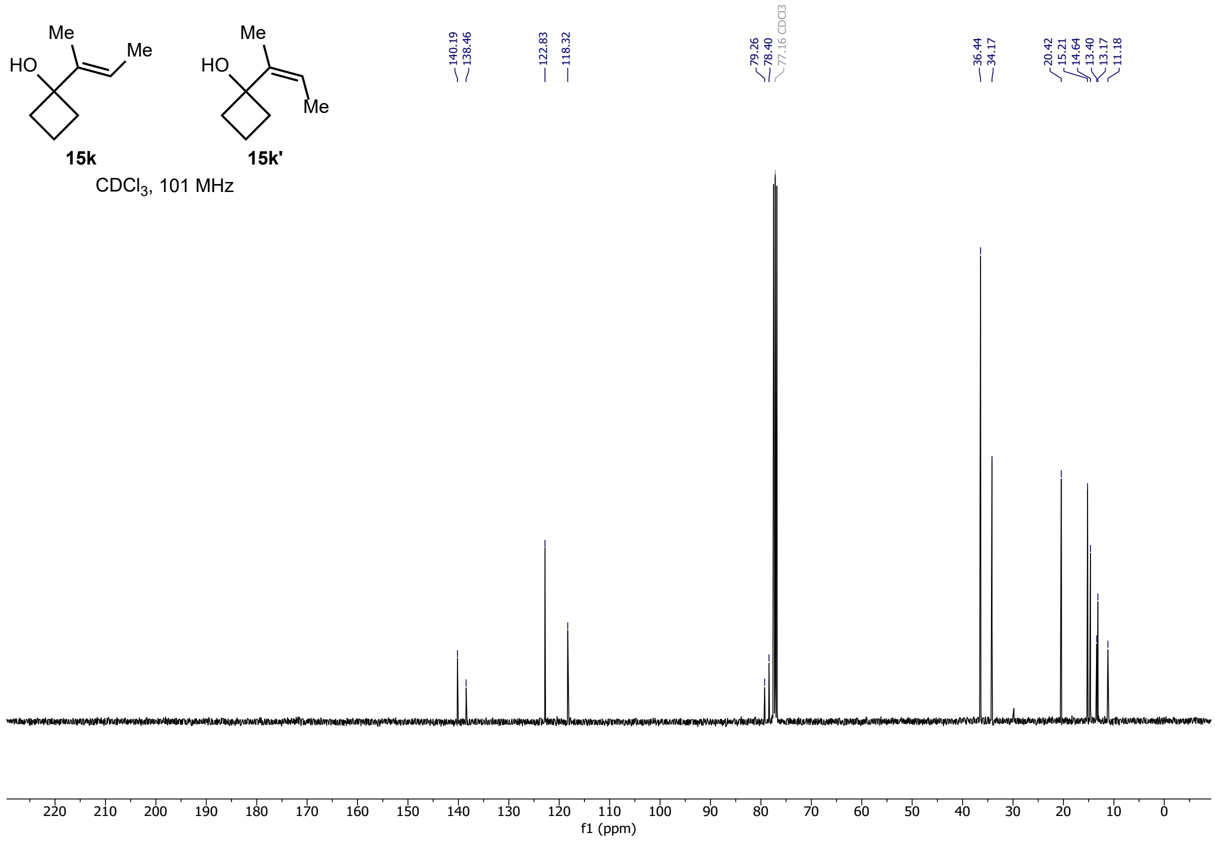
Me
HO
Me
15k
15k'
CDCl3, 101 MHz
140.19
138.46
122.83
118.32
79.26
78.40
77.16 CDCl3
36.44
34.17
20.42
15.21
14.64
13.40
13.17
11.18
f1 (ppm)

9. Copies of ^{1}H, ^{13}C, and ^{19}F NMR Spectra for all Products

Peaks corresponding to a minor rotamer (confirmed by VT NMR spectroscopy studies) can be observed in the ^{1}H NMR spectra of some of the purified products.

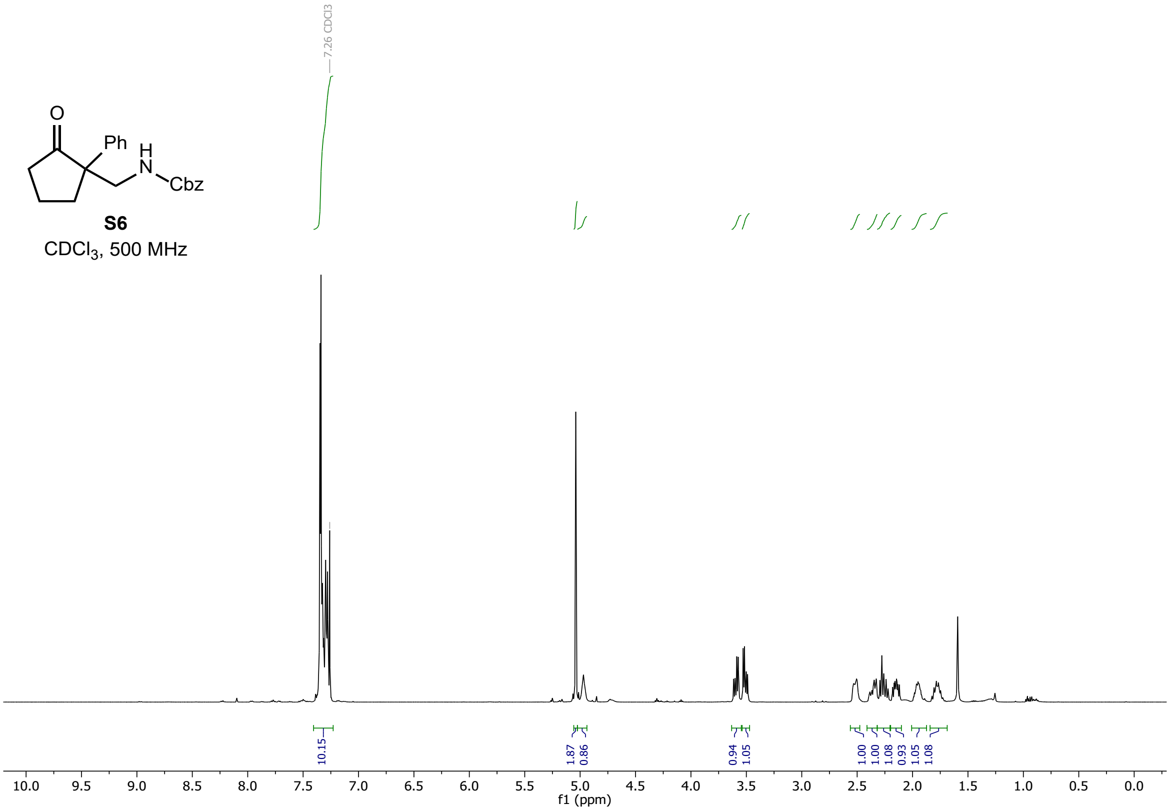

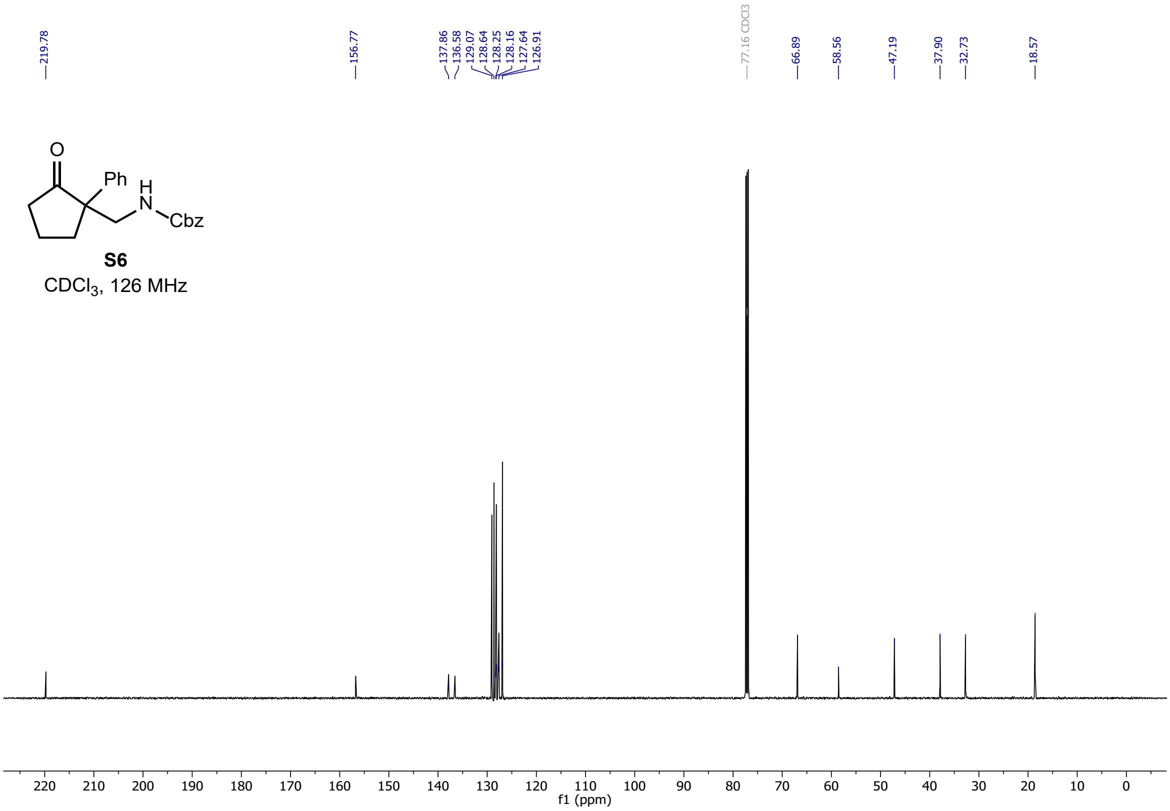

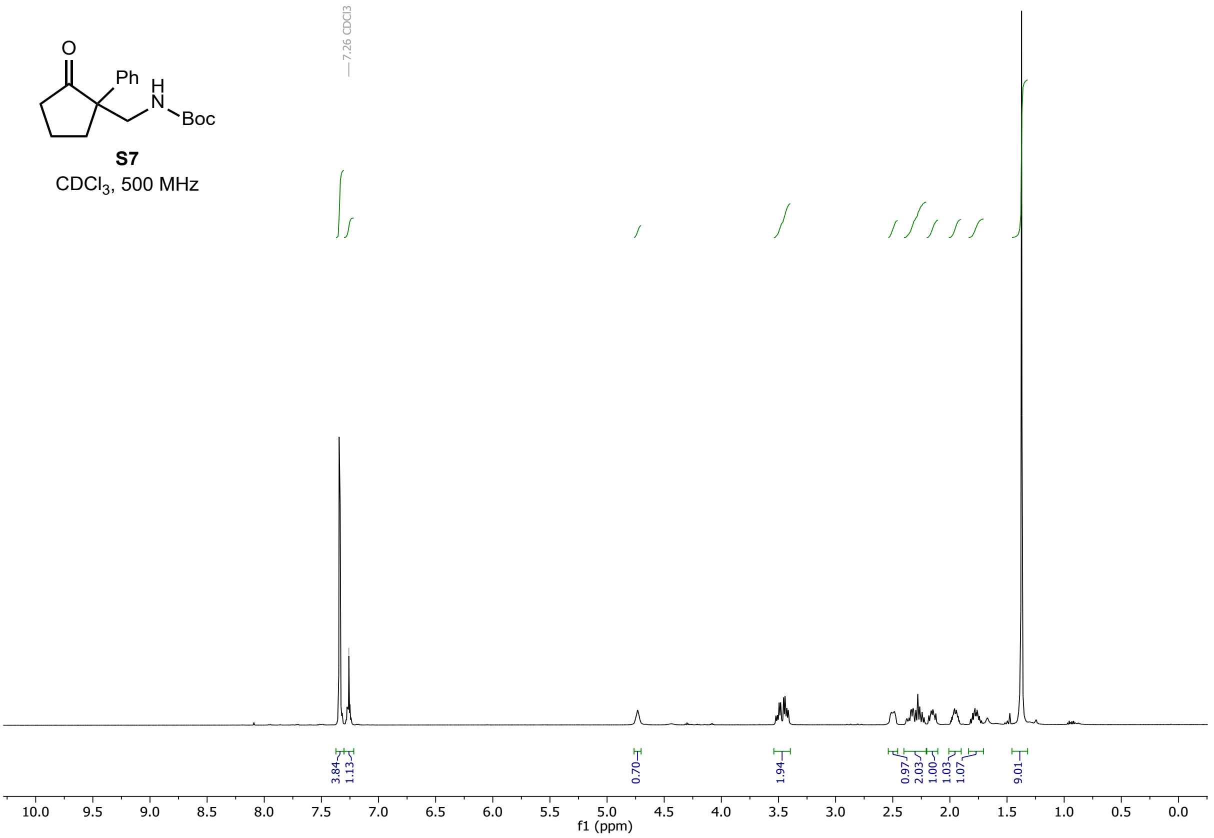
O
Ph
H
N
Boc
S7
CDCl$_3$, 500 MHz
7.26 CDCl3
3.84
1.13
0.70
1.94
0.97
2.03
1.00
1.03
1.07
9.01
10.0 9.5 9.0 8.5 8.0 7.5 7.0 6.5 6.0 5.5 5.0 4.5 4.0 3.5 3.0 2.5 2.0 1.5 1.0 0.5 0.0
f1 (ppm)

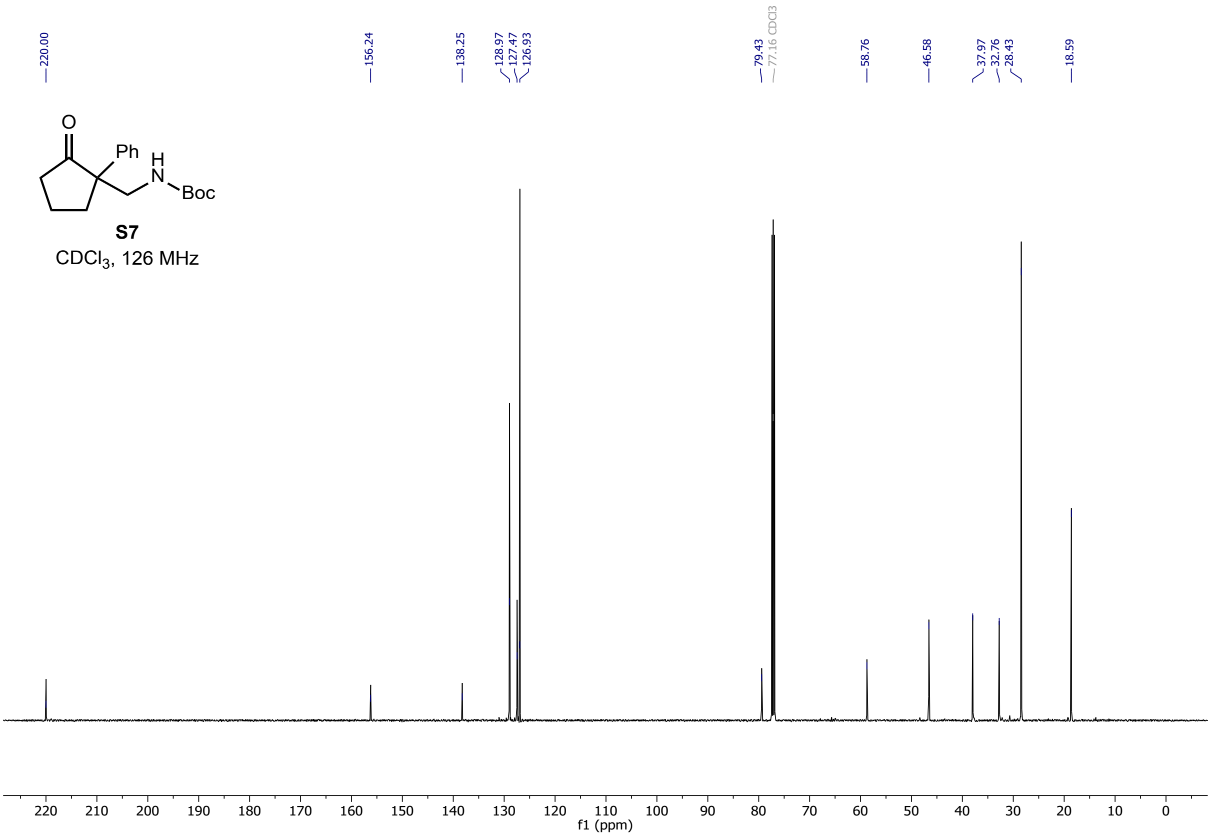
220.00
156.24
138.25
128.97
127.47
126.93
79.43
77.16 CDCl3
58.76
46.58
37.97
32.76
28.43
18.59
O
Ph
H
N
Boc
S7
CDCl$_3$, 126 MHz
220 210 200 190 180 170 160 150 140 130 120 110 100 90 80 70 60 50 40 30 20 10 0
f1 (ppm)

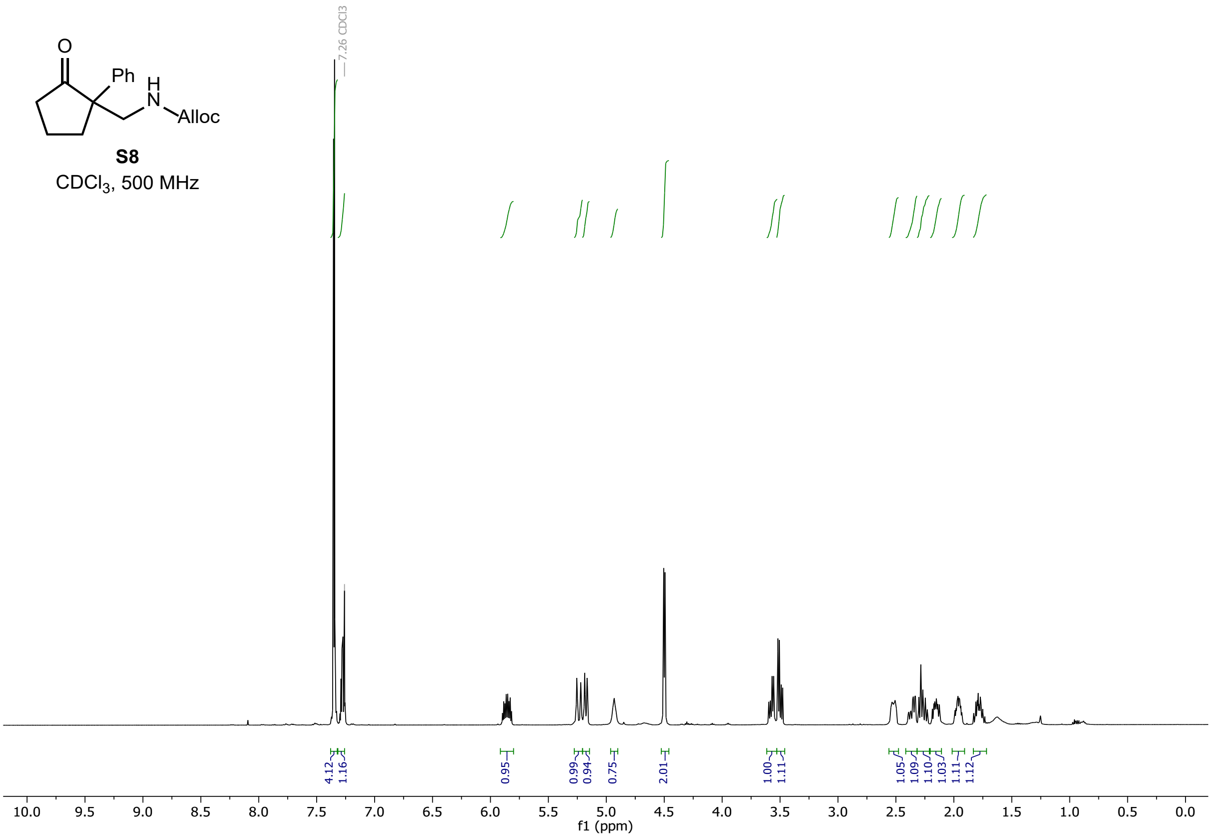

O
Ph
H
N
Alloc
S8
CDCl3, 500 MHz
7.26 CDCl3
4.12
1.16
0.95
0.99
0.94
0.75
2.01
1.00
1.11
1.05
1.09
1.10
1.03
1.11
1.12
10.0 9.5 9.0 8.5 8.0 7.5 7.0 6.5 6.0 5.5 5.0 4.5 4.0 3.5 3.0 2.5 2.0 1.5 1.0 0.5 0.0
f1 (ppm)

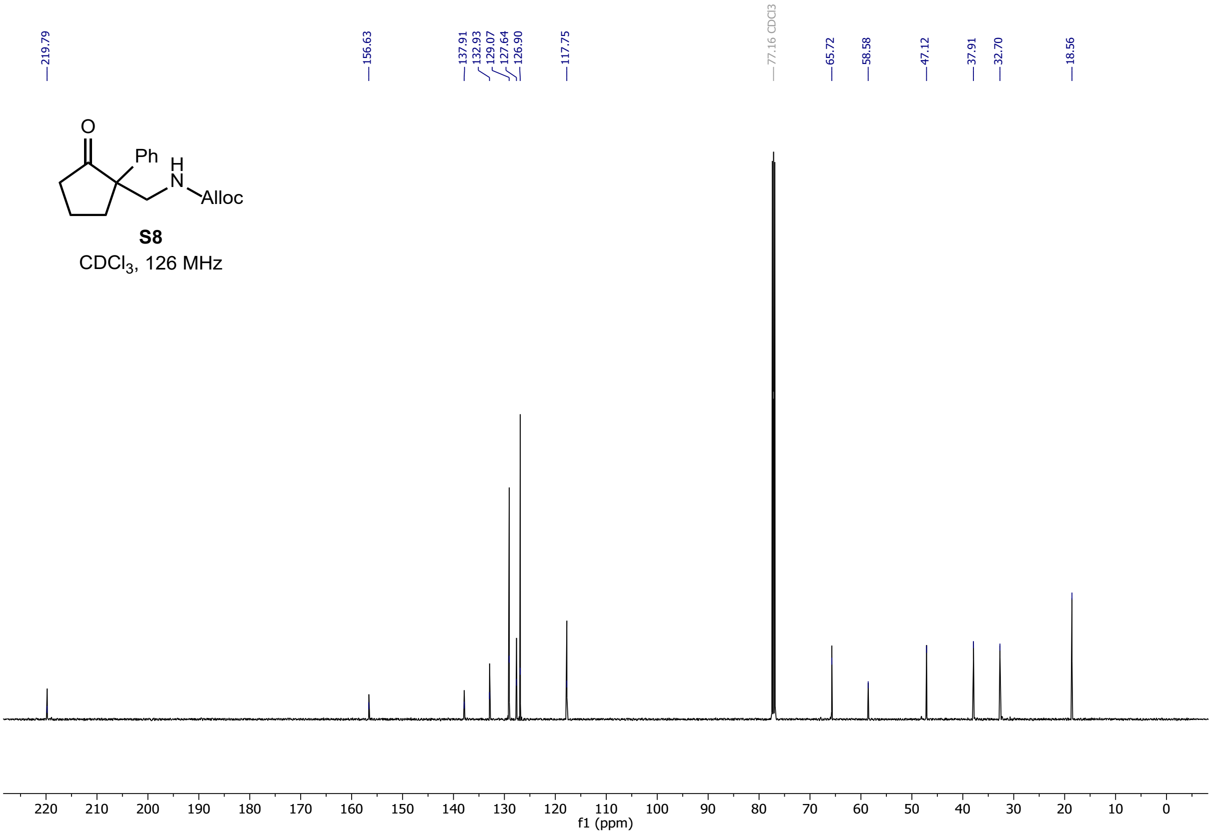

219.79
156.63
137.91
132.93
129.07
127.64
126.90
117.75
77.16 CDCl3
65.72
58.58
47.12
37.91
32.70
18.56
O
Ph
H
N
Alloc
S8
CDCl3, 126 MHz
220 210 200 190 180 170 160 150 140 130 120 110 100 90 80 70 60 50 40 30 20 10 0
f1 (ppm)

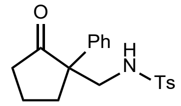

S9

$CDCl_3$, 400 MHz

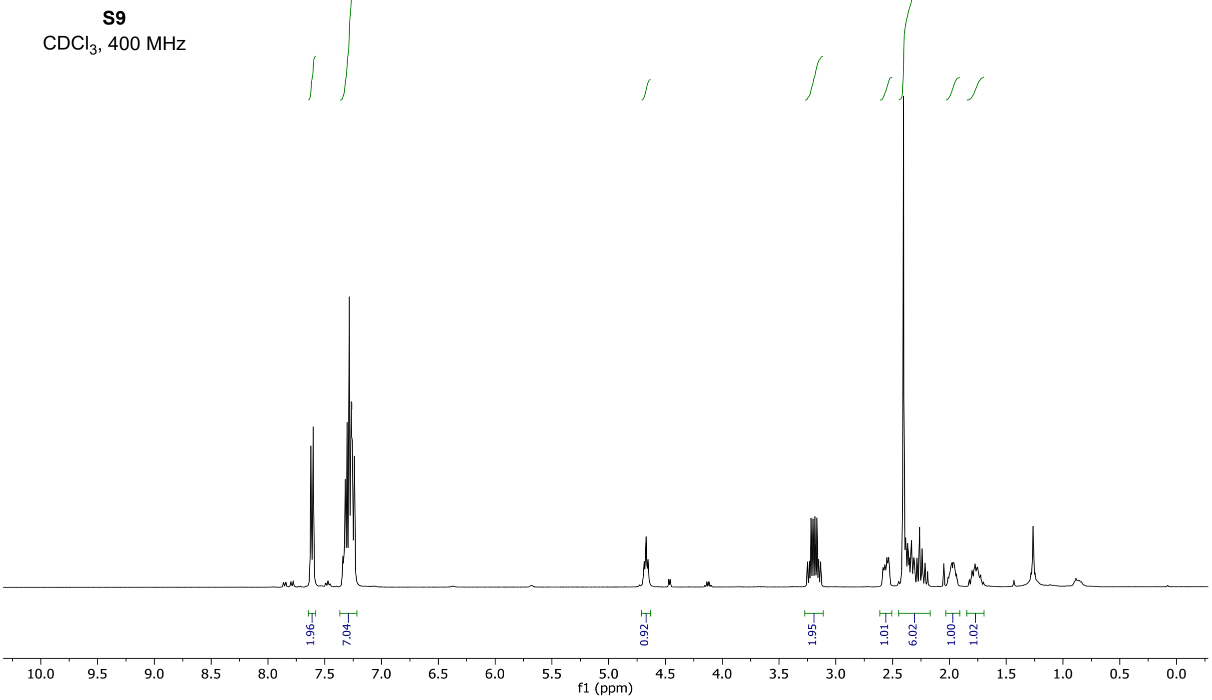

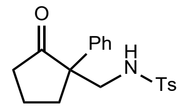

S9

$CDCl_3$, 101 MHz

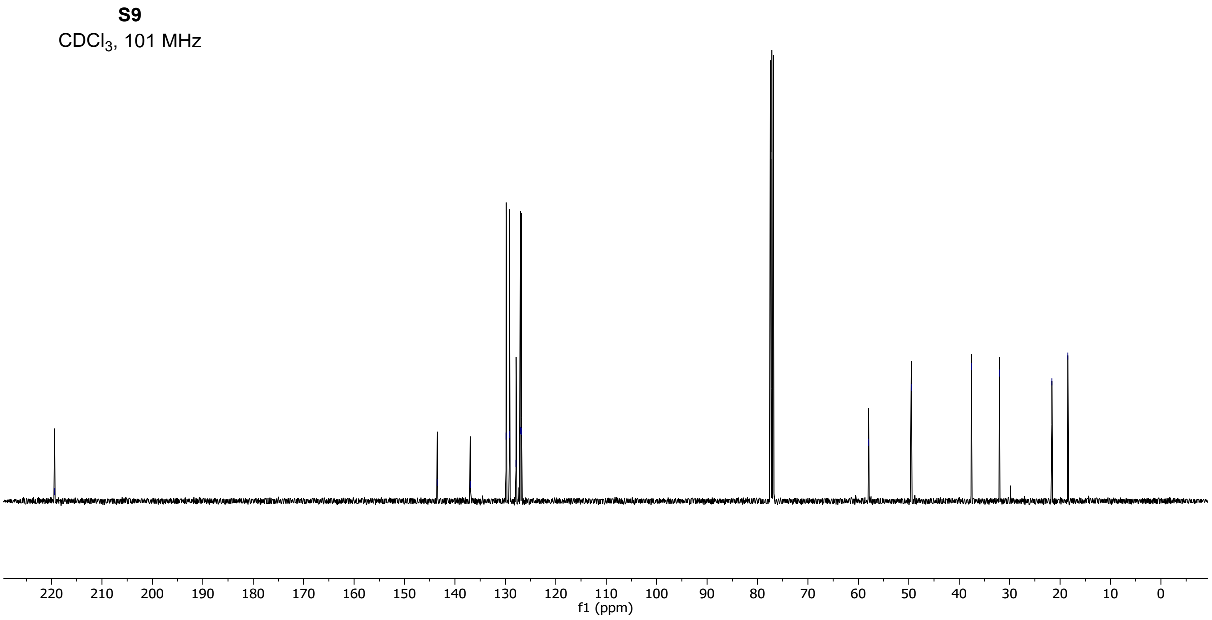

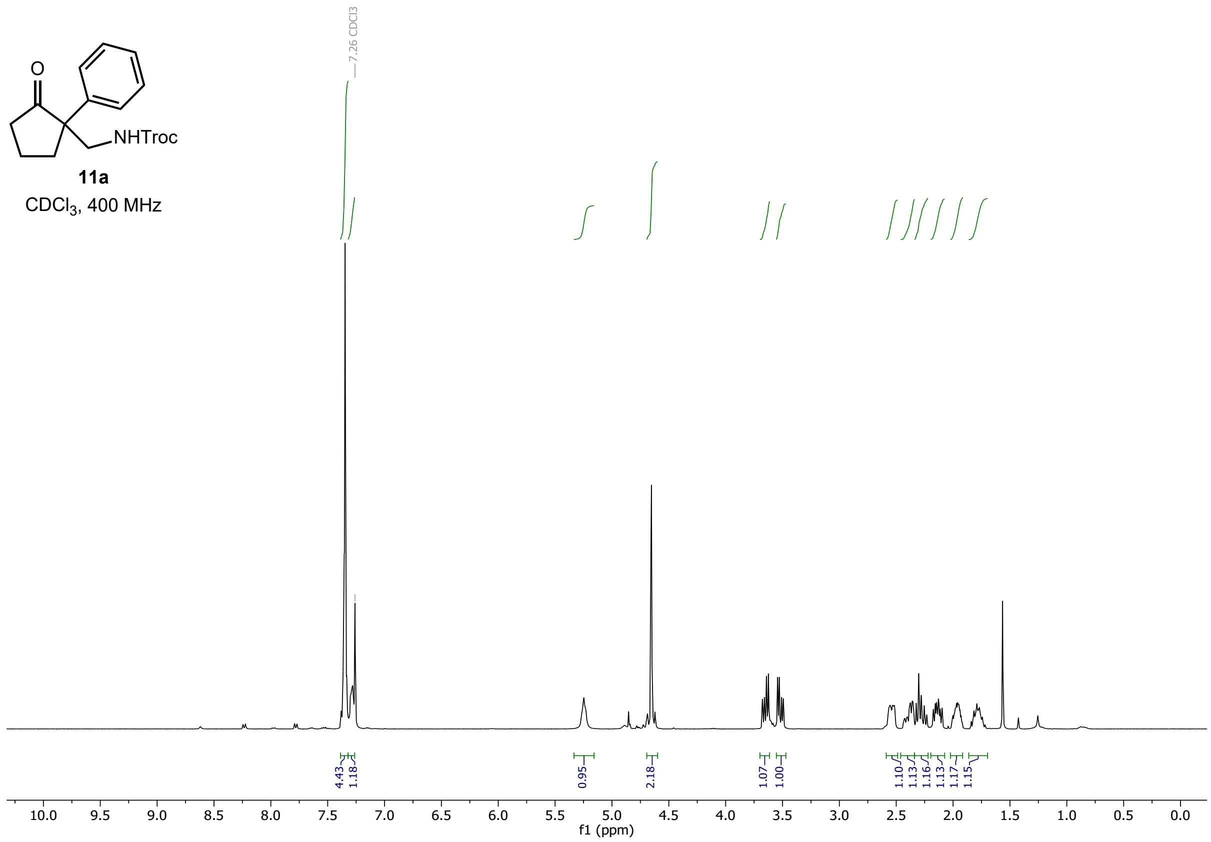
11a
CDCl$_3$, 400 MHz
NHTroc
7.26 CDCl3
4.43
1.18
0.95
2.18
1.07
1.00
1.10
1.13
1.16
1.13
1.17
1.15
f1 (ppm)

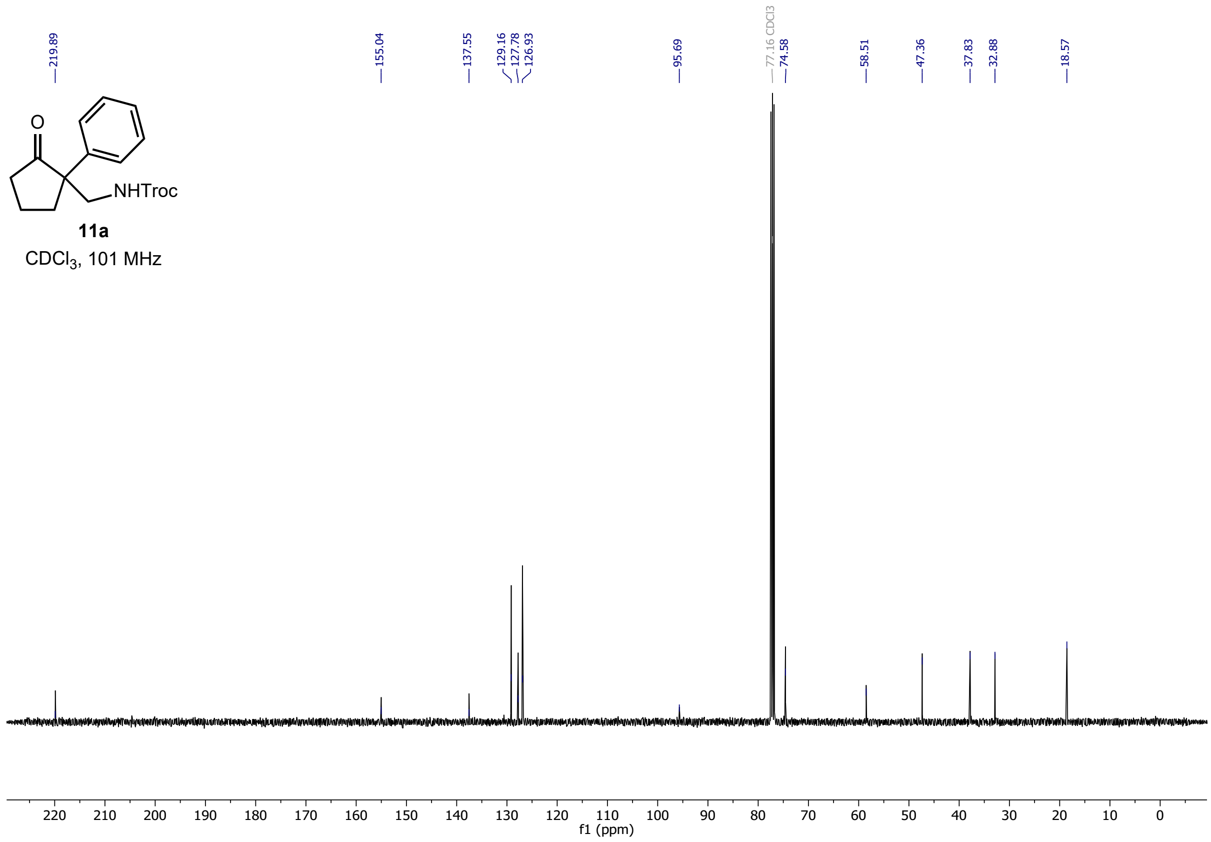
11a
CDCl$_3$, 101 MHz
NHTroc
219.89
155.04
137.55
129.16
127.78
126.93
95.69
77.16 CDCl3
74.58
58.51
47.36
37.83
32.88
18.57
f1 (ppm)

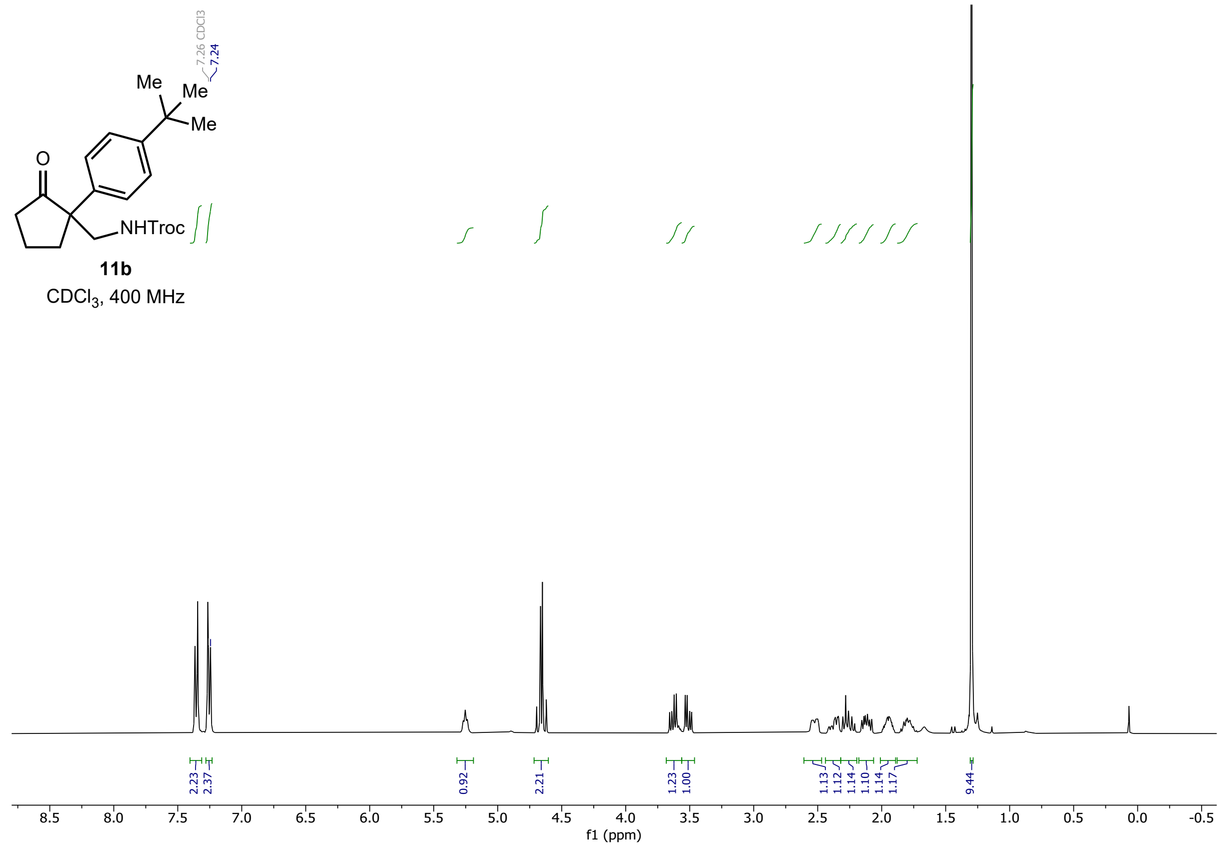

11b

$CDCl_3$, 400 MHz

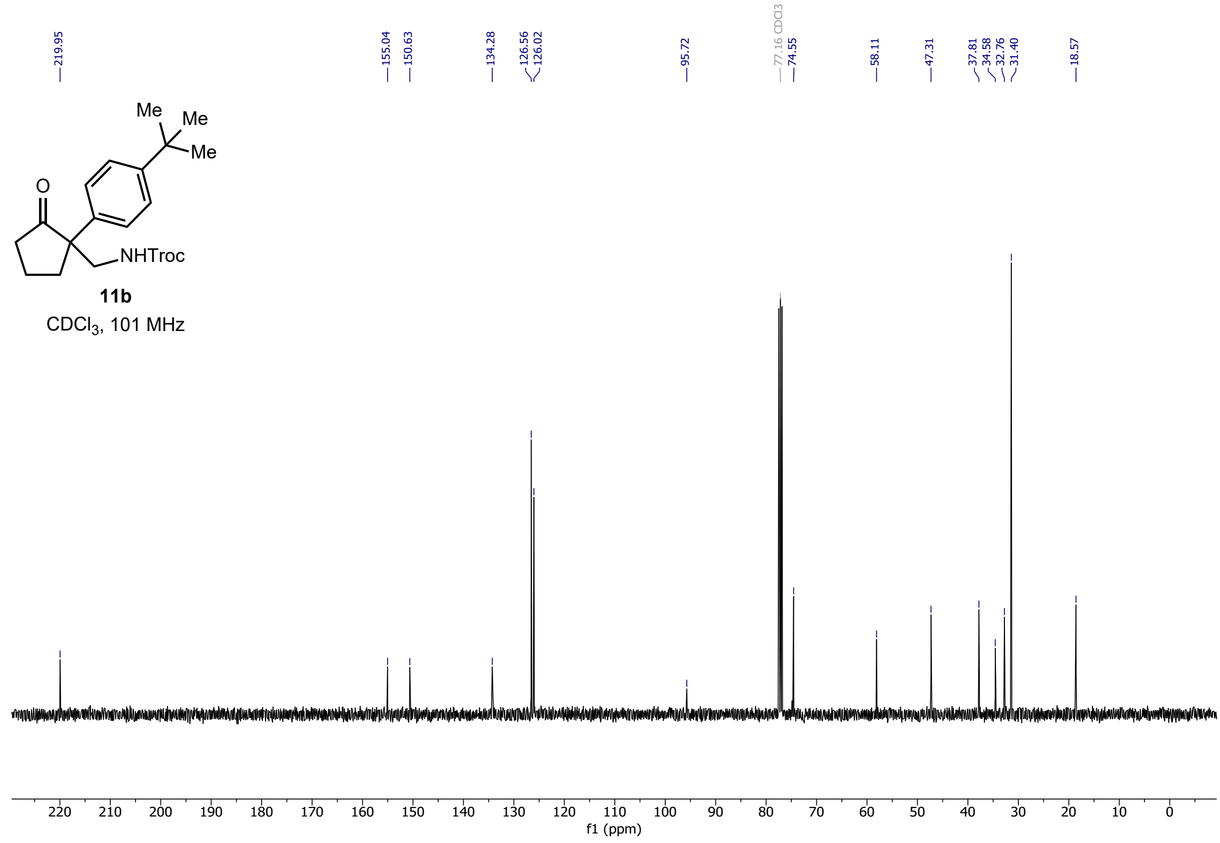

11b

$CDCl_3$, 101 MHz

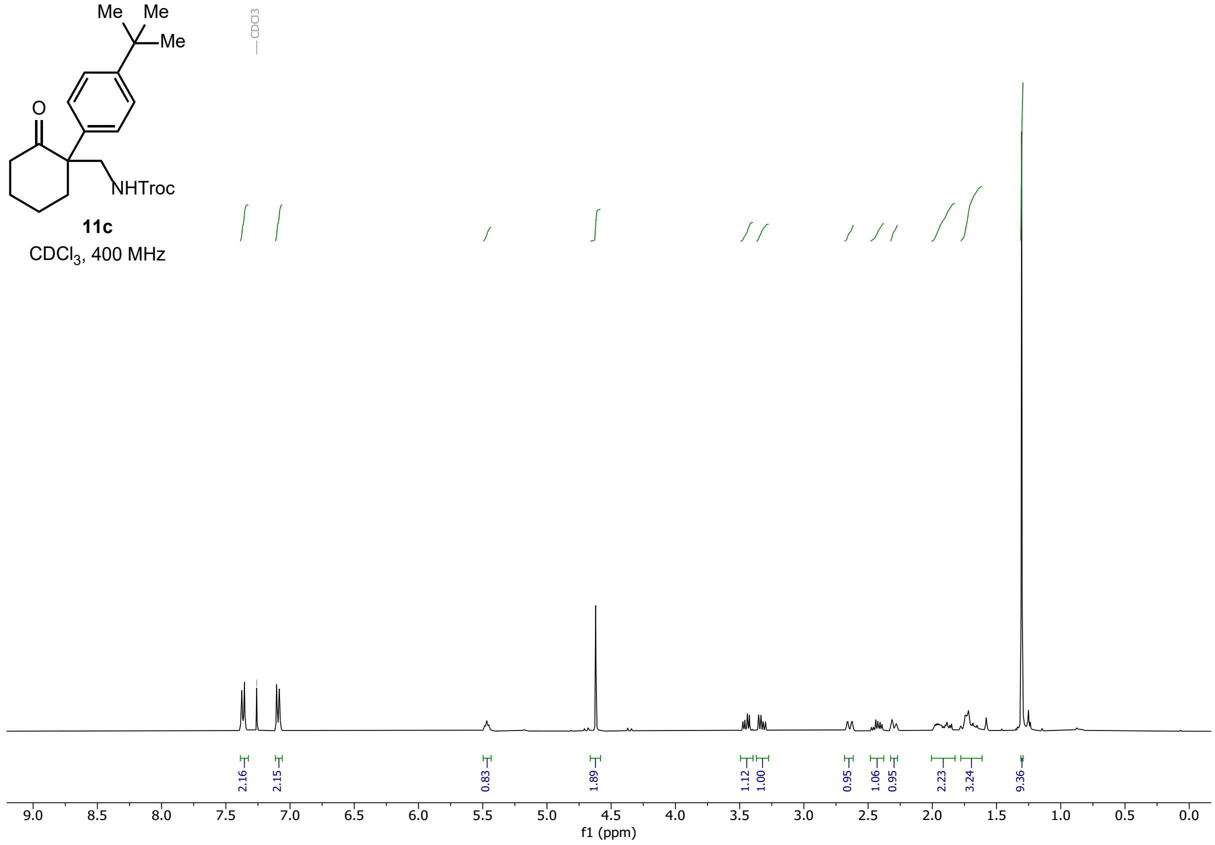
Me Me
Me
O
NHTroc
11c
CDCl$_3$, 400 MHz
CDCl3
2.16 2.15 0.83 1.89 1.12 1.00 0.95 1.06 0.95 2.23 3.24 9.36
9.0 8.5 8.0 7.5 7.0 6.5 6.0 5.5 5.0 4.5 4.0 3.5 3.0 2.5 2.0 1.5 1.0 0.5 0.0
f1 (ppm)

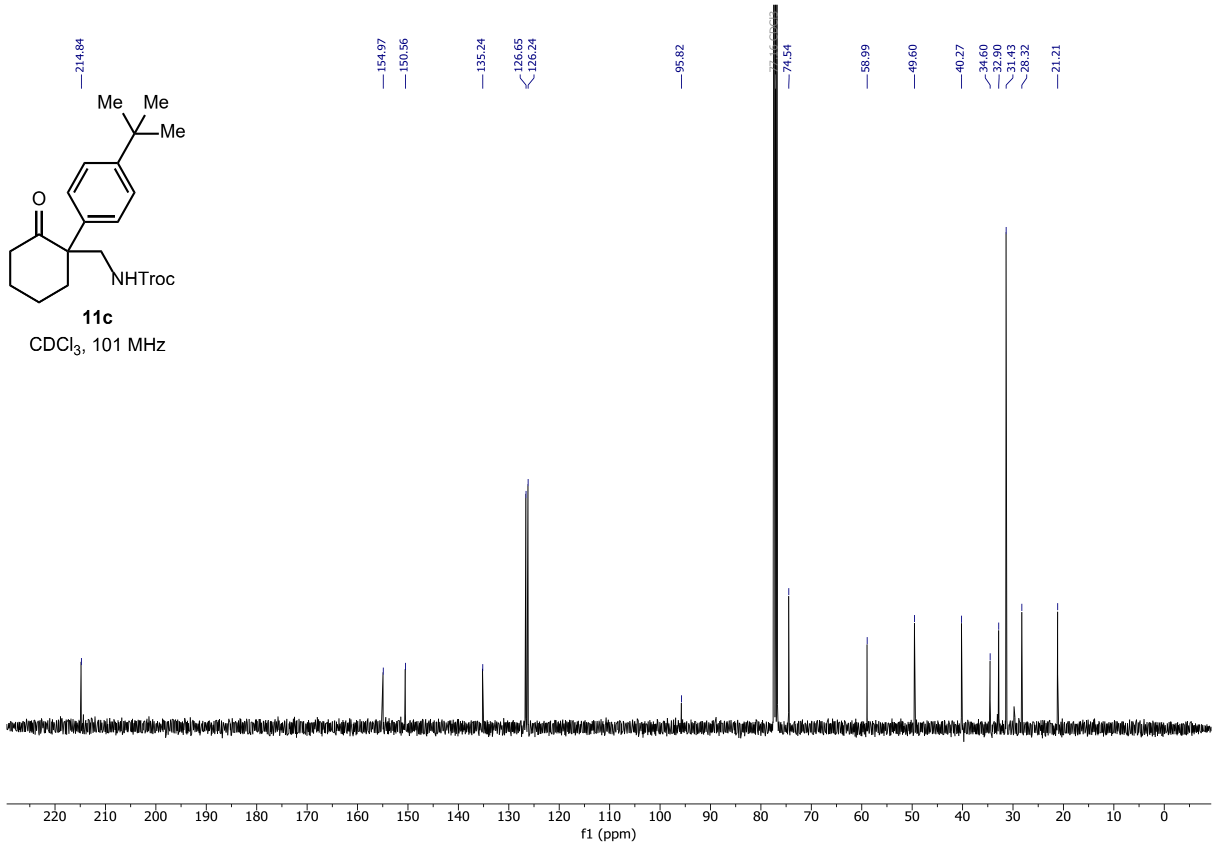
214.84
154.97 150.56
135.24
126.65 126.24
95.82
74.54
58.99
49.60
40.27 34.60 32.90 31.43 28.32
21.21
Me Me
Me
O
NHTroc
11c
CDCl$_3$, 101 MHz
220 210 200 190 180 170 160 150 140 130 120 110 100 90 80 70 60 50 40 30 20 10 0
f1 (ppm)

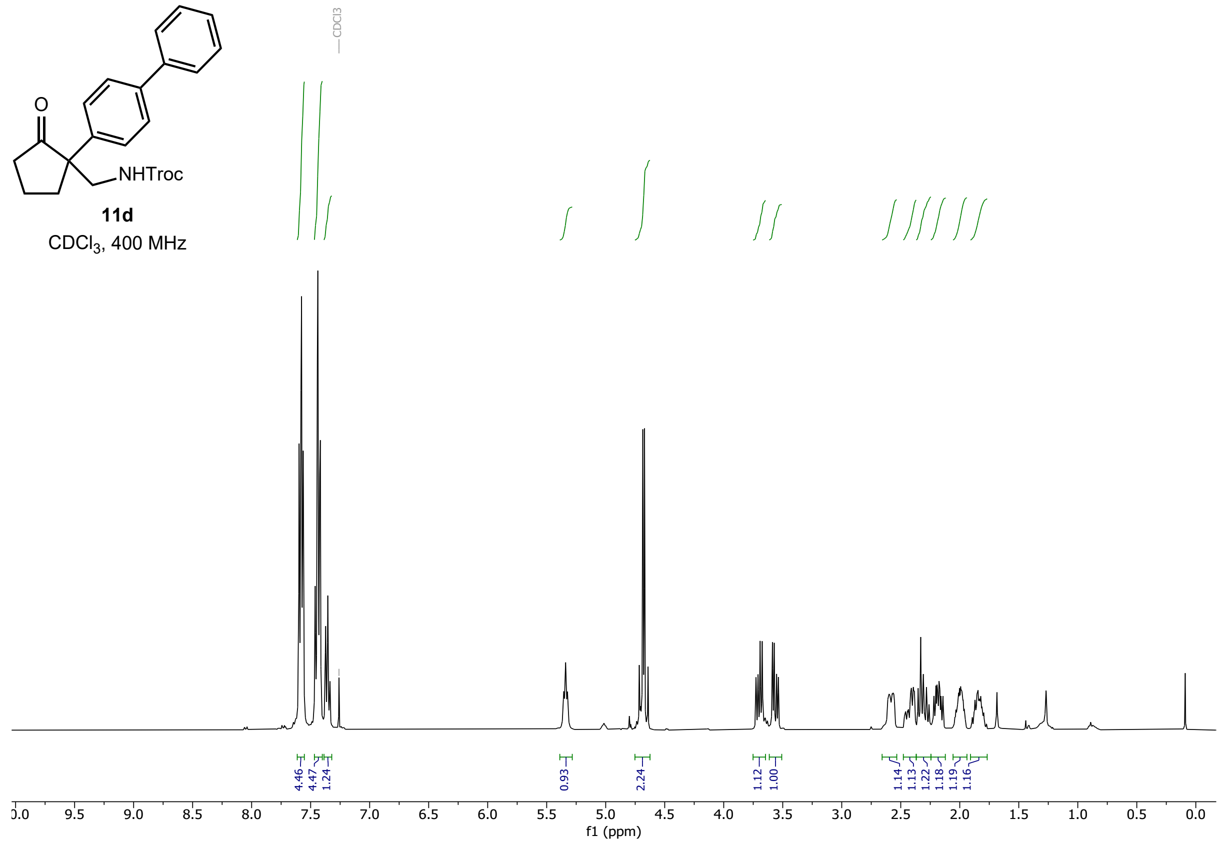
O
NHTroc
11d
CDCl3, 400 MHz
CDCl3
4.46
4.47
1.24
0.93
2.24
1.12
1.00
1.14
1.13
1.22
1.18
1.19
1.16
f1 (ppm)

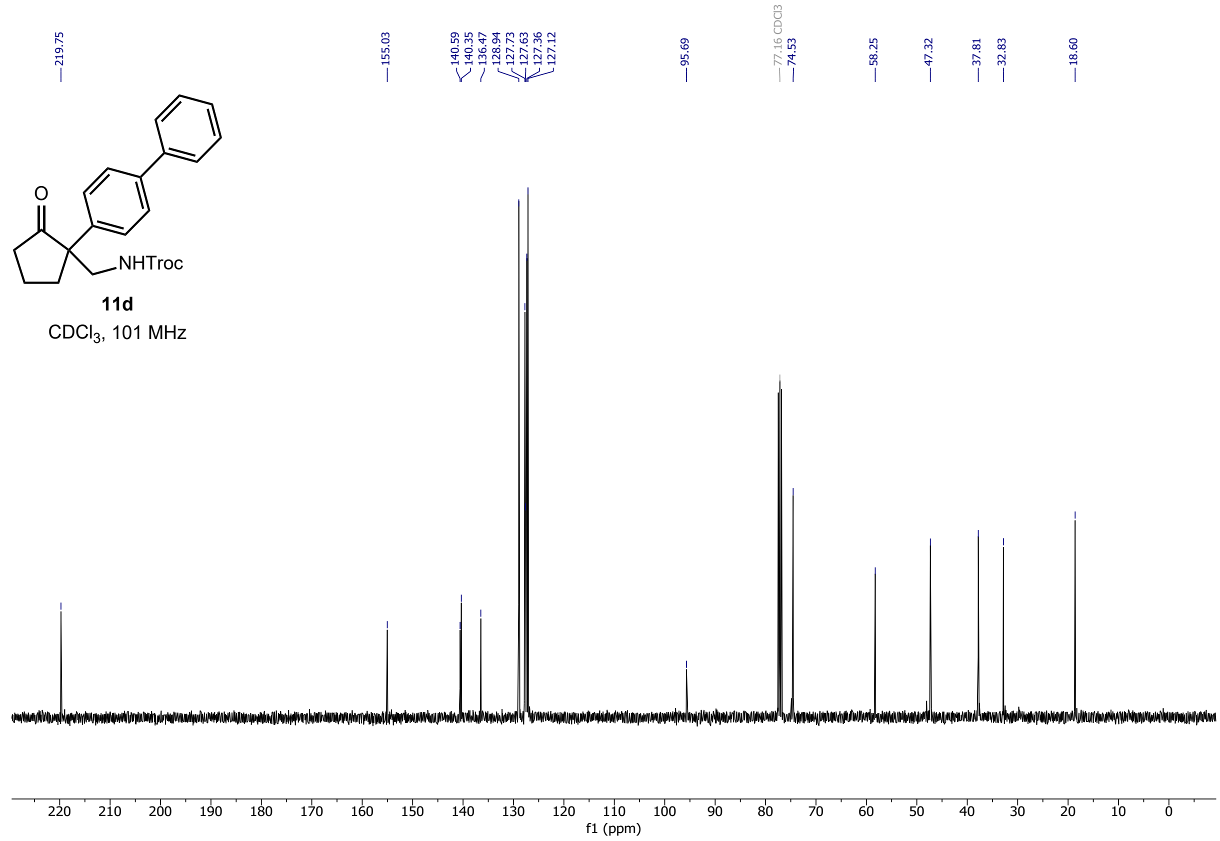
219.75
155.03
140.59
140.35
136.47
128.94
127.73
127.63
127.36
127.12
95.69
77.16 CDCl3
74.53
58.25
47.32
37.81
32.83
18.60
O
NHTroc
11d
CDCl3, 101 MHz
f1 (ppm)

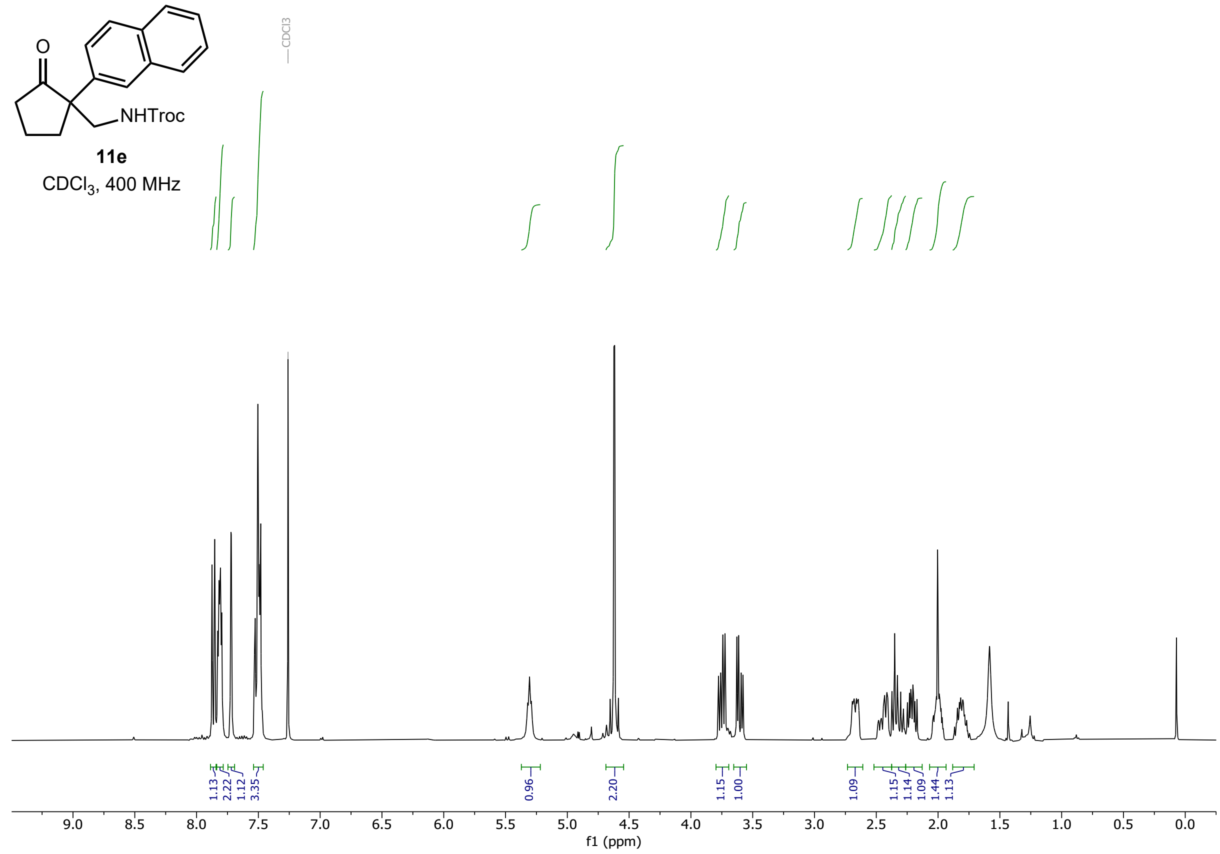

11e
$CDCl_3$, 400 MHz

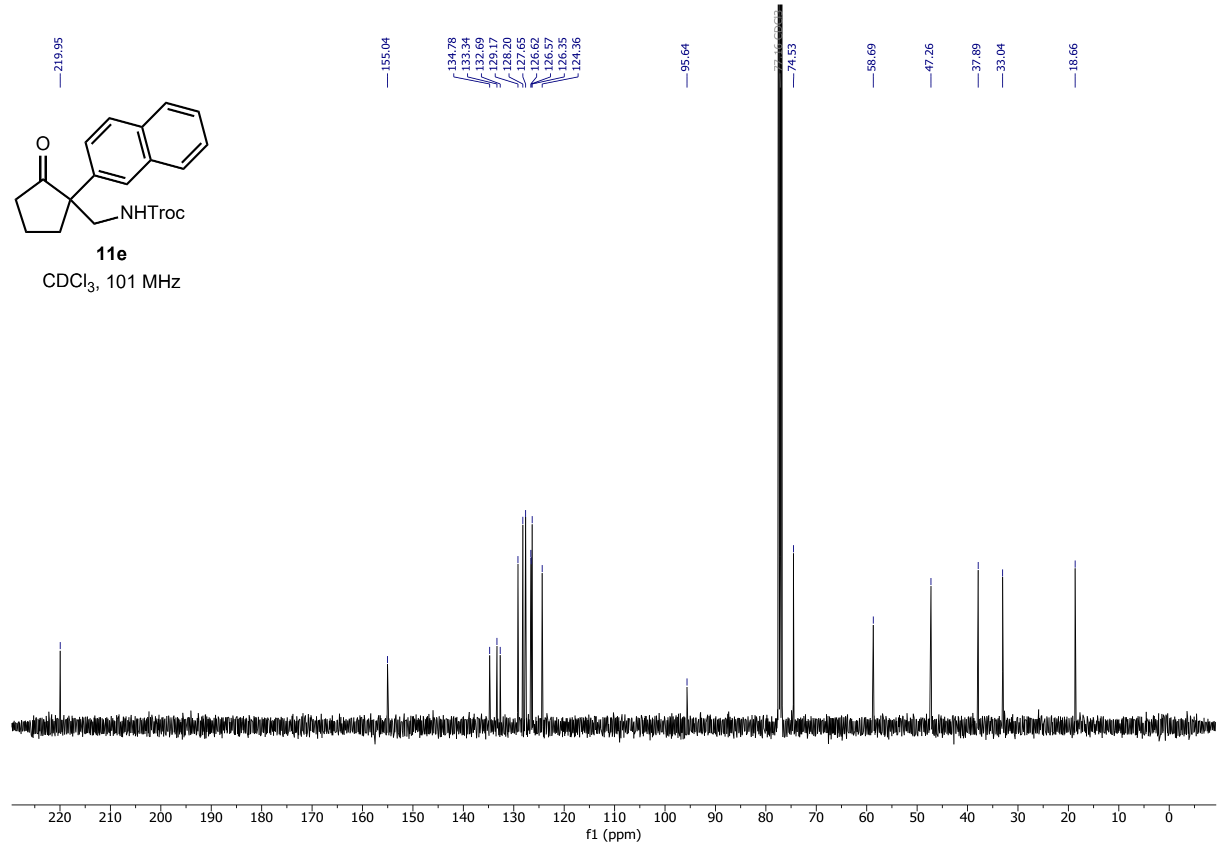

11e
$CDCl_3$, 101 MHz

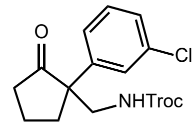

11f

$CDCl_3$, 400 MHz

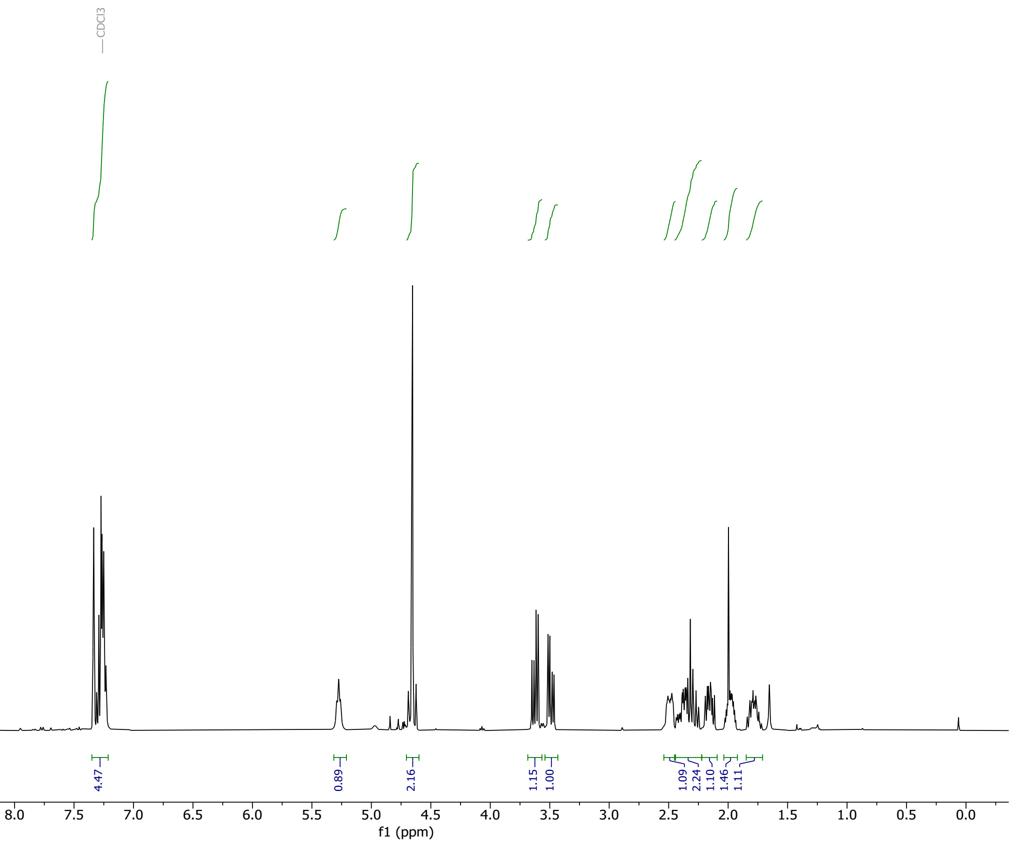

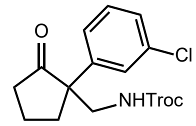

11f

$CDCl_3$, 101 MHz

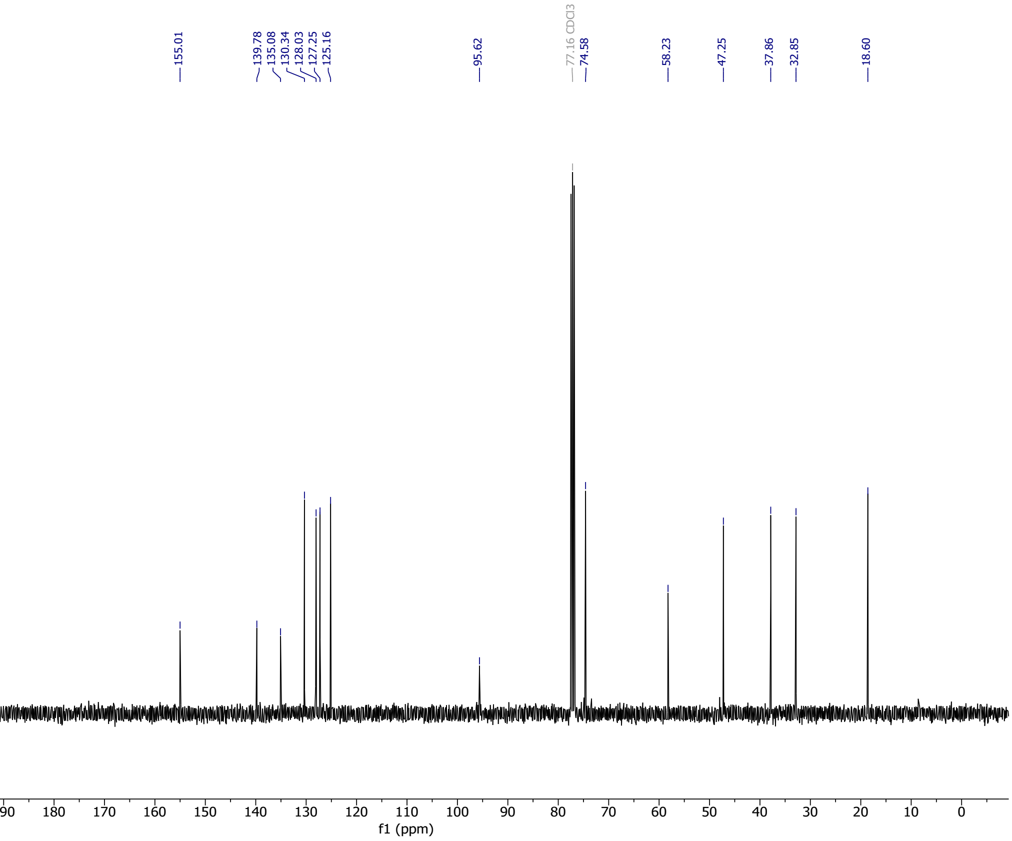

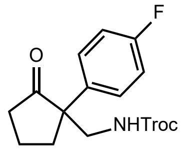

11g

$CDCl_3$, 400 MHz

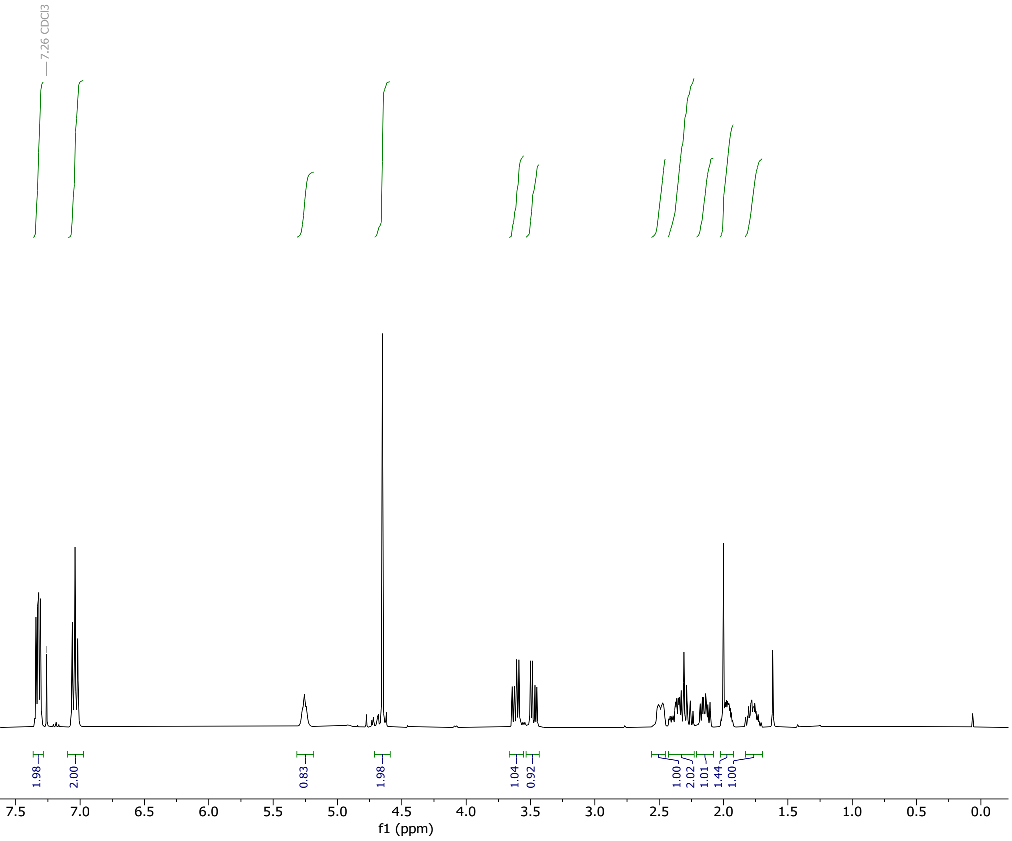

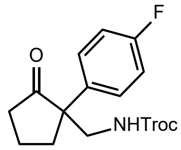

11g

$CDCl_3$, 101 MHz

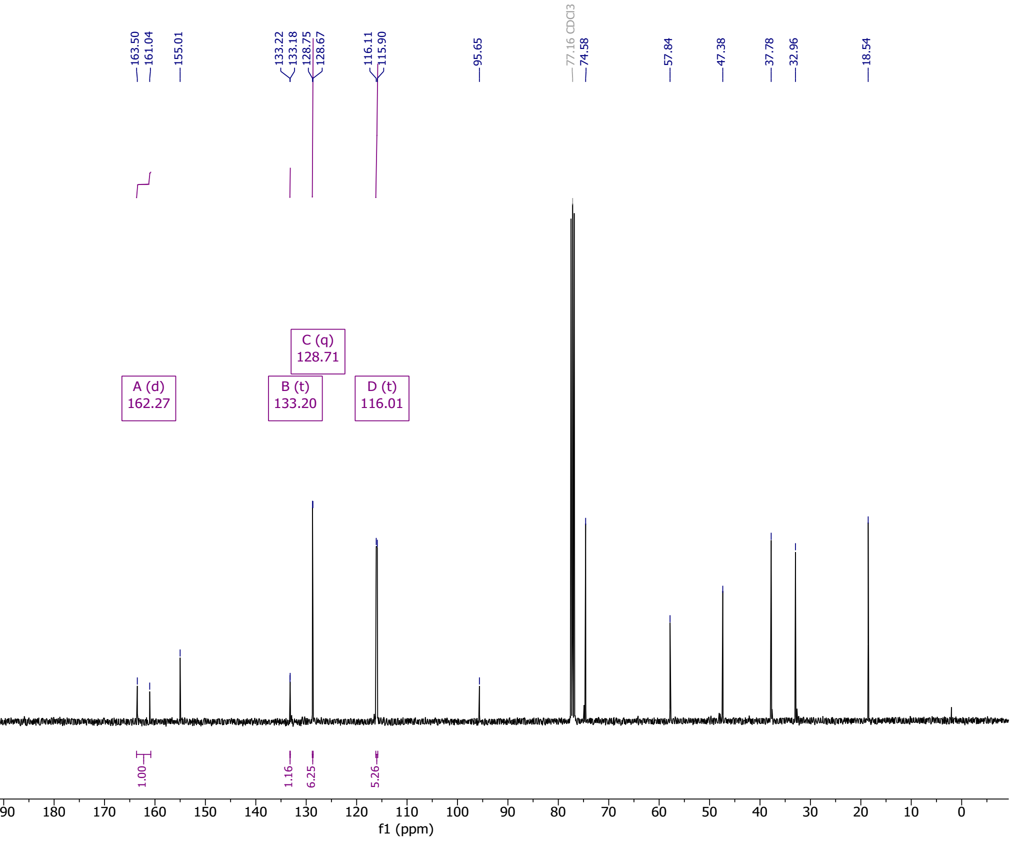

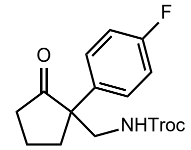

11g

$CDCl_3$, 376 MHz

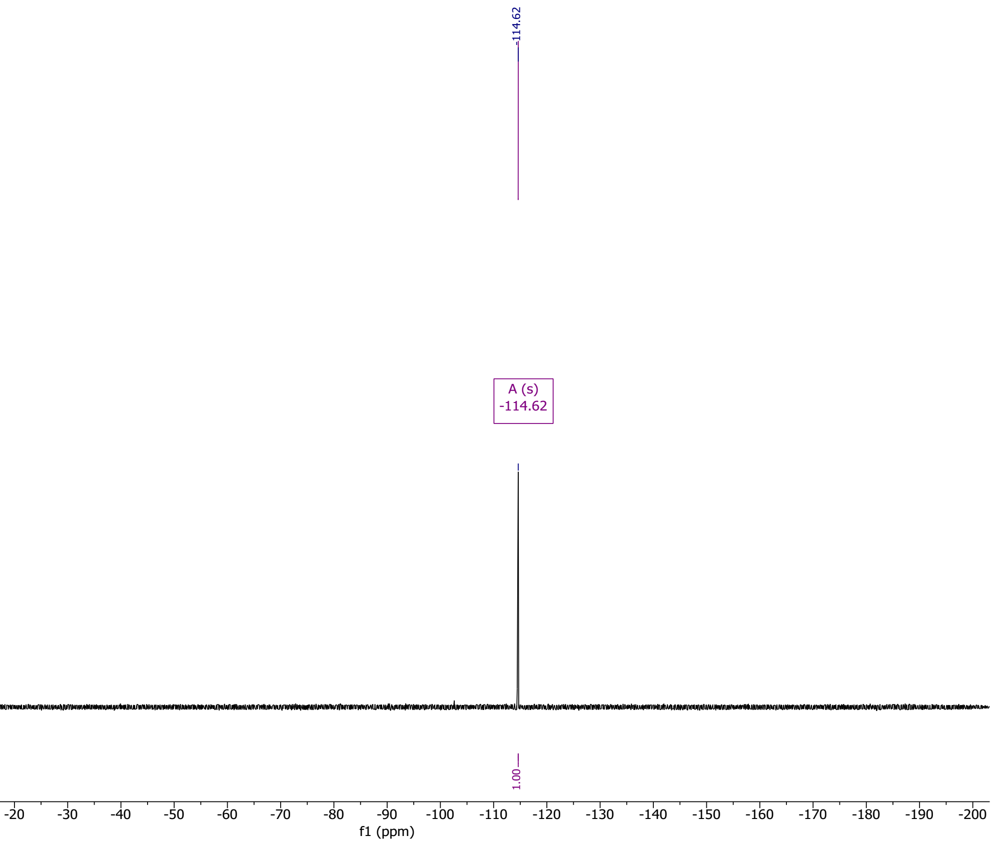

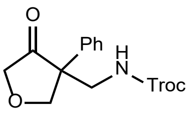

11h

$CDCl_3$, 400 MHz

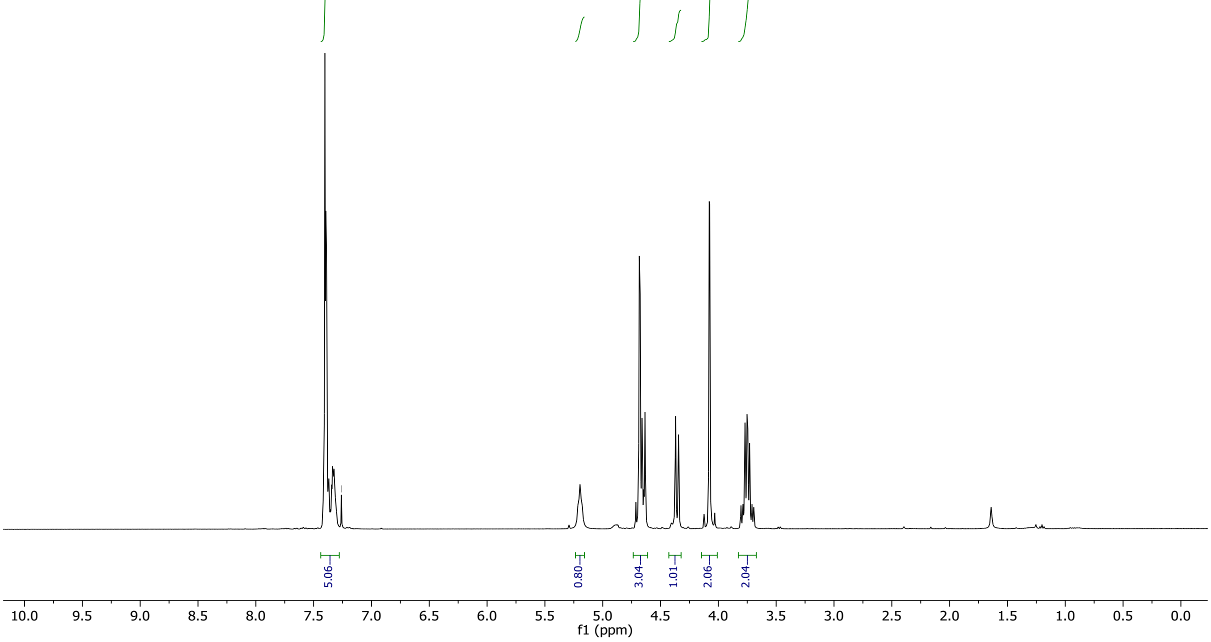

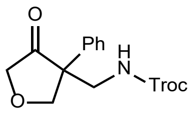

11h

$CDCl_3$, 101 MHz

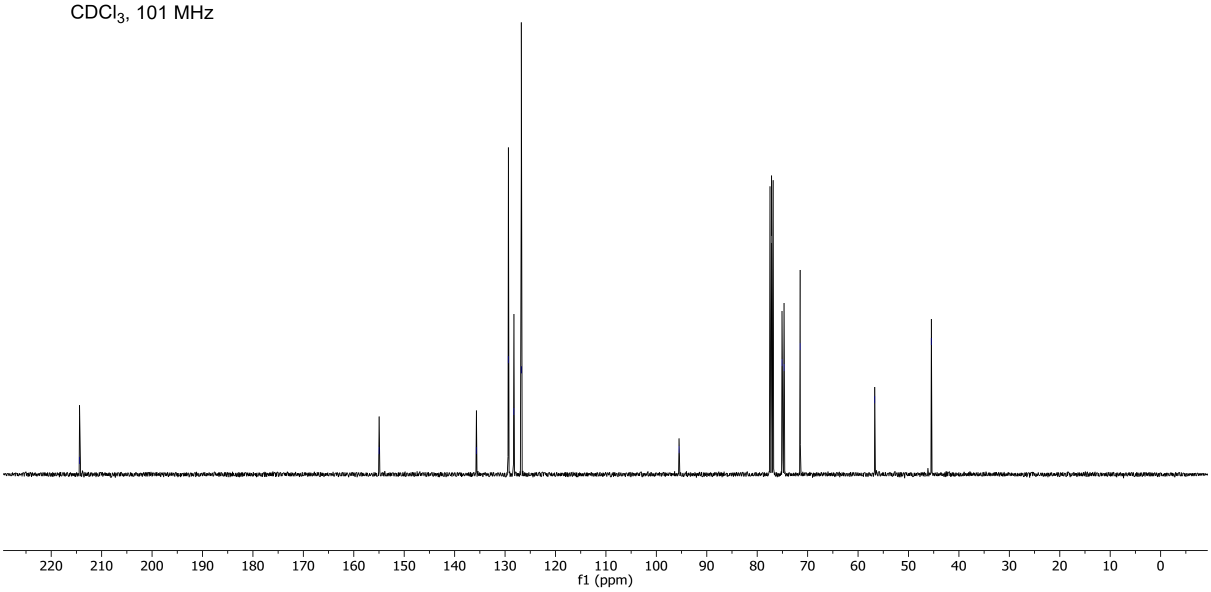

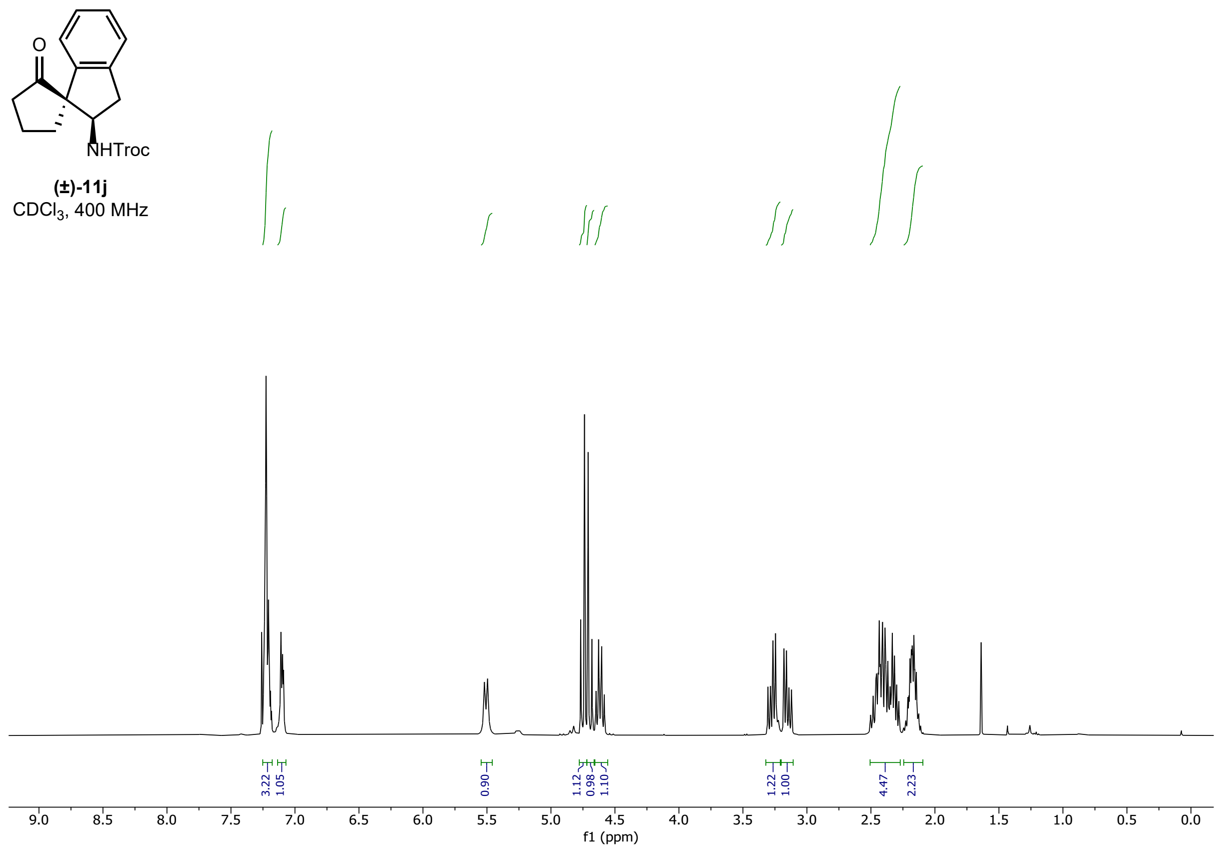

(±)-11j
$CDCl_3$, 400 MHz

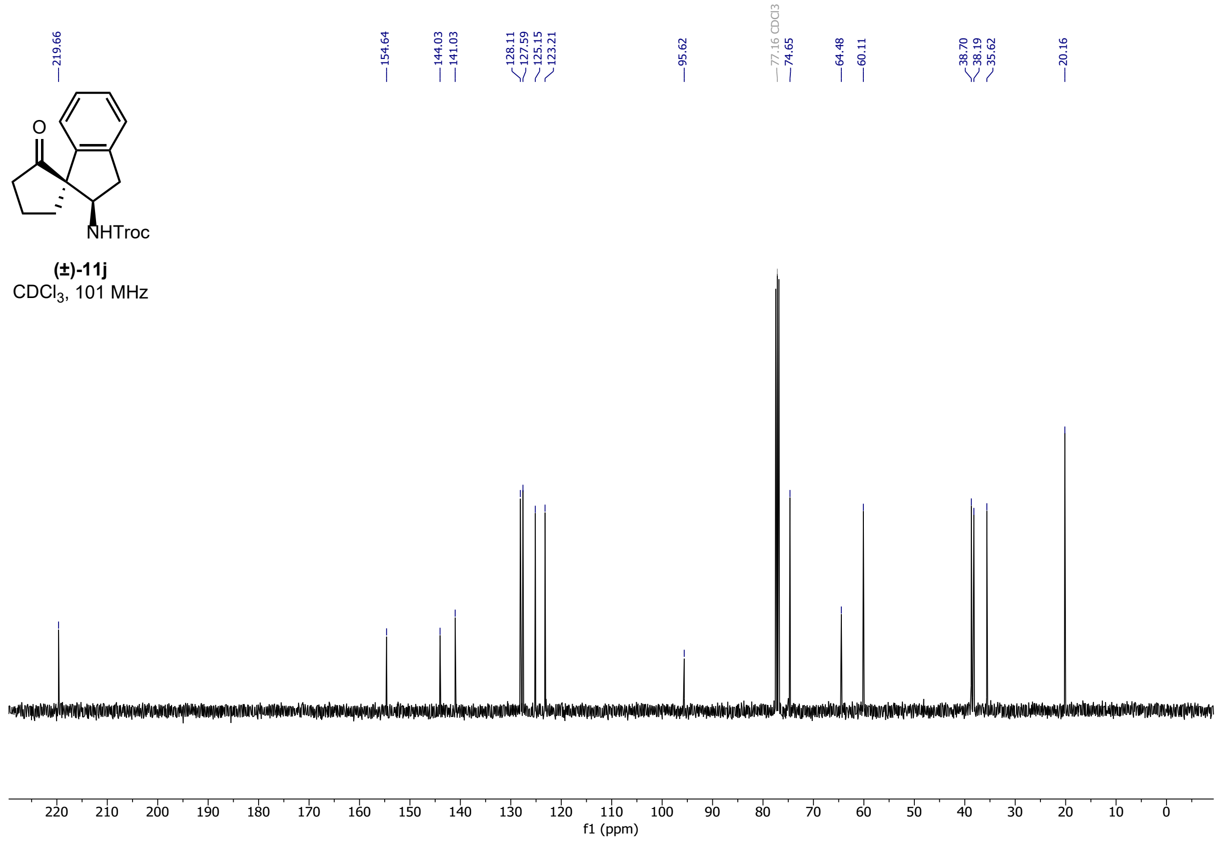

(±)-11j
$CDCl_3$, 101 MHz

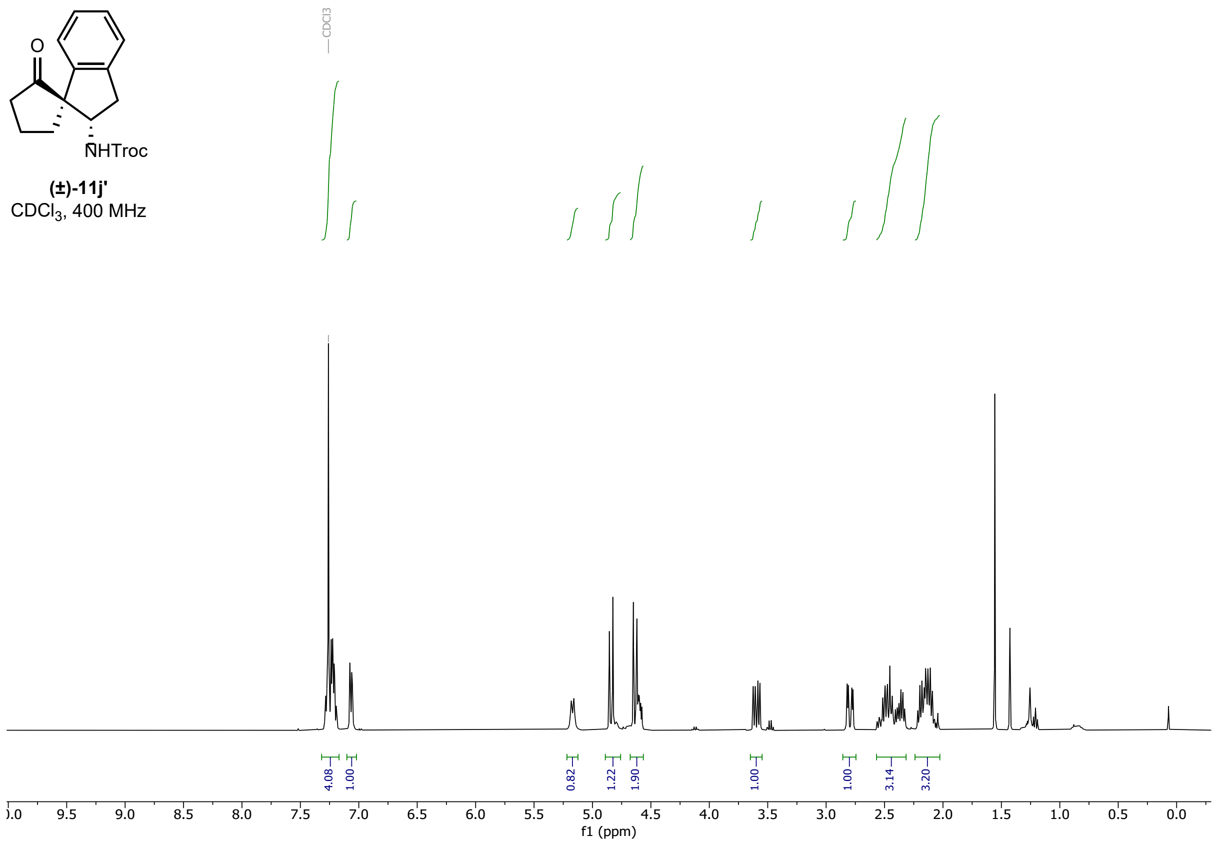
O
NHTroc
(±)-11j'
CDCl3, 400 MHz
CDCl3
4.08
1.00
0.82
1.22
1.90
1.00
1.00
3.14
3.20
f1 (ppm)

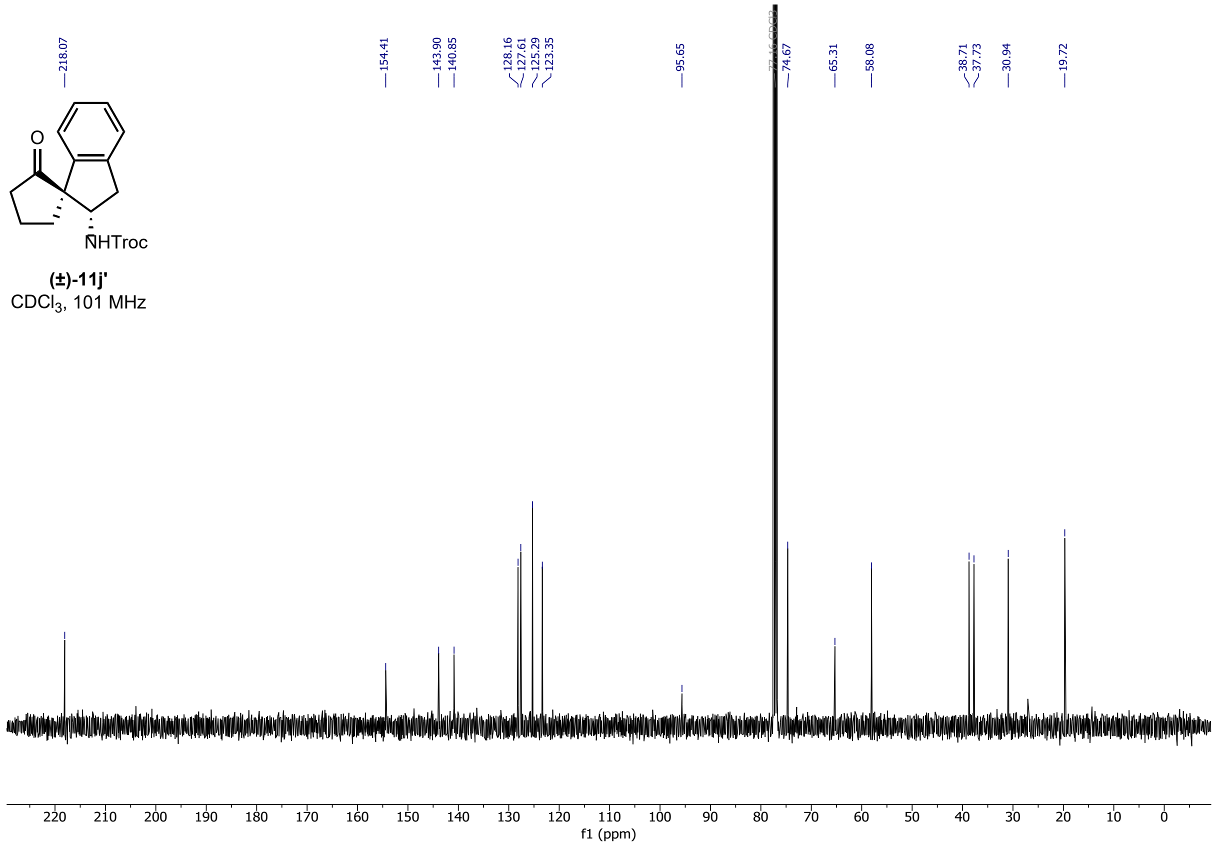
218.07
154.41
143.90
140.85
128.16
127.61
125.29
123.35
95.65
77.16 CDCl3
74.67
65.31
58.08
38.71
37.73
30.94
19.72
O
NHTroc
(±)-11j'
CDCl3, 101 MHz
f1 (ppm)

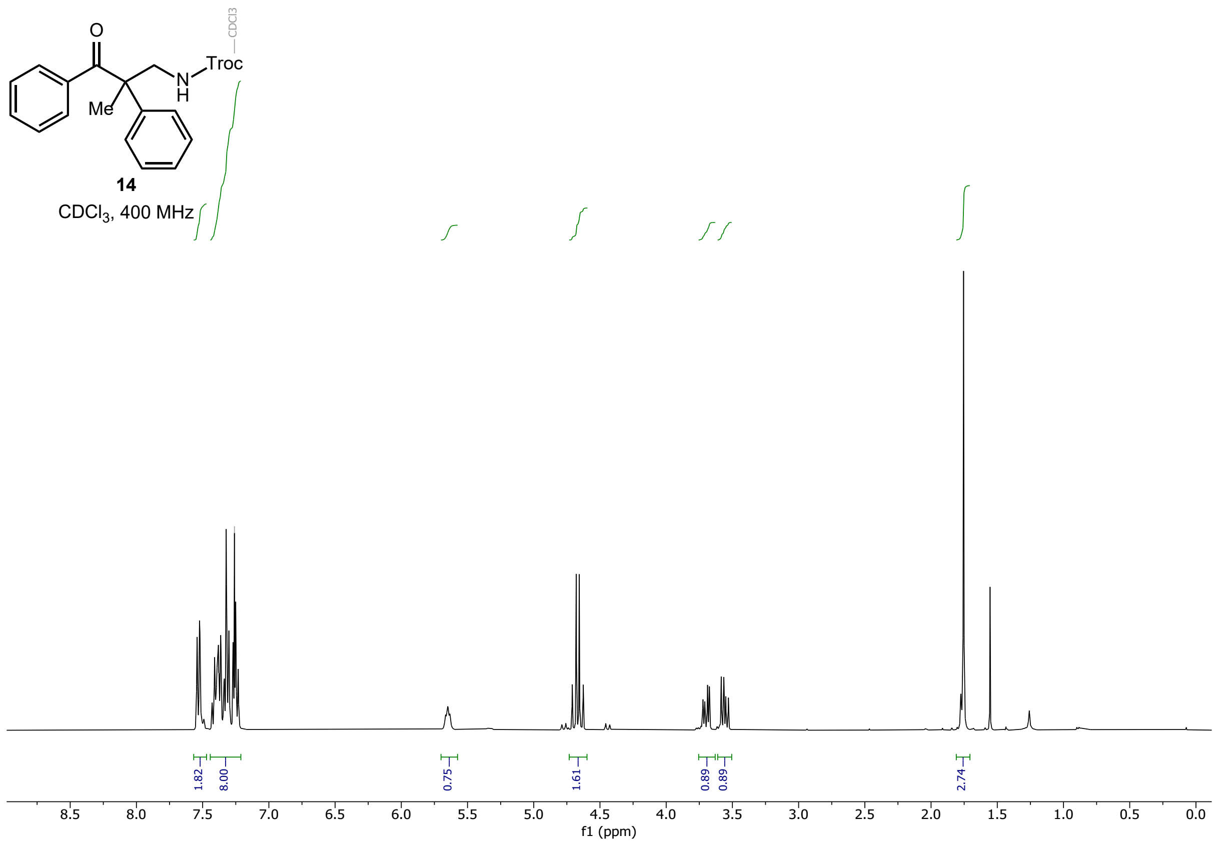
O
N
H
Troc
Me
14
CDCl3, 400 MHz
CDCl3
1.82
8.00
0.75
1.61
0.89
0.89
2.74
8.5 8.0 7.5 7.0 6.5 6.0 5.5 5.0 4.5 4.0 3.5 3.0 2.5 2.0 1.5 1.0 0.5 0.0
f1 (ppm)

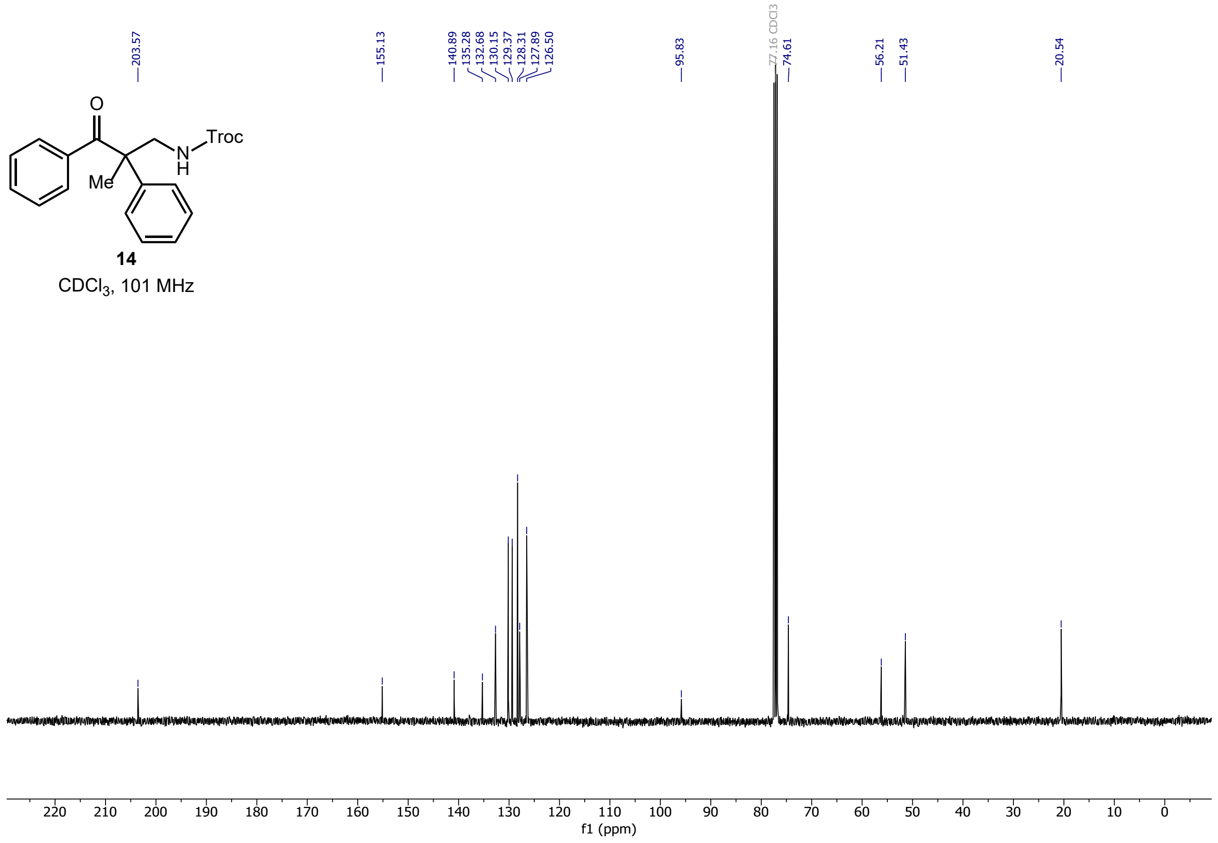
203.57
155.13
140.89
135.28
132.68
130.15
129.37
128.31
127.89
126.50
95.83
77.16 CDCl3
74.61
56.21
51.43
20.54
O
N
H
Troc
Me
14
CDCl3, 101 MHz
220 210 200 190 180 170 160 150 140 130 120 110 100 90 80 70 60 50 40 30 20 10 0
f1 (ppm)

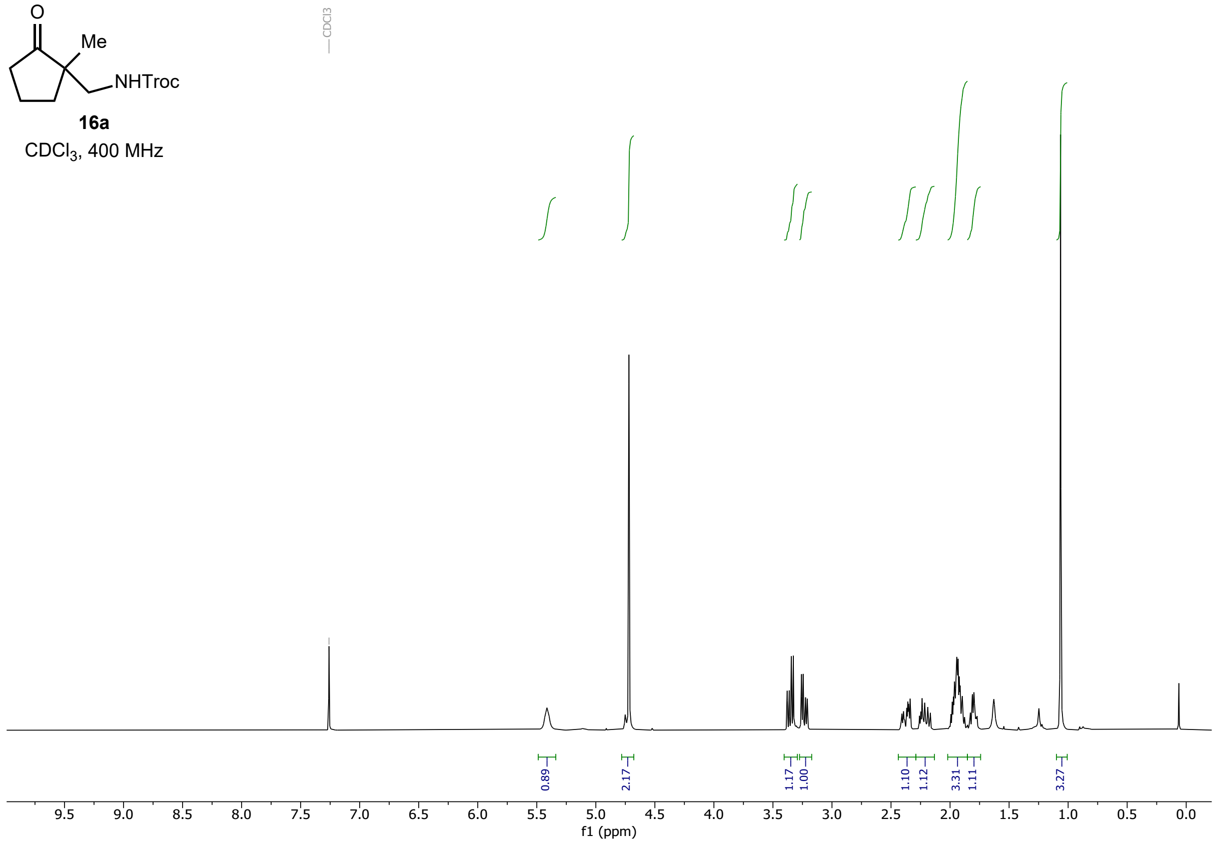
O
Me
NHTroc
16a
$CDCl_3$, 400 MHz
CDCl3
0.89
2.17
1.17
1.00
1.10
1.12
3.31
1.11
3.27
9.5 9.0 8.5 8.0 7.5 7.0 6.5 6.0 5.5 5.0 4.5 4.0 3.5 3.0 2.5 2.0 1.5 1.0 0.5 0.0
f1 (ppm)

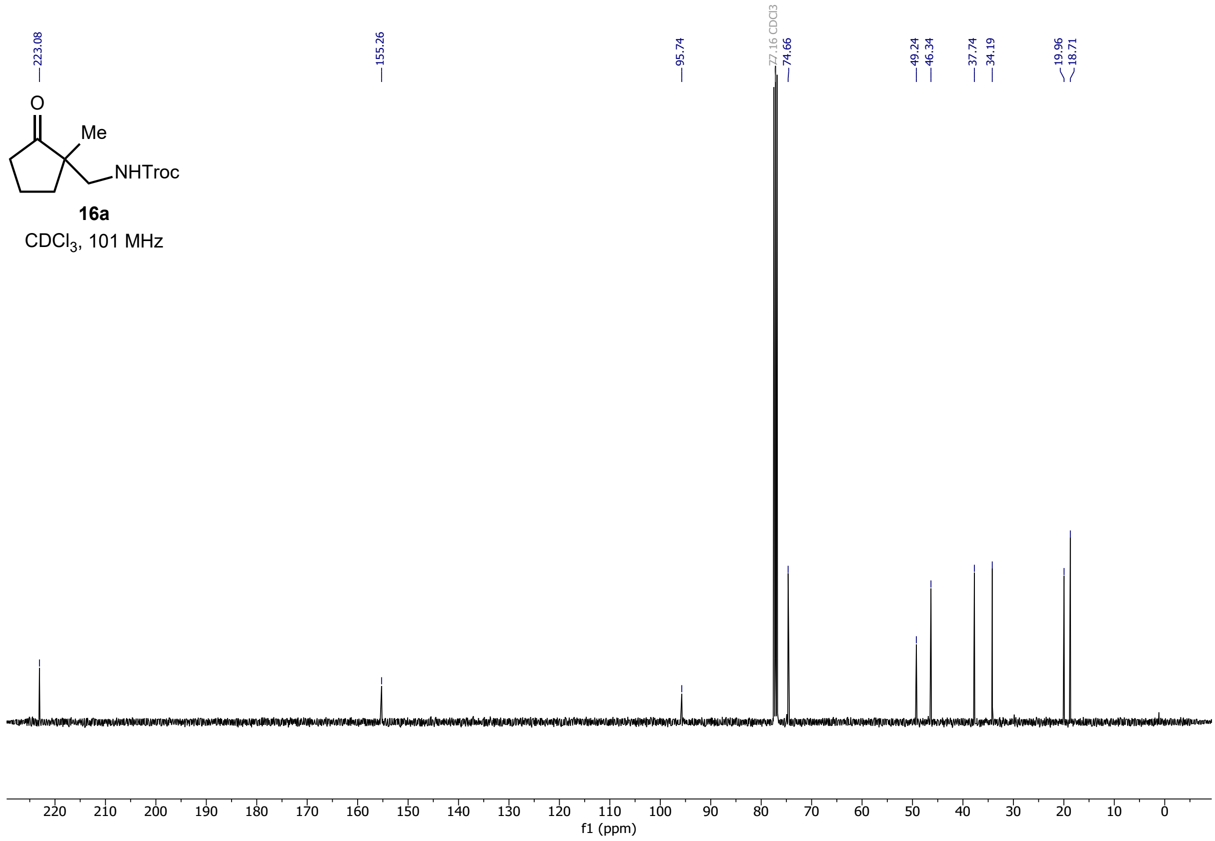
223.08
155.26
95.74
77.16 CDCl3
74.66
49.24
46.34
37.74
34.19
19.96
18.71
O
Me
NHTroc
16a
$CDCl_3$, 101 MHz
220 210 200 190 180 170 160 150 140 130 120 110 100 90 80 70 60 50 40 30 20 10 0
f1 (ppm)

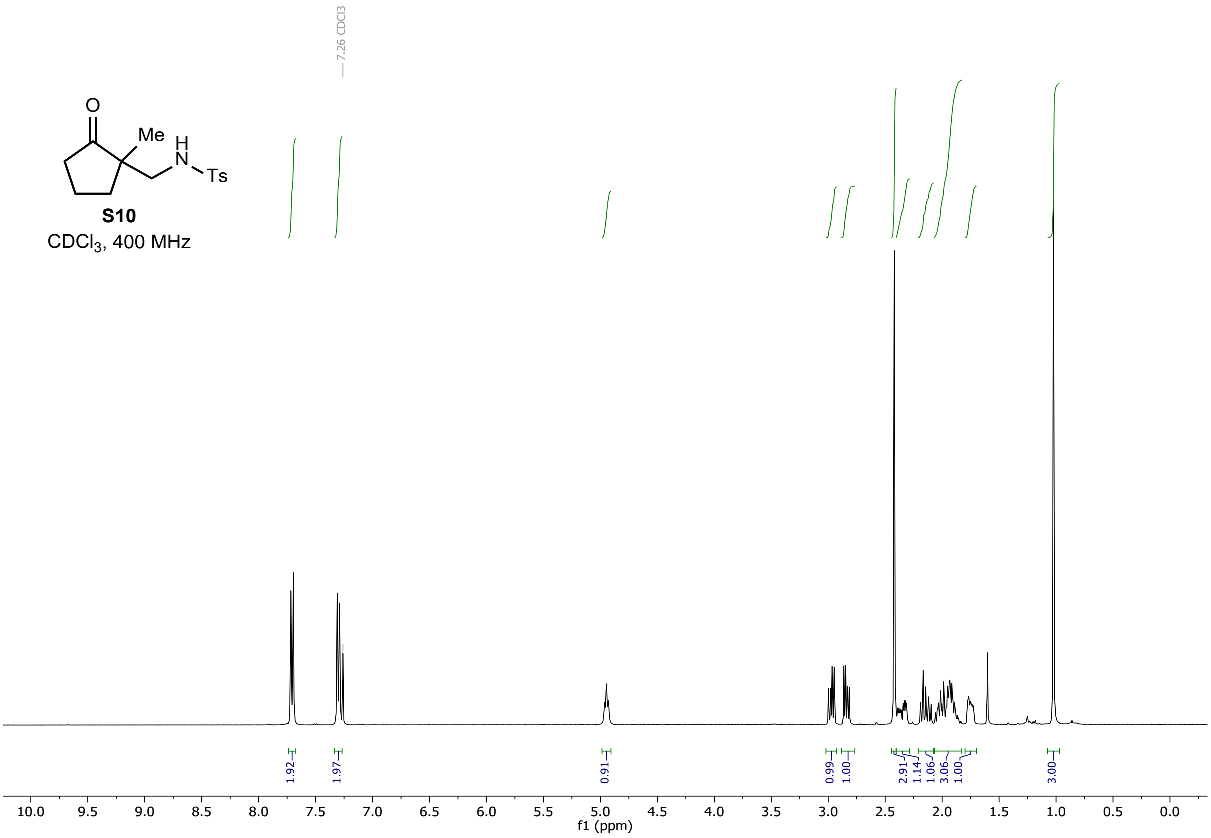

7.26 CDCl3
S10
$CDCl_3$, 400 MHz
1.92
1.97
0.91
0.99
1.00
2.91
1.14
1.06
3.06
1.00
3.00
f1 (ppm)

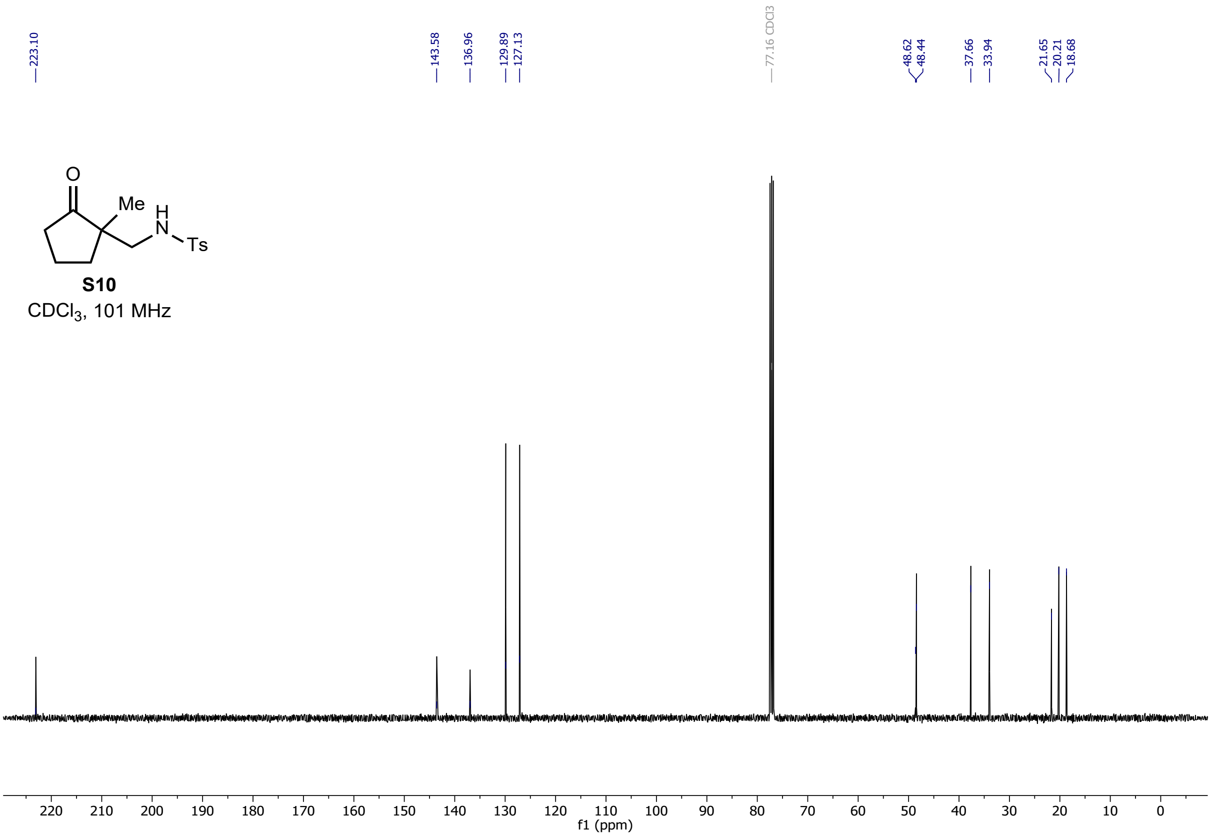

223.10
143.58
136.96
129.89
127.13
77.16 CDCl3
48.62
48.44
37.66
33.94
21.65
20.21
18.68
S10
$CDCl_3$, 101 MHz
f1 (ppm)

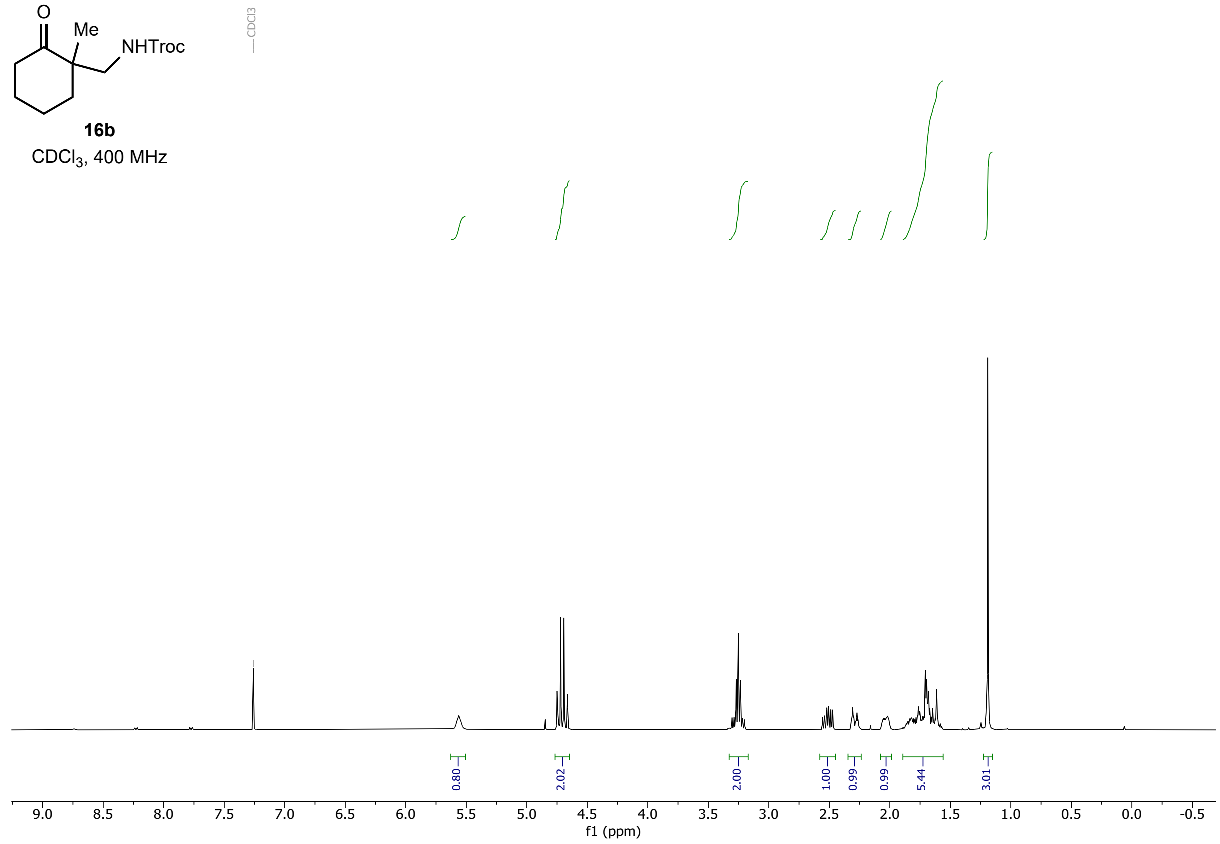
O
Me
NHTroc
16b
CDCl3, 400 MHz
CDCl3
0.80
2.02
2.00
1.00
0.99
0.99
5.44
3.01
9.0 8.5 8.0 7.5 7.0 6.5 6.0 5.5 5.0 4.5 4.0 3.5 3.0 2.5 2.0 1.5 1.0 0.5 0.0 -0.5
f1 (ppm)

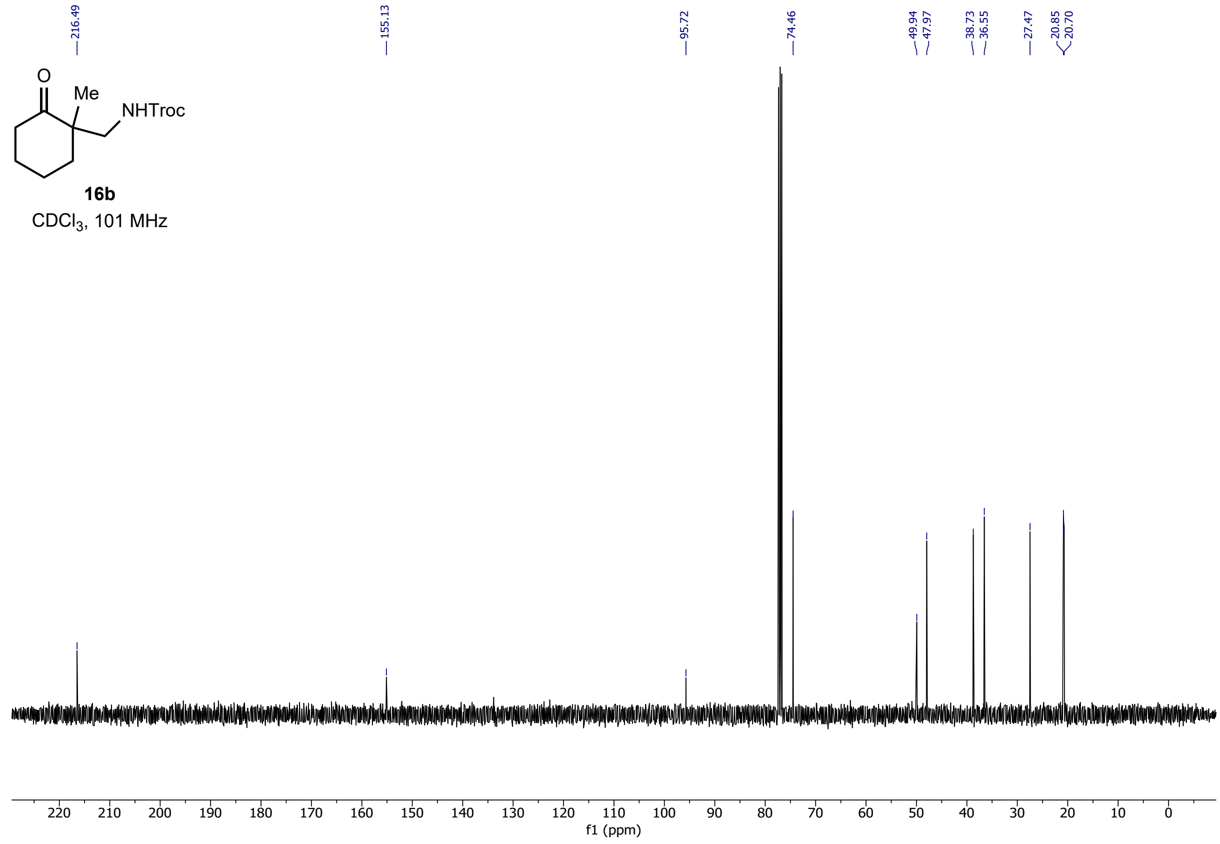
216.49
155.13
95.72
74.46
49.94
47.97
38.73
36.55
27.47
20.85
20.70
O
Me
NHTroc
16b
CDCl3, 101 MHz
220 210 200 190 180 170 160 150 140 130 120 110 100 90 80 70 60 50 40 30 20 10 0
f1 (ppm)

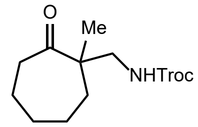

16c
$CDCl_3$, 500 MHz

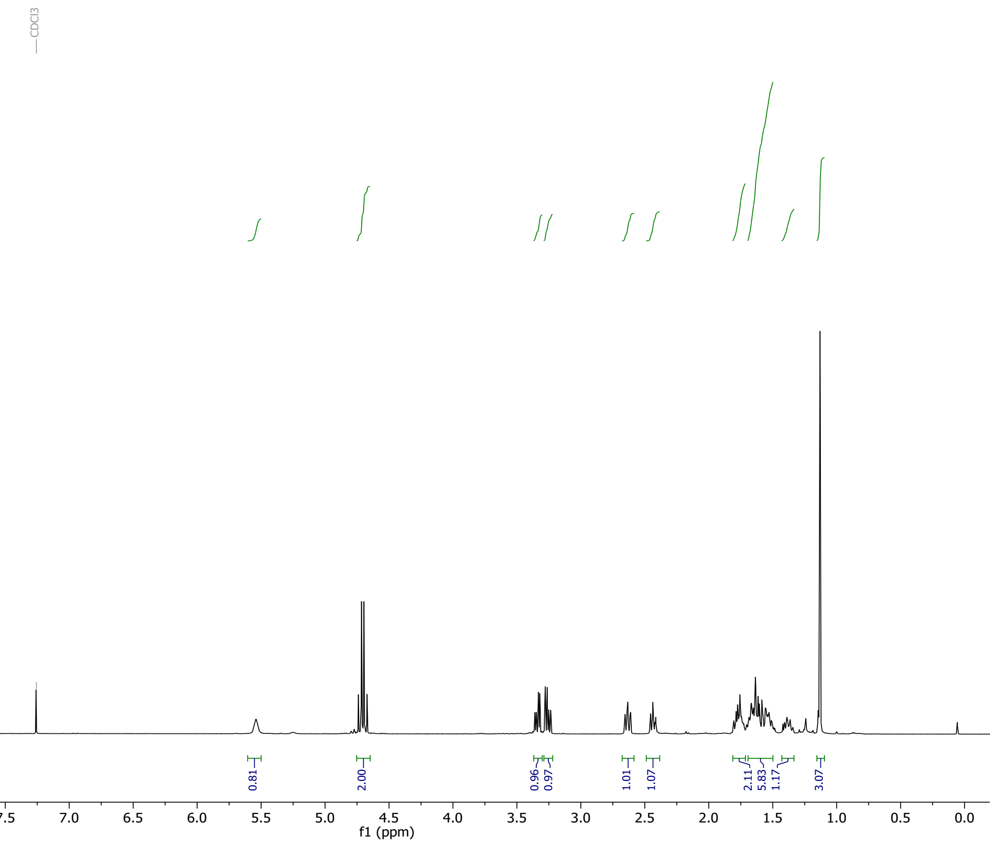

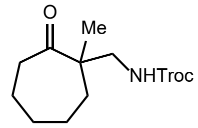

16c
$CDCl_3$, 126 MHz

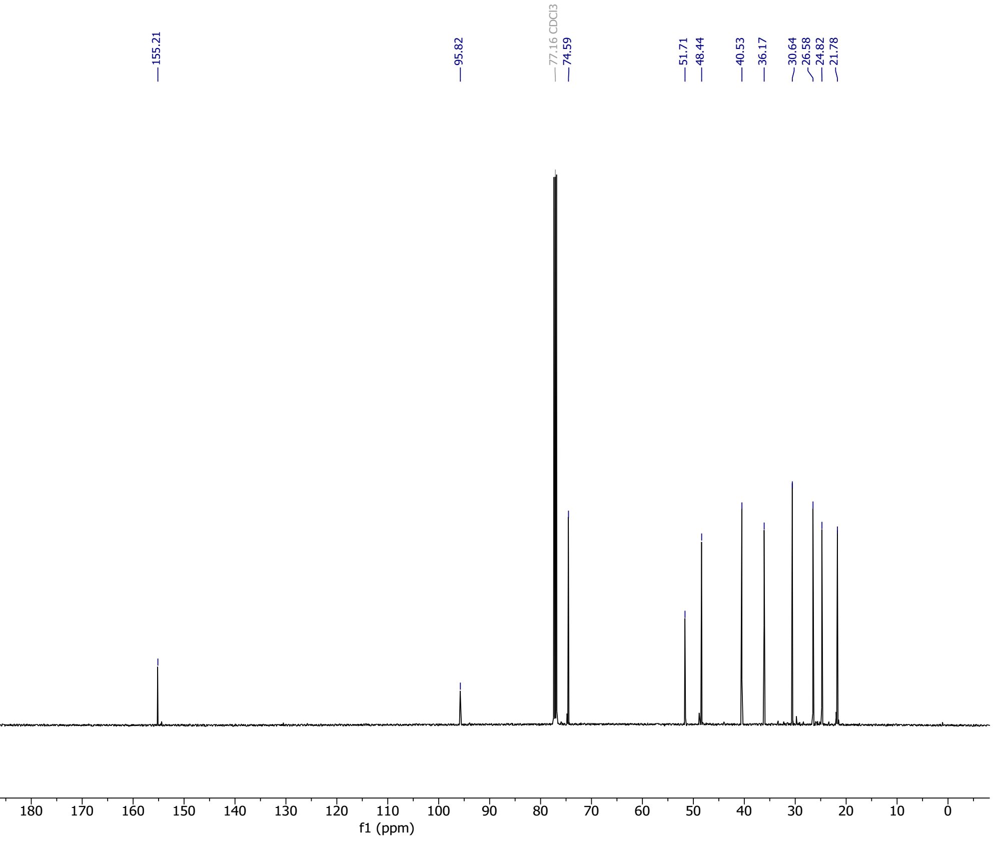

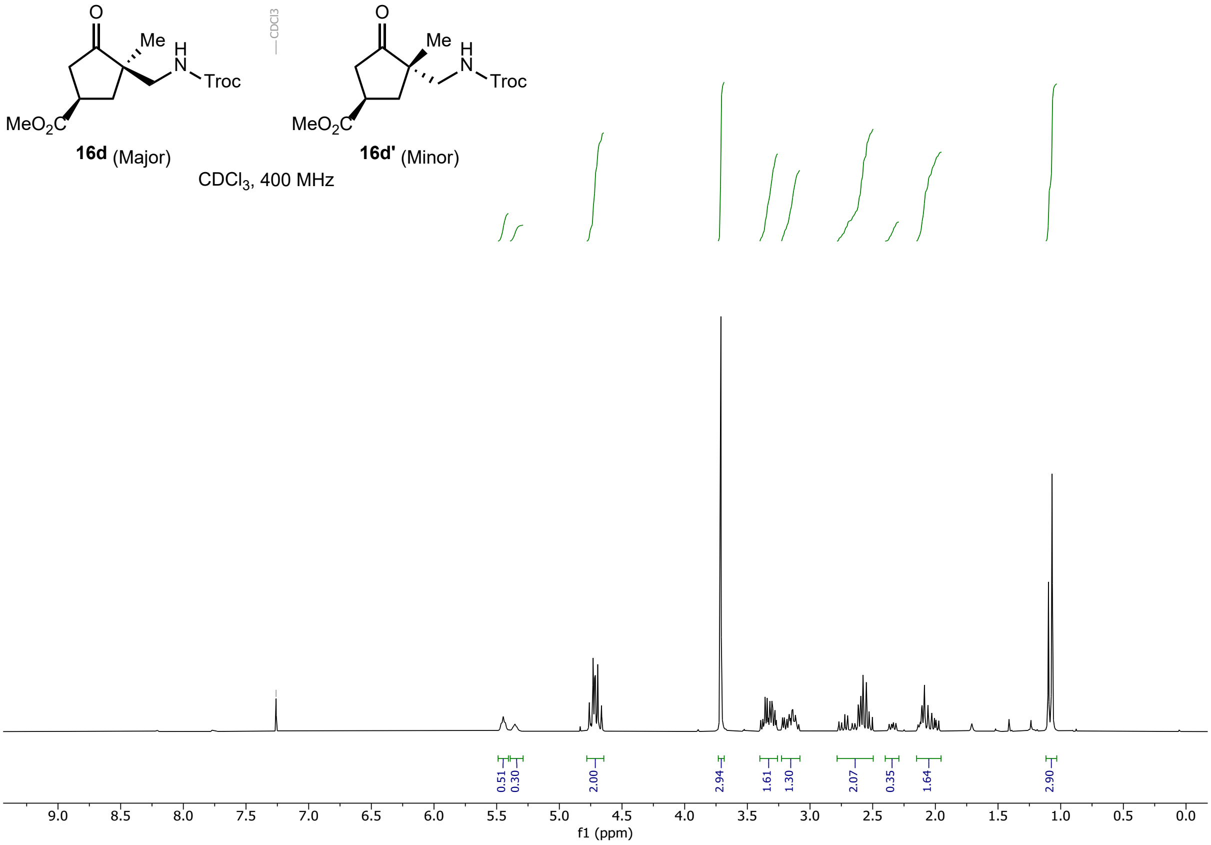
16d (Major)
16d' (Minor)
CDCl3, 400 MHz
0.51 0.30 2.00 2.94 1.61 1.30 2.07 0.35 1.64 2.90
f1 (ppm)

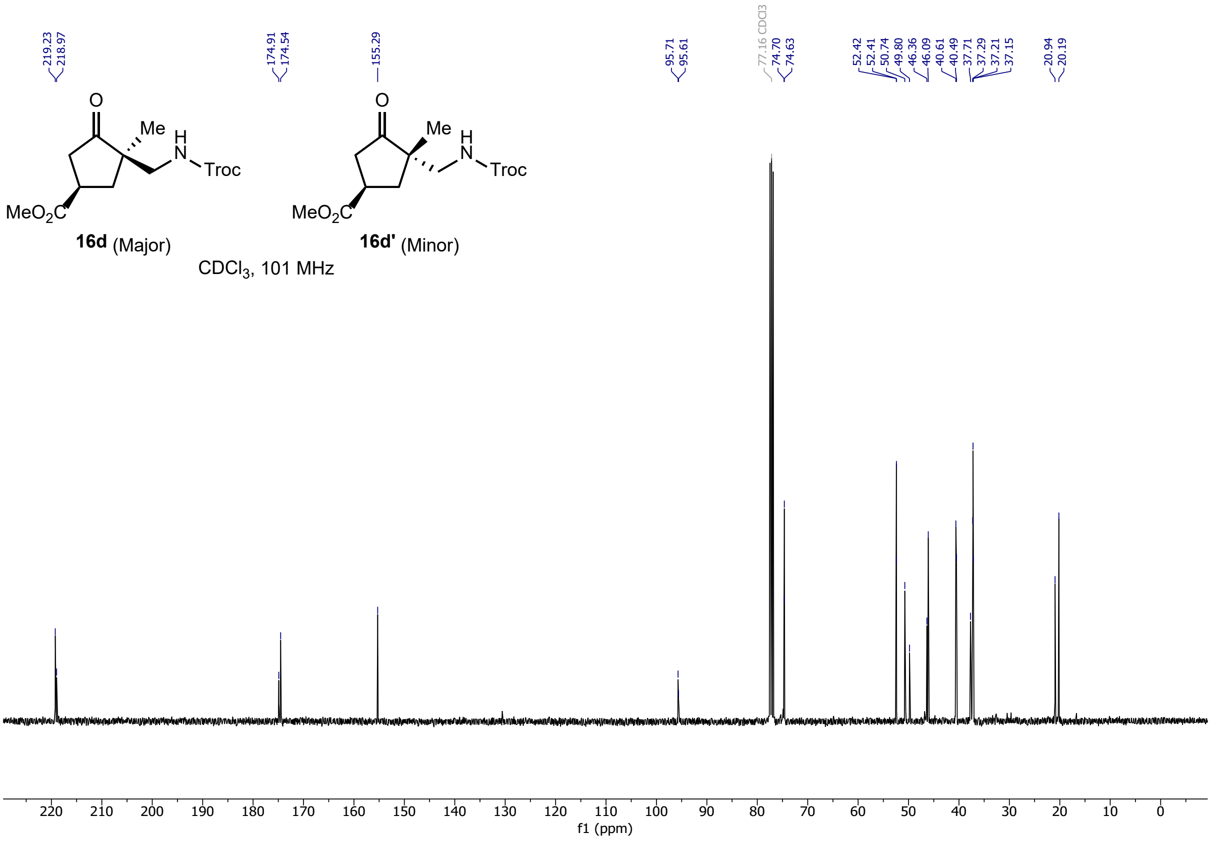
219.23 218.97
174.91 174.54
155.29
95.71 95.61
77.16 CDCl3
74.70 74.63
52.42 52.41 50.74 49.80 46.36 46.09 40.61 40.49 37.71 37.29 37.21 37.15
20.94 20.19
16d (Major)
16d' (Minor)
CDCl3, 101 MHz
f1 (ppm)

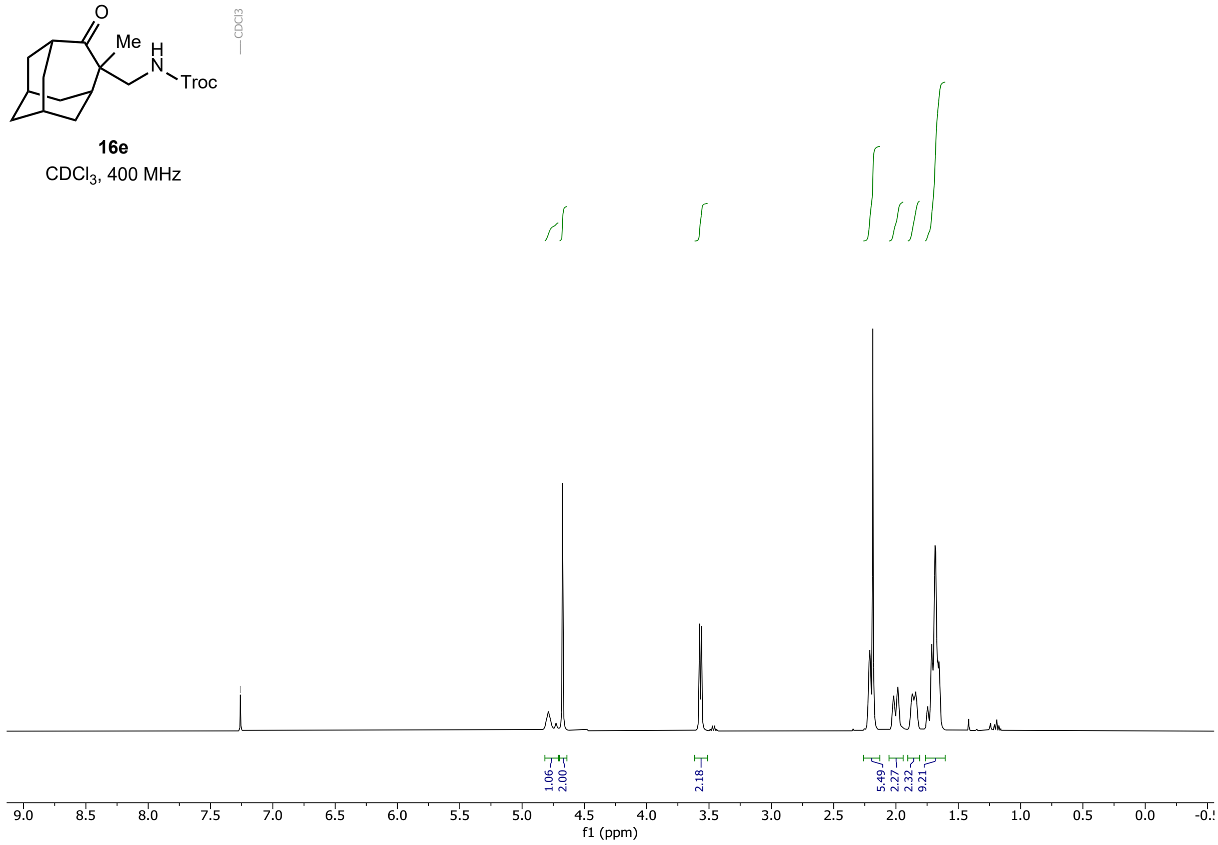
Me
H
N
Troc
O
16e
CDCl3, 400 MHz
CDCl3
1.06
2.00
2.18
5.49
2.27
2.32
9.21
f1 (ppm)

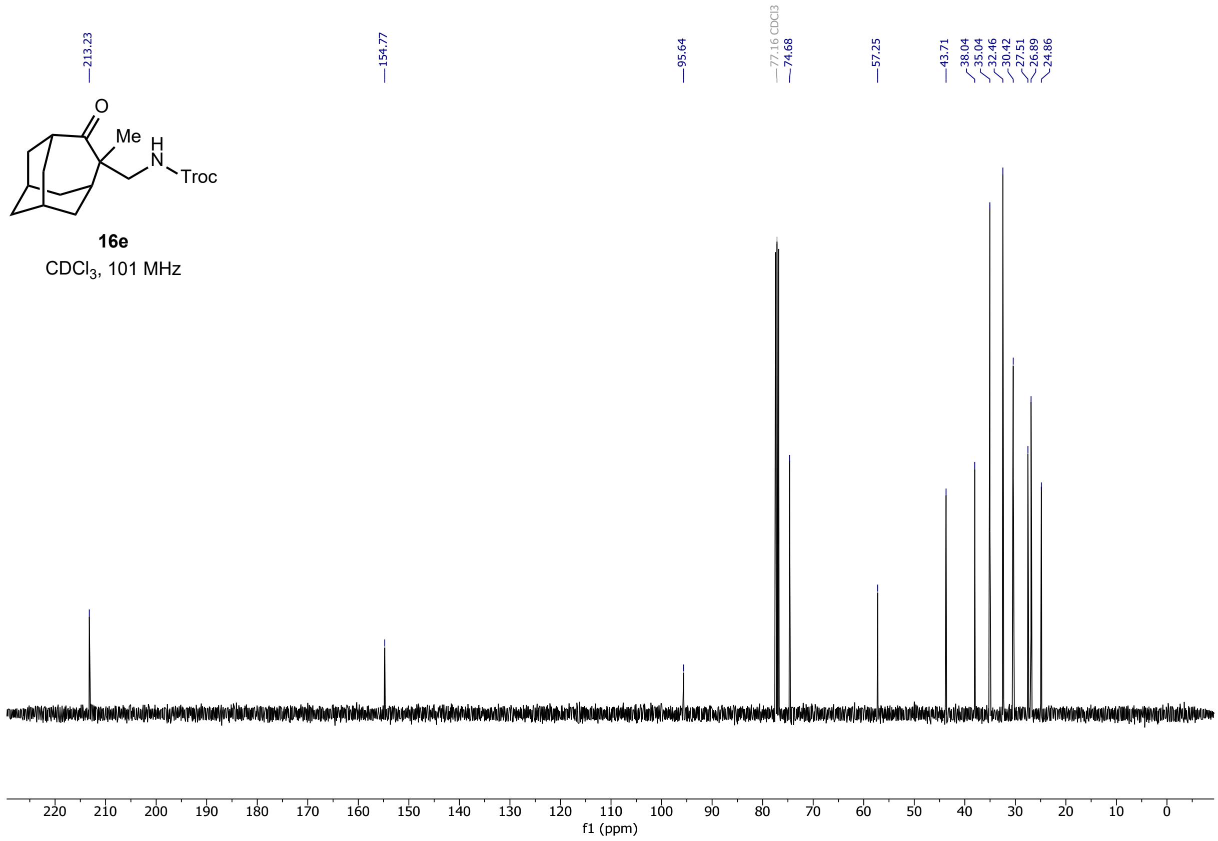
213.23
154.77
95.64
77.16 CDCl3
74.68
57.25
43.71
38.04
35.04
32.46
30.42
27.51
26.89
24.86
Me
H
N
Troc
O
16e
CDCl3, 101 MHz
f1 (ppm)

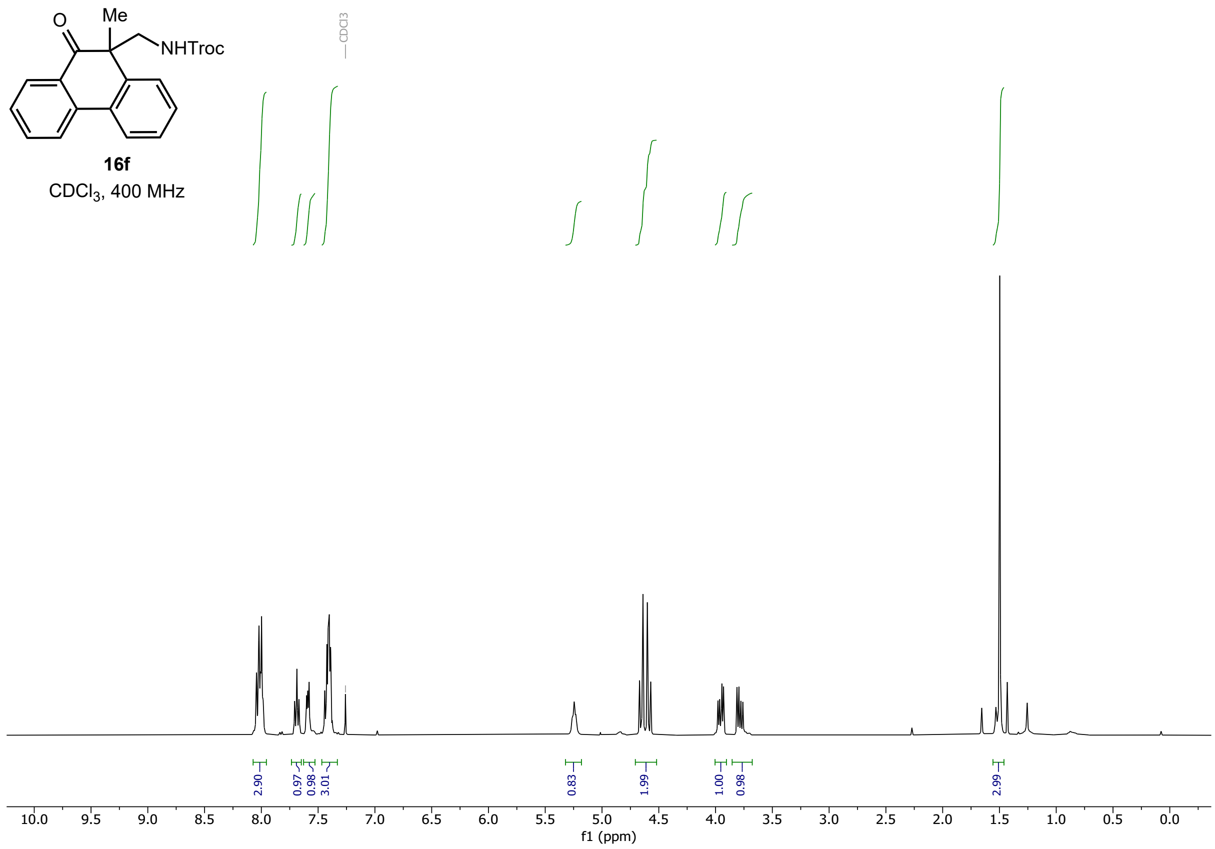
Me
O
NHTroc
16f
CDCl3, 400 MHz
CDCl3
2.90
0.97
0.98
3.01
0.83
1.99
1.00
0.98
2.99
f1 (ppm)

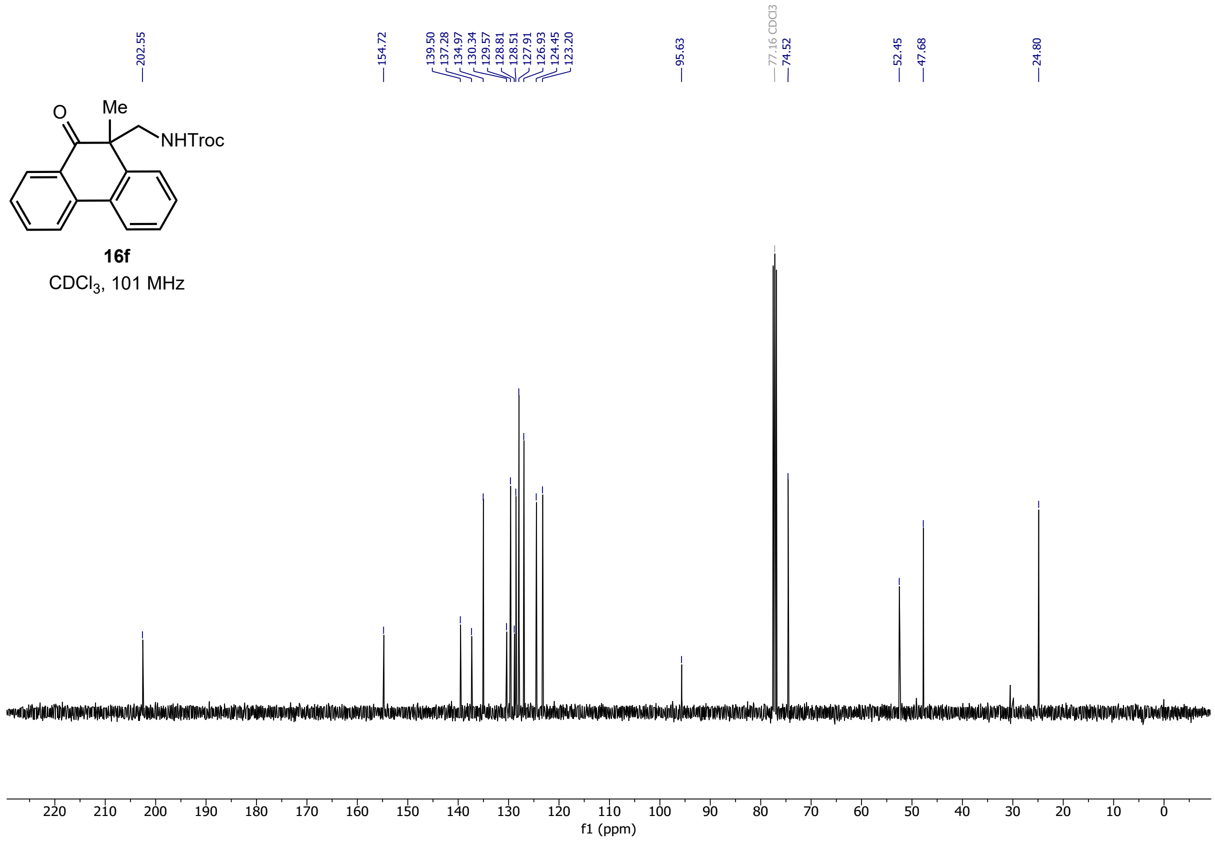
Me
O
NHTroc
16f
CDCl3, 101 MHz
202.55
154.72
139.50
137.28
134.97
130.34
129.57
128.81
128.51
127.91
126.93
124.45
123.20
95.63
77.16 CDCl3
74.52
52.45
47.68
24.80
f1 (ppm)

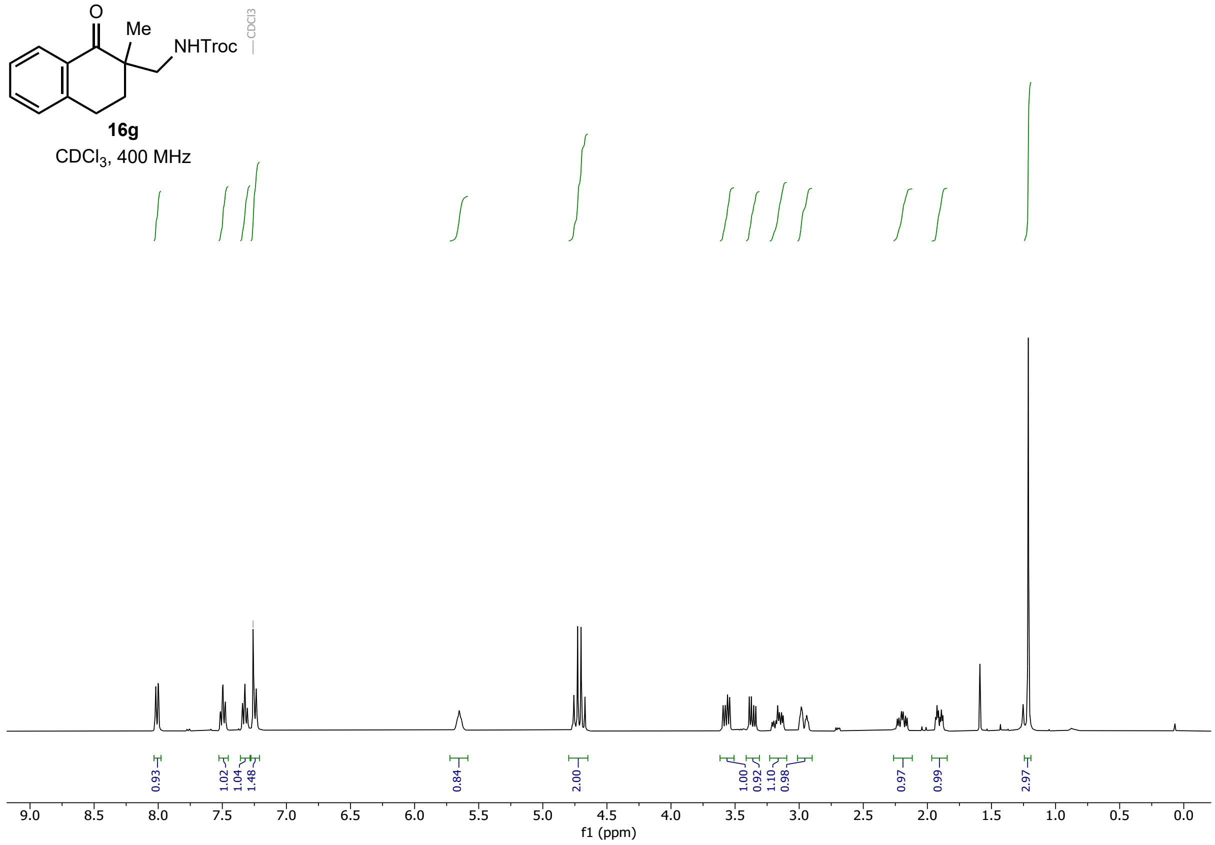
O
Me
NHTroc
16g
CDCl3, 400 MHz
CDCl3
0.93
1.02
1.04
1.48
0.84
2.00
1.00
0.92
1.10
0.98
0.97
0.99
2.97
9.0 8.5 8.0 7.5 7.0 6.5 6.0 5.5 5.0 4.5 4.0 3.5 3.0 2.5 2.0 1.5 1.0 0.5 0.0
f1 (ppm)

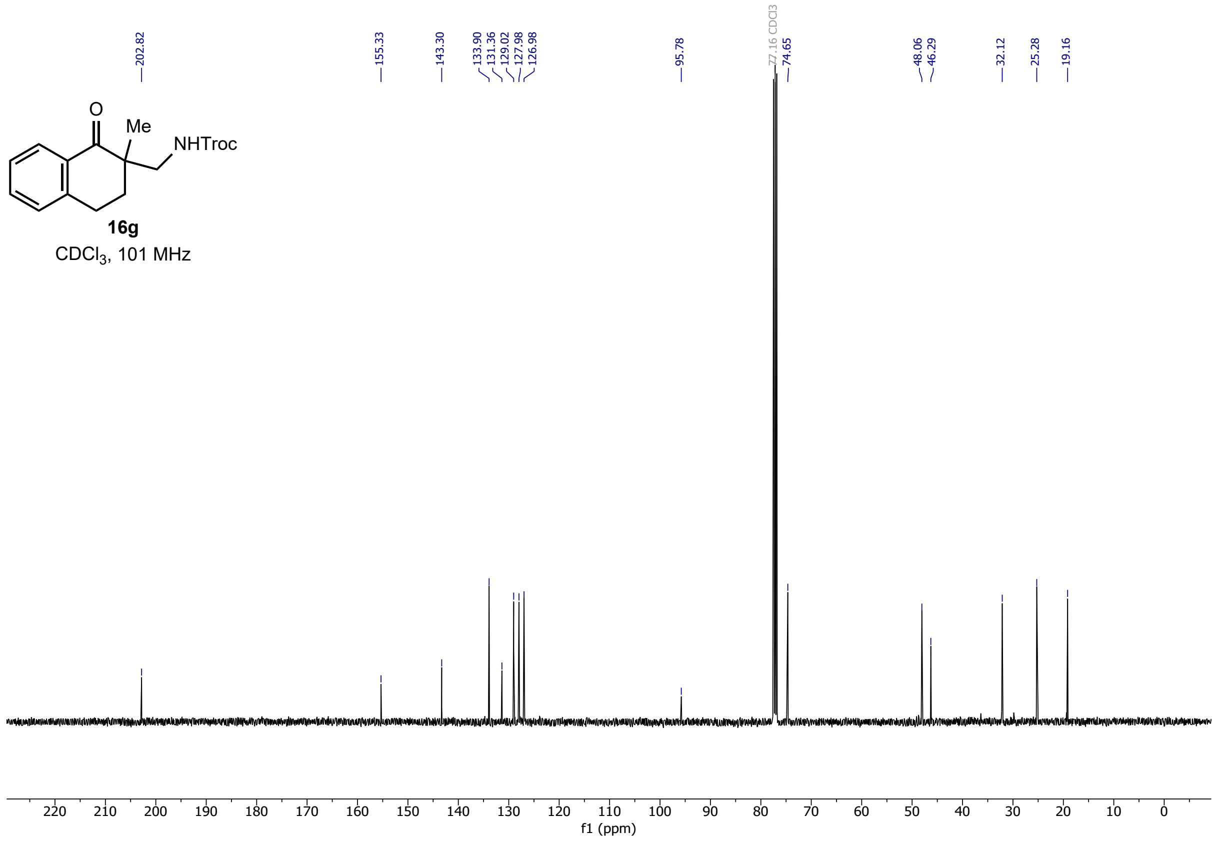
202.82
155.33
143.30
133.90
131.36
129.02
127.98
126.98
95.78
77.16 CDCl3
74.65
48.06
46.29
32.12
25.28
19.16
O
Me
NHTroc
16g
CDCl3, 101 MHz
220 210 200 190 180 170 160 150 140 130 120 110 100 90 80 70 60 50 40 30 20 10 0
f1 (ppm)

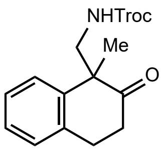

16g'
$CDCl_3$, 400 MHz

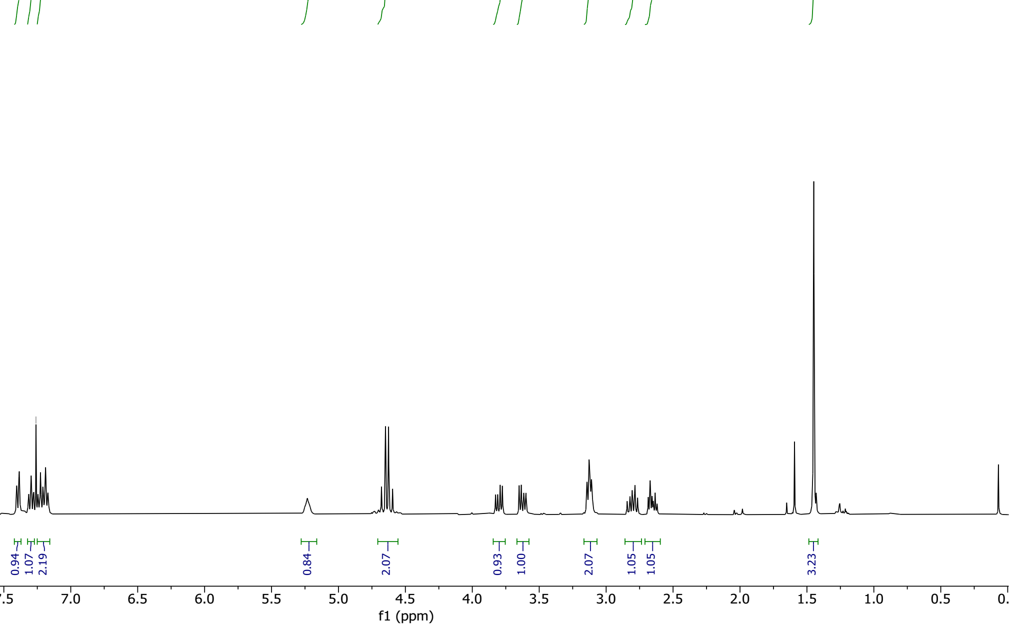

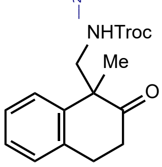

16g'
$CDCl_3$, 101 MHz

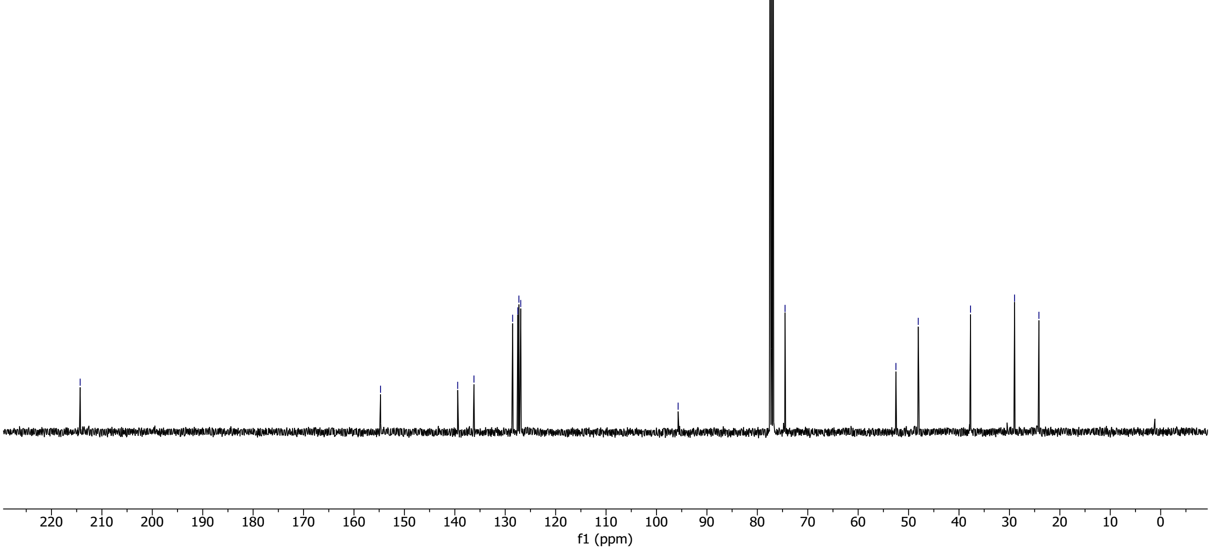

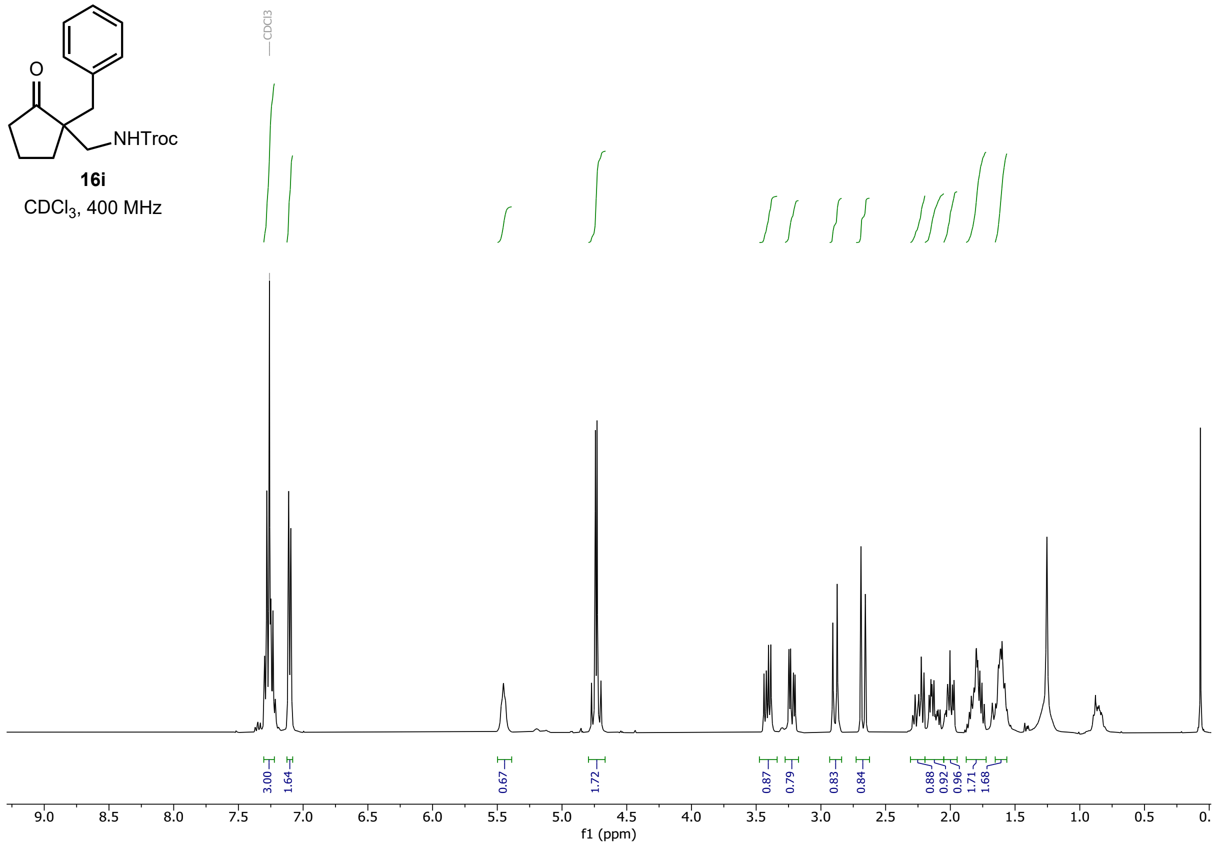
NHTroc
O
16i
$CDCl_3$, 400 MHz
CDCl3
3.00
1.64
0.67
1.72
0.87
0.79
0.83
0.84
0.88
0.92
0.96
1.71
1.68
f1 (ppm)

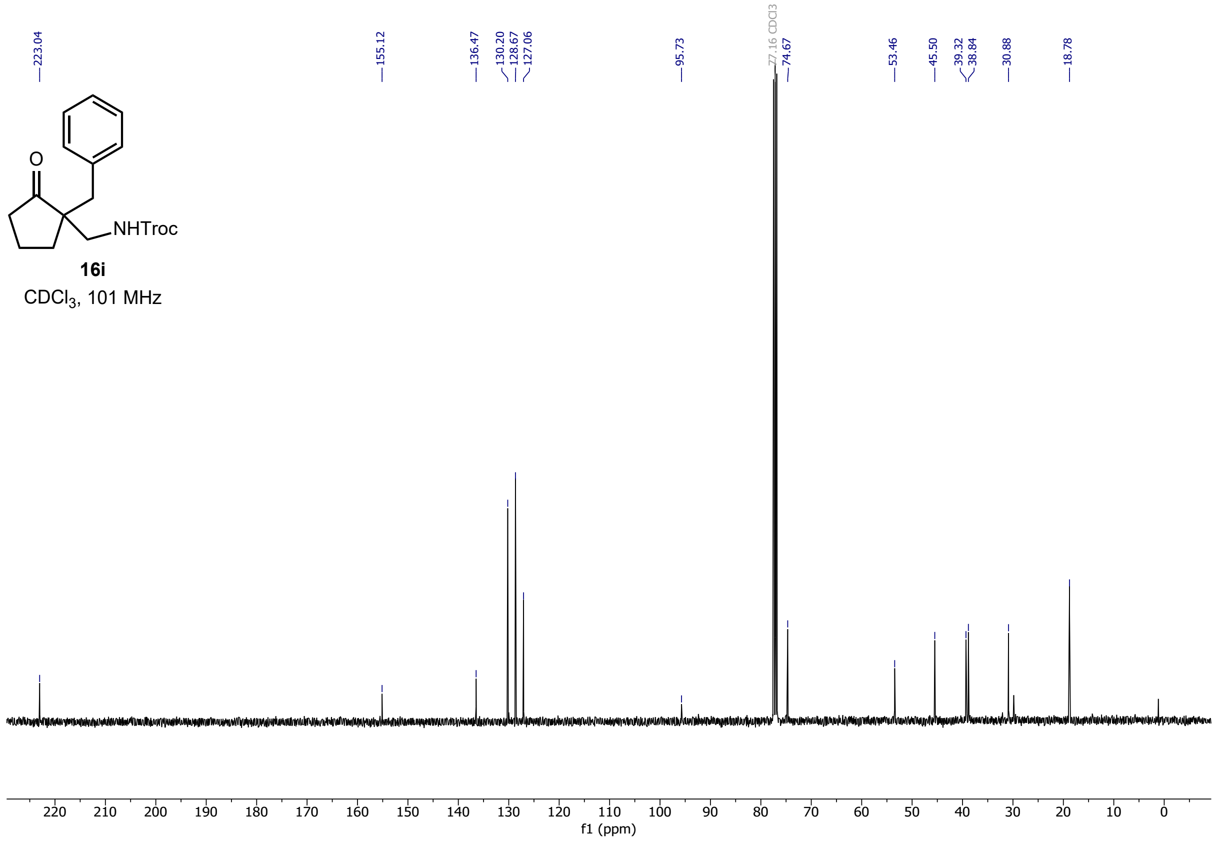
223.04
155.12
136.47
130.20
128.67
127.06
95.73
77.16 CDCl3
74.67
53.46
45.50
39.32
38.84
30.88
18.78
NHTroc
O
16i
$CDCl_3$, 101 MHz
f1 (ppm)

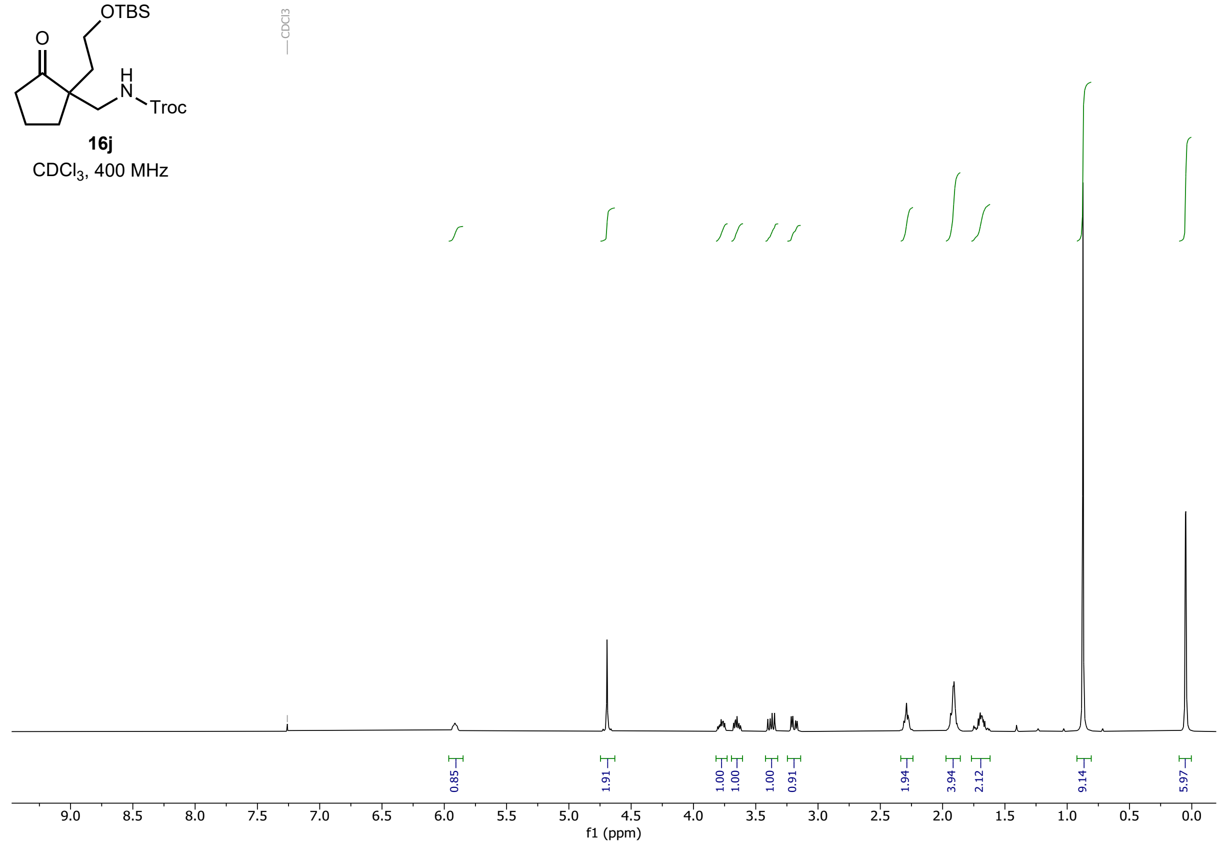
OTBS
O
H
N
Troc
16j
$CDCl_3$, 400 MHz
CDCl3
0.85
1.91
1.00
1.00
1.00
0.91
1.94
3.94
2.12
9.14
5.97
9.0 8.5 8.0 7.5 7.0 6.5 6.0 5.5 5.0 4.5 4.0 3.5 3.0 2.5 2.0 1.5 1.0 0.5 0.0
f1 (ppm)

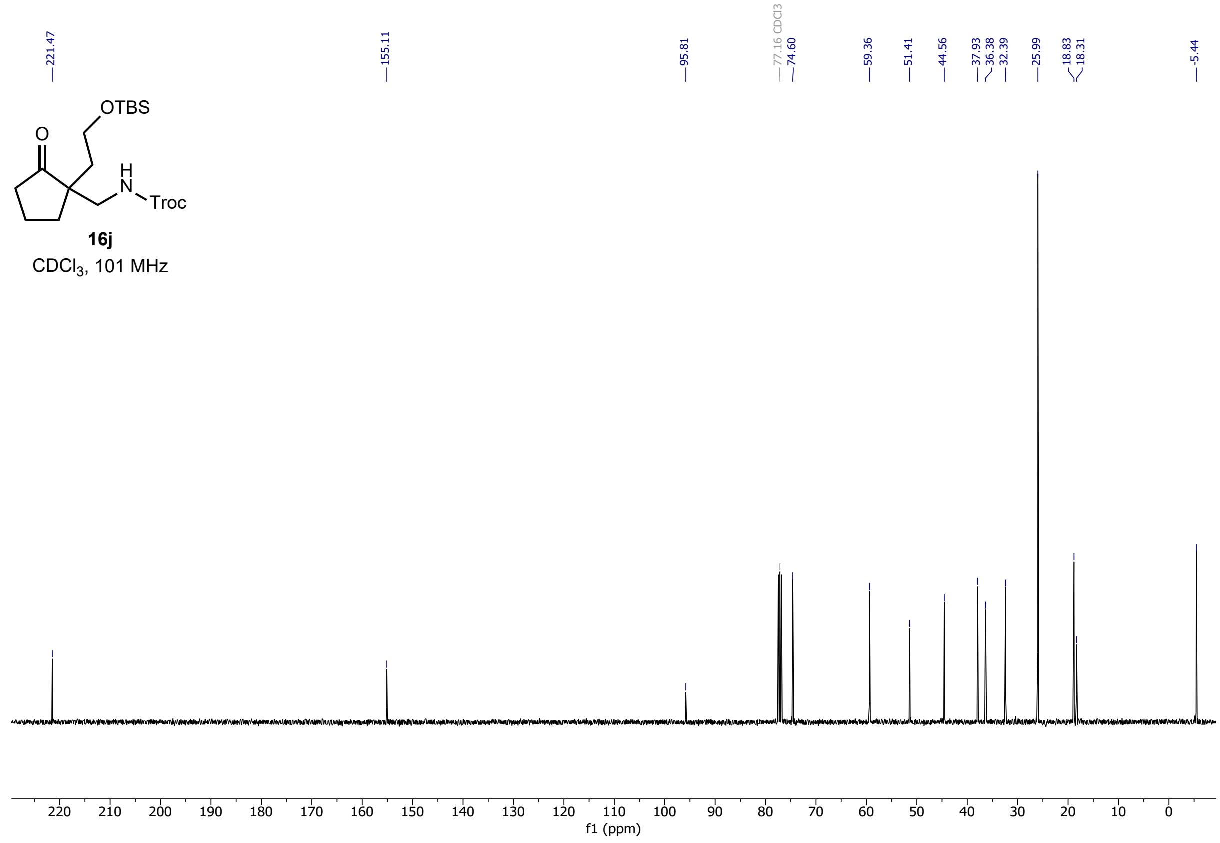
221.47
155.11
95.81
77.16 CDCl3
74.60
59.36
51.41
44.56
37.93
36.38
32.39
25.99
18.83
18.31
-5.44
OTBS
O
H
N
Troc
16j
$CDCl_3$, 101 MHz
220 210 200 190 180 170 160 150 140 130 120 110 100 90 80 70 60 50 40 30 20 10 0
f1 (ppm)

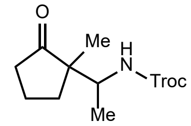

16k (Major)
$CDCl_3$, 400 MHz

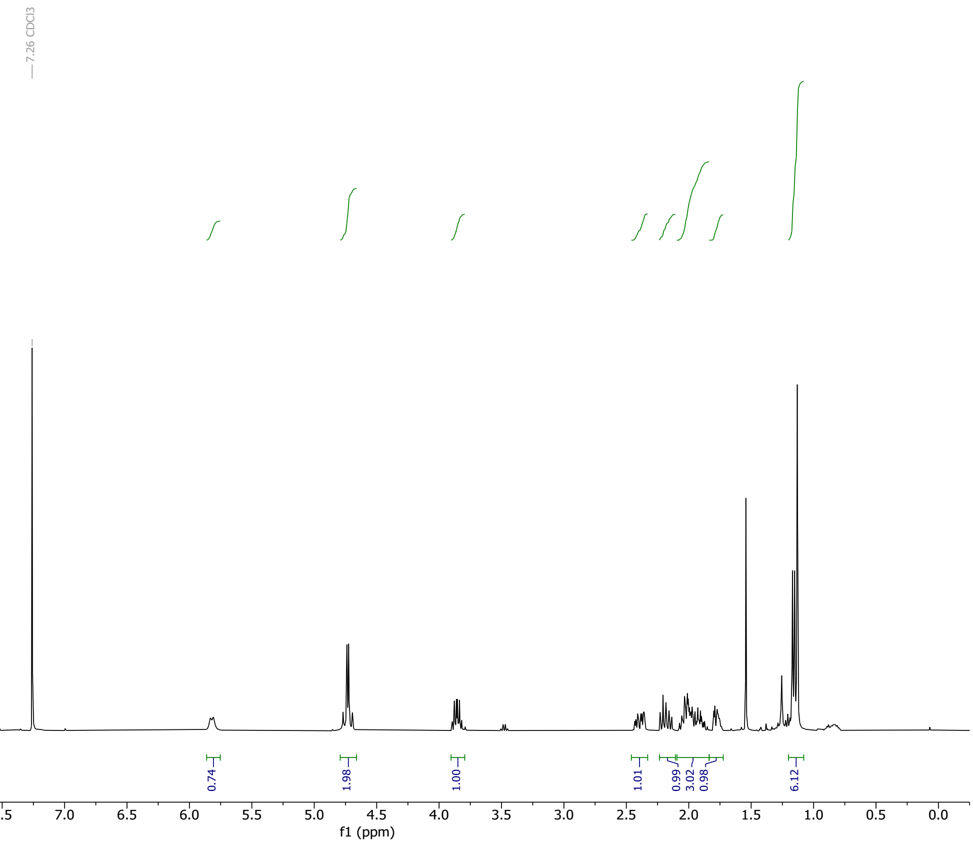

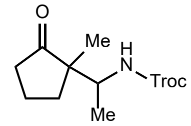

16k (Major)
$CDCl_3$, 101 MHz

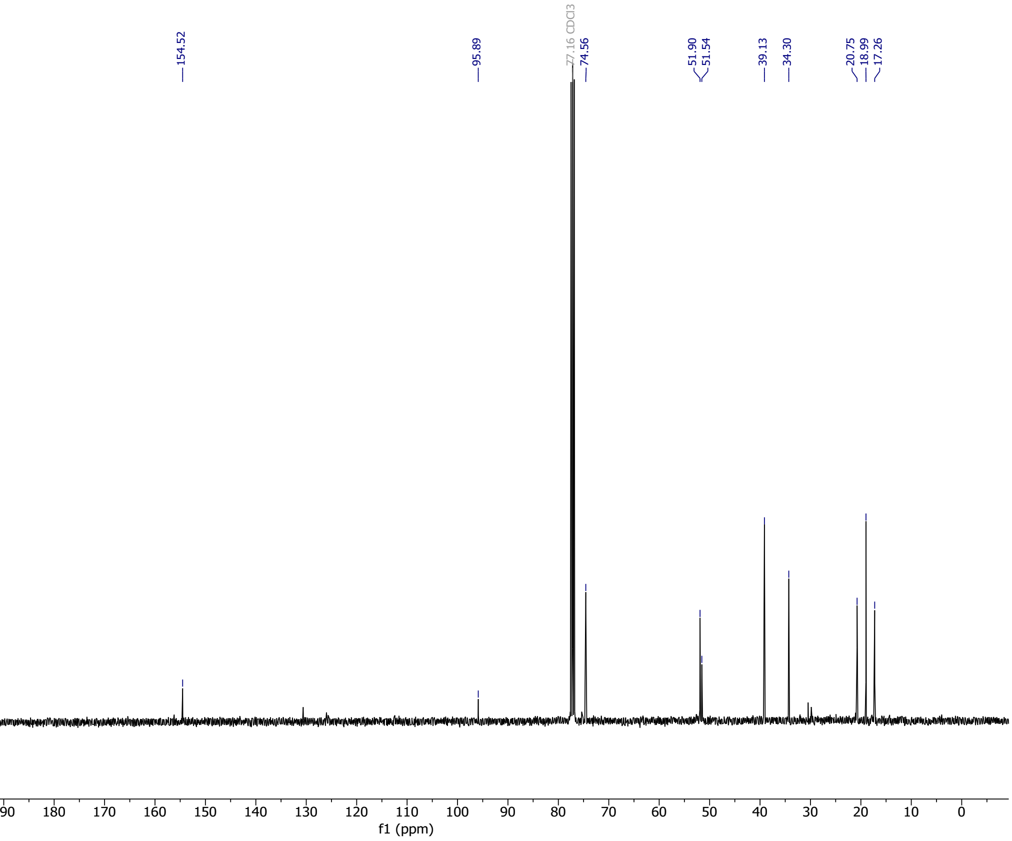

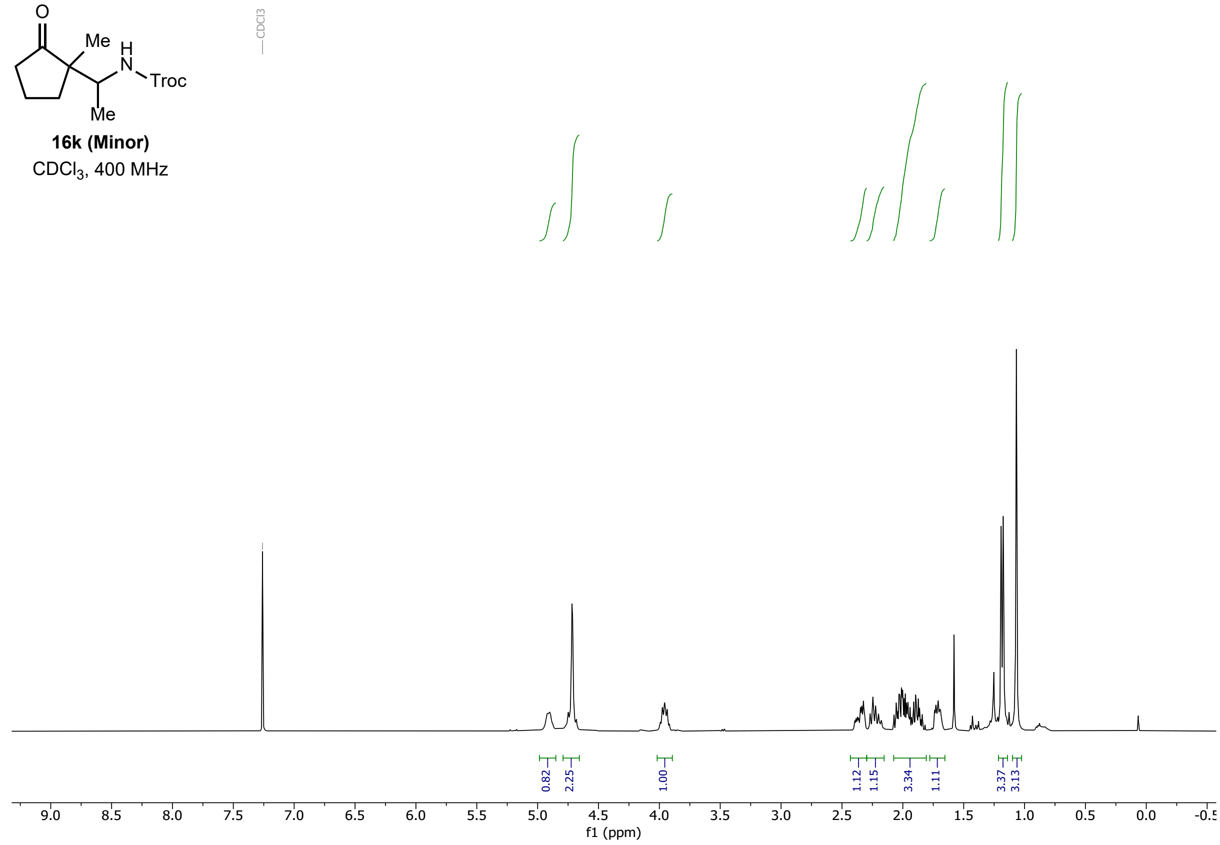

O
Me
H
N
Troc
Me
16k (Minor)
CDCl3, 400 MHz
CDCl3
0.82
2.25
1.00
1.12
1.15
3.34
1.11
3.37
3.13
f1 (ppm)

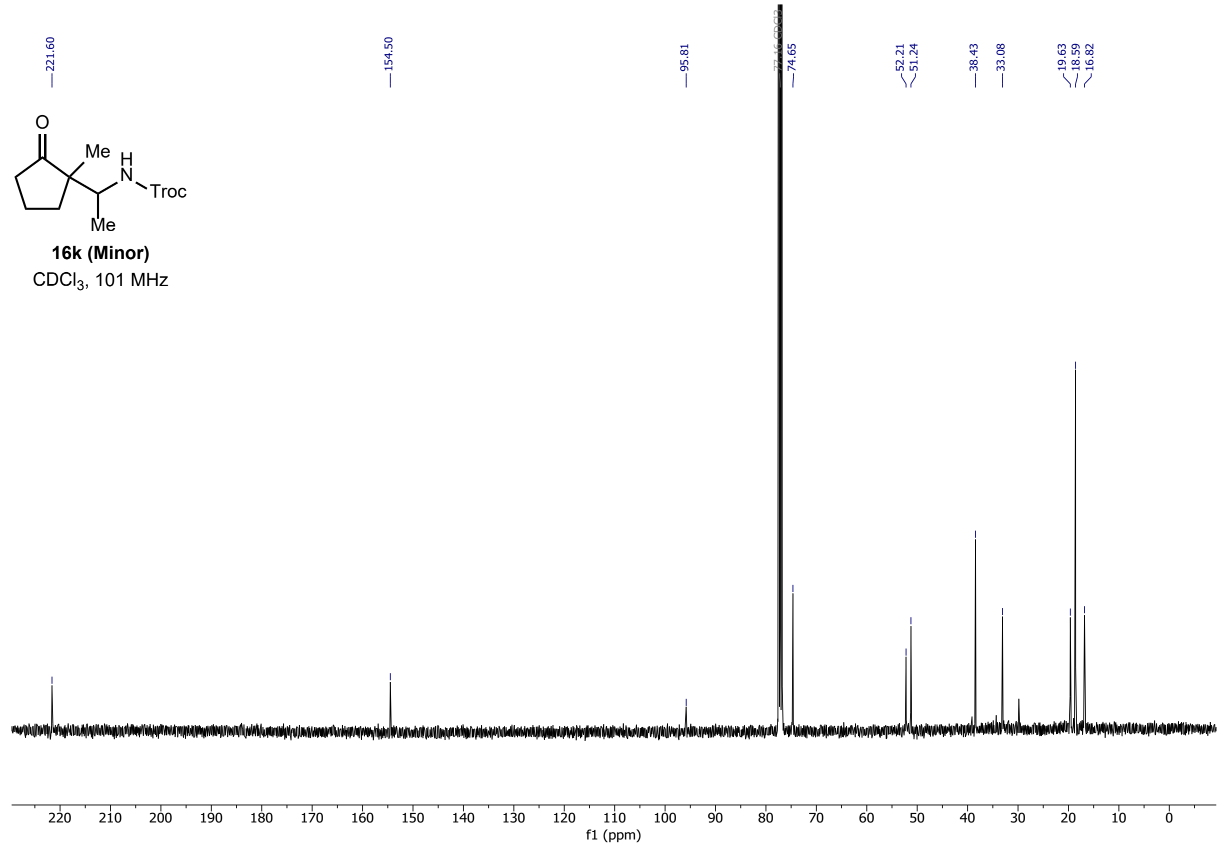

221.60
154.50
95.81
74.65
52.21
51.24
38.43
33.08
19.63
18.59
16.82
O
Me
H
N
Troc
Me
16k (Minor)
CDCl3, 101 MHz
f1 (ppm)

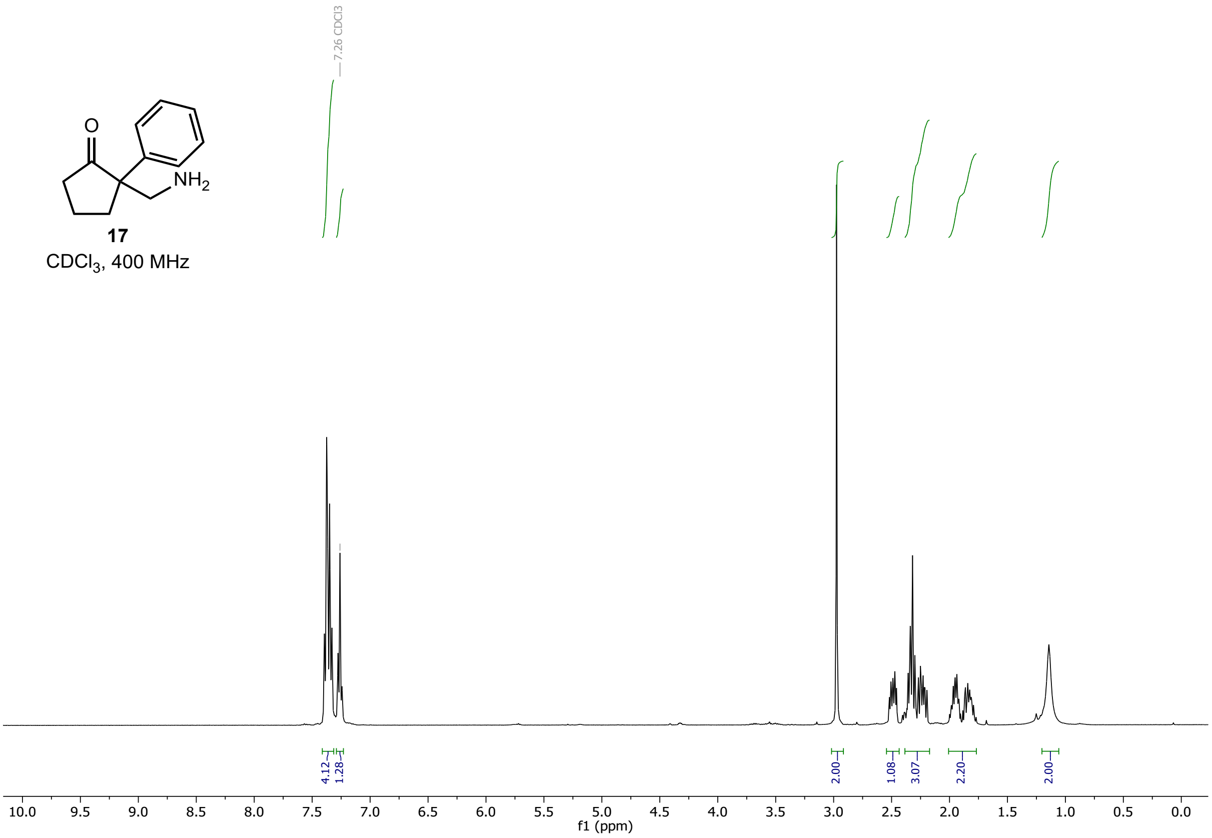
O
NH2
17
CDCl3, 400 MHz
7.26 CDCl3
4.12
1.28
2.00
1.08
3.07
2.20
2.00
f1 (ppm)

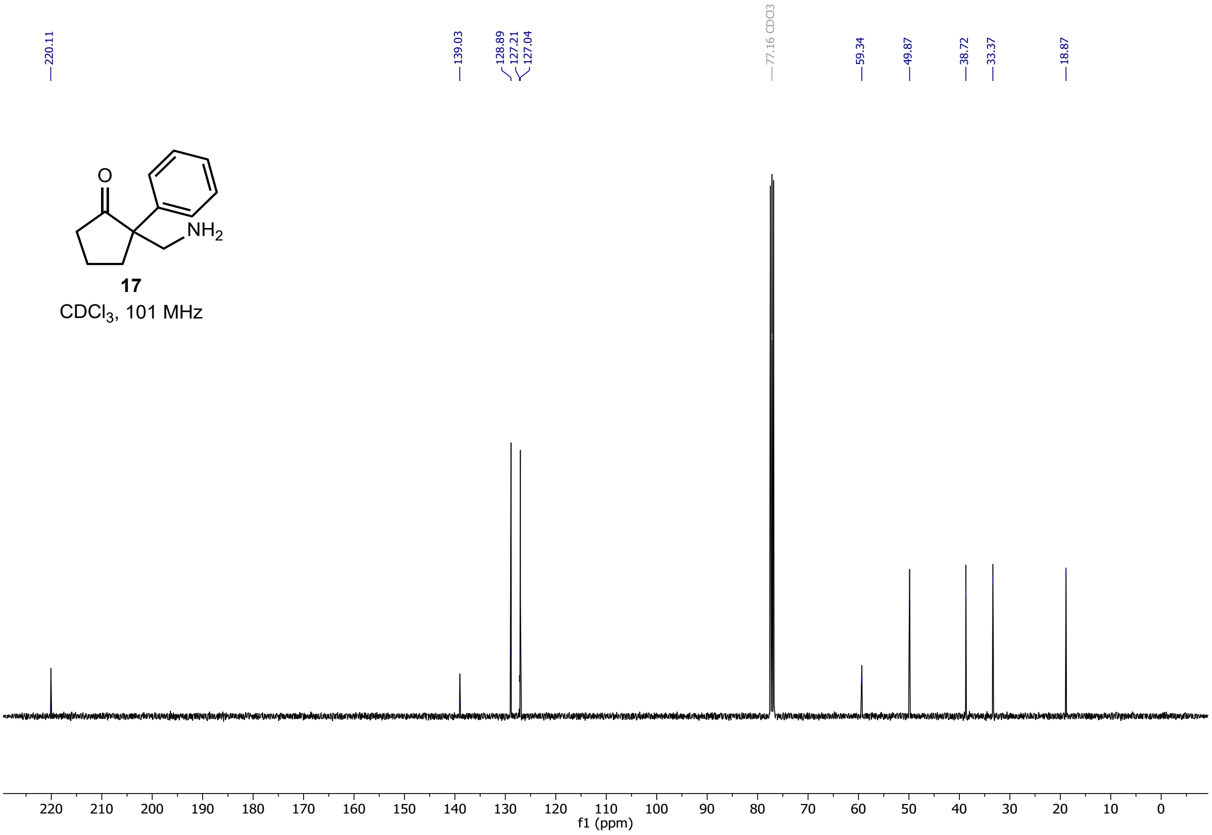
220.11
139.03
128.89
127.21
127.04
77.16 CDCl3
59.34
49.87
38.72
33.37
18.87
O
NH2
17
CDCl3, 101 MHz
f1 (ppm)
